# Machine Learning for Clinical Outcome Prediction After Transcatheter Aortic Valve Replacement: A Systematic Review and Meta-Analysis of Predictive Performance, Validation, and Clinical Readiness

**DOI:** 10.3390/jcm15197692

**Published:** 2026-10-04

**Authors:** Azin Alizadehasl, Elahe Abdi Bastami, Seyedeh Fatemeh Hosseini Jebelli, Delaram Narimani Davani, Alia Bahramnejad, Azam Yalameh Aliabadi, Seyyedeh Maryam Moussavi, Azin Ghaffari, Reza Amani-Beni, Bahar Darouei, Ehsan Amini-Salehi, Seyyed Mohammad Hashemi, Samira Golkar, Hoda Hakimian, Soroush Najdaghi

**Affiliations:** 1 Cardio-Oncology Research Center, Rajaie Cardiovascular Institute, Tehran 1995614331, Iran; 2 Neuroscience Research Center, Isfahan University of Medical Sciences, Isfahan 81746-73461, Iran; 3 School of Medicine and Dentistry, Griffith University, Queensland 4101, Australia; 4 Cardiovascular Research Center, Rajaie Cardiovascular Institute, Tehran 1996114151, Iran; 5 Heart Failure Research Center, Cardiovascular Research Institute, Isfahan University of Medical Sciences, Isfahan 81589-87944, Iran; 6 Gastrointestinal and Liver Diseases Research Center, Guilan University of Medical Sciences, Rasht 41625, Iran; 7 Cardiovascular Research Center, Hormozgan University of Medical Sciences, Bandar Abbas 7916193145, Iran; 8 Clinical Research Development Center, Najafabad Branch, Islamic Azad University, Najafabad 85135, Iran

**Keywords:** transcatheter aortic valve replacement, machine learning, artificial intelligence, risk assessment, treatment outcome, systematic review, meta-analysis

## Abstract

**Background:** Machine learning (ML) models are increasingly used to predict adverse outcomes after transcatheter aortic valve replacement (TAVR), but their generalizability and clinical readiness remain uncertain. We evaluated predictive performance across post-TAVR outcomes, emphasizing validation quality, transportability, methodological bias, and clinical utility. **Methods:** PubMed/MEDLINE, Embase, Scopus, and Web of Science were searched from inception through 6 August 2026 for supervised ML studies reporting out-of-sample performance for clinically relevant post-TAVR outcomes. Overlapping source populations were audited before synthesis. Areas under the receiver operating characteristic curve (AUCs) were pooled on the logit scale using restricted maximum likelihood (REML) random-effects models with modified Hartung–Knapp inference and prediction intervals. Prespecified analyses examined validation maturity, predictor timing, prediction horizon, data architecture, and risk of bias. Within-study dependence was addressed using multilevel meta-regression with bias-reduced cluster-robust variance estimation (CR2); Bayesian analyses complemented the primary synthesis. **Results:** Sixty-seven full-text reports were included. Twenty independent source/report families contributed to mortality meta-analysis, yielding a pooled AUC of 0.791 (95% confidence interval [CI], 0.732–0.840; I^2^ = 91.5%; prediction interval, 0.480–0.940). The high heterogeneity and wide prediction interval indicate that this pooled AUC is an average across heterogeneous settings rather than a transportable performance guarantee. Stronger external, temporal, geographic, or inter-centre evaluation showed lower discrimination than internal/resampled evaluation (0.754 vs. 0.814; *p* = 0.299), while preprocedural models yielded 0.767 versus 0.866 for peri-/postprocedural or mixed models (*p* = 0.083). High-risk studies showed higher apparent mortality discrimination than low/unclear-risk studies (0.895 vs. 0.747; *p* for interaction = 0.0009); this observational comparison involved only five high-risk mortality studies and should not be interpreted causally. The Bayesian-adjusted difference was +0.118 AUC units (95% credible interval [CrI], +0.037 to +0.179). Pooled AUCs were 0.760 for permanent pacemaker implantation (PPMI), 0.778 for broader conduction abnormalities, and 0.747 for paravalvular leak (PVL). No algorithm family showed reproducible superiority (*p* = 0.775). Stronger independent evaluation, calibration/Brier assessment, and decision-curve analysis were reported in only 18/67, 23/67, and 10/67 reports, respectively. **Conclusions:** ML shows promising post-TAVR discrimination, particularly for mortality, but substantial heterogeneity, limited independent validation, and sparse calibration and decision utility assessment constrain clinical translation. A high AUC alone should not be interpreted as evidence of transportability or clinical usefulness.

## 1. Introduction

Severe aortic stenosis is a progressive calcific valve disease for which valve replacement becomes central once symptoms, left ventricular dysfunction, or other high-risk features emerge. Transcatheter aortic valve replacement (TAVR; also termed transcatheter aortic valve implantation [TAVI]) is now established across a broad surgical risk spectrum, and contemporary European Society of Cardiology (ESC)/European Association for Cardio-Thoracic Surgery (EACTS) guidance emphasizes Heart Team assessment that integrates age, life expectancy, anatomy, procedural risk, and lifetime valve strategy [1]. The Society of Thoracic Surgeons (STS)/American College of Cardiology (ACC) Transcatheter Valve Therapy (TVT) Registry recorded 276,316 TAVR procedures in the United States between 2011 and 2019; annual TAVR volume reached 72,991 procedures in 2019, exceeding surgical aortic valve replacement (57,626 procedures). Over the same period, 30-day mortality decreased from 7.2% to 2.5% and 30-day stroke from 2.75% to 2.3%, whereas the need for a new pacemaker remained essentially unchanged (10.9% to 10.8%). Moderate or severe aortic regurgitation at 30 days, including paravalvular leak (PVL), fell from 8.0% to 1.6%; in 2019, 90.3% of patients were discharged home after a median hospital stay of 2 days [2]. These improvements have not eliminated clinically important postprocedural events, making individualized risk prediction relevant to counselling, procedural planning, discharge, and follow-up.

Risk stratification has traditionally relied on surgical scores such as the Society of Thoracic Surgeons Predicted Risk of Mortality (STS-PROM) and the European System for Cardiac Operative Risk Evaluation II (EuroSCORE II) and on TAVR-specific models. The STS/ACC TVT in-hospital mortality model, developed in 13,718 patients and validated in 6868, observed an in-hospital mortality rate of 5.3%; its C-statistic was 0.67 in development and 0.66 in validation, illustrating the modest discrimination of conventional risk tools in this setting [3]. A 2026 systematic review of mortality prediction after TAVR included 36 studies and 272,390 patients, covering six established risk scores and 23 newer machine learning (ML) models, and again showed substantial variation across models and prediction horizons [4]. More broadly, cardiovascular prediction model appraisal has emphasized that algorithmic complexity alone does not ensure valid or transportable performance [5]. Nevertheless, individual ML studies show why the approach remains attractive. In the German Aortic Valve Registry (GARY)/SwissTAVI analysis, 30-day mortality was 3.11% among 28,147 GARY procedures and 3.06% among 6693 external SwissTAVI procedures; the postprocedural TAVI Risk Machine (TRIM) model achieved a C-statistic of 0.79 in the GARY test set and 0.75 in external validation, compared with 0.67 for the STS score in SwissTAVI [6]. In a 54,739-record United States (US) administrative cohort, the five best-performing classifiers each achieved areas under the receiver operating characteristic curve (AUCs) of at least 0.80 for in-hospital mortality [7]. More recently, a publicly trained artificial intelligence (AI)-enabled electrocardiogram (AI-ECG) model externally evaluated in a prospective cohort of 439 TAVR patients achieved AUCs of 0.85 for 30-day mortality and 0.74 for 1-year mortality using a single preprocedural electrocardiogram (ECG) [8].

These findings should not be interpreted as evidence that all ML models address the same clinical problem. A preprocedural model intended for treatment selection or counselling is not interchangeable with a model that incorporates intraprocedural or early postprocedural information; early mortality, longer-term survival, permanent pacemaker implantation (PPMI), and PVL also have different mechanisms, horizons, and decision consequences. Studies often evaluate several algorithms in the same patients, and registries or institutional cohorts can reappear across publications, creating within-study and source-population dependence. In broader clinical prediction research, ML has not demonstrated a consistent performance advantage over logistic regression when comparisons are methodologically sound [9]. Contemporary Transparent Reporting of a multivariable prediction model for Individual Prognosis Or Diagnosis plus Artificial Intelligence (TRIPOD + AI) and Prediction model Risk Of Bias Assessment Tool plus Artificial Intelligence (PROBAST + AI) guidance therefore places particular weight on a clearly defined intended use, separation of model development from evaluation, calibration, external validation, and transparent reporting [10,11].

Recent systematic reviews illustrate both the promise of ML after TAVR and the limitations of the evidence base. Sazzad et al. included 10 studies and 22,933 patients and reported a pooled mortality AUC of 0.79 [12]. Zaka et al. included nine studies and 29,608 patients; the pooled C-statistic was 0.79 for the top-performing ML models versus 0.68 for traditional methods, while only two studies provided external validation and three reported calibration [13]. Jacquemyn et al. reviewed 21 studies, 118,153 patients, and 76 models, with pooled C-statistics of 0.81 for early mortality, 0.76 for 1-year mortality, and 0.75 for PPMI [14]. Sulaiman et al. identified 17 studies and emphasized the predominance of retrospective designs, heterogeneity, and limited generalizability [15]. Shojaei et al. subsequently included 43 studies and 366,269 patients, reporting pooled AUCs of 0.78 for mortality and 0.75 for PPMI or new left bundle branch block (LBBB); only three studies used external validation [16]. These reviews differed in clinical scope and analytic unit, and none jointly addressed source-population overlap, provenance of performance estimates, predictor timing, validation maturity, within-study algorithm dependence, outcome-specific clinical exchangeability, and transportability to new settings.

We therefore conducted an updated, source-audited systematic review and meta-analysis of supervised ML models for clinically relevant outcomes after TAVR. We aimed to quantify outcome-specific out-of-sample discrimination while preserving source family independence; determine how validation design, predictor timing, outcome horizon, data architecture, and methodological quality influence reported performance; compare algorithm and outcome families using dependence-aware methods; and determine whether strong reported discrimination is accompanied by evidence of calibration, transportability, and clinical readiness.

## 2. Methods

### 2.1. Search Strategy

A systematic literature search was conducted in PubMed/MEDLINE, Embase, Scopus, and Web of Science Core Collection from database inception through 6 August 2026, in accordance with the Preferred Reporting Items for Systematic Reviews and Meta-Analyses (PRISMA) 2020 [17]. Database-specific strategies combined controlled vocabulary, where applicable, and free-text terms across three core concepts: TAVR, including the synonymous TAVI terminology, and aortic stenosis; ML and AI (including deep learning, neural networks, random forests, gradient boosting/extreme gradient boosting (XGBoost), support vector machines (SVMs), and ensemble methods); and clinically relevant post-TAVR outcomes, including mortality, PPMI and conduction disturbances, stroke, PVL, readmission, bleeding or vascular complications, and other adverse clinical outcomes. Boolean operators and database-specific syntax were adapted for each source. The complete search strategies for all four databases are provided in Supplementary Table S1.

### 2.2. Eligibility Criteria, Study Selection, and Data Extraction

Studies were eligible if they included adults undergoing TAVR, evaluated a supervised ML or ML-derived multivariable prediction model for a clinically relevant postprocedural outcome, and reported out-of-sample model performance using cross-validation, nested resampling, held-out testing, temporal or geographic validation, inter-centre validation, or independent external validation. Models based on clinical, laboratory, electrocardiographic, echocardiographic, computed tomography (CT), procedural, administrative, longitudinal, or multimodal data were eligible. Outcomes included mortality/survival, PPMI and conduction disturbances, stroke or cerebrovascular events, PVL, heart failure (HF) hospitalization or readmission, bleeding or vascular complications, resource-use outcomes, and other clinically relevant post-TAVR events.

We excluded reviews, editorials, case reports, protocols, conference abstracts without sufficient evaluable data, non-human studies, unsupervised-only analyses, and technical imaging studies limited to segmentation, reconstruction, or procedural planning without a patient-level clinical outcome. Studies combining TAVI and surgical aortic valve replacement were excluded unless TAVR-specific results could be extracted separately. Reports without a usable out-of-sample AUC/C-statistic were excluded from discrimination meta-analysis but could be retained for qualitative or structured synthesis when they provided other relevant model-performance information. Multiple publications derived from overlapping cohorts were not assumed to represent independent evidence.

Study selection, data extraction, and evidence appraisal were conducted in duplicate by paired combinations of three reviewers [S.N.], [D.N.D.], and [E.A.B.]. For each title/abstract, full-text report, and extracted data item, two of these three reviewers performed the assessment; disagreements were discussed with the third reviewer, and any case that remained unresolved was adjudicated by a fourth reviewer [S.F.H.J.]. Title/abstract and full-text screening was managed in Rayyan [18]. Data extraction used a standardized form capturing study setting and design, cohort and evaluation sample size, event frequency, outcome definition and prediction horizon, predictor timing and modality, ML algorithm, validation strategy, missing-data and class imbalance handling, feature selection and model tuning, discrimination and uncertainty estimates, calibration, Brier score, precision–recall performance, decision-curve analysis (DCA), explainability, and external validation. Performance estimates were linked, whenever possible, to the specific model, outcome, prediction horizon, and evaluation dataset from which they were derived [19,20] (Supplementary Tables S2 and S3).

Source family audit. Before quantitative synthesis, every included report was assigned a source family identifier. Reports were linked when explicit statements or a convergence of cohort descriptors supported reuse or overlap of the same patient source, including registry/database or institution(s), recruitment period, eligibility criteria, cohort and event counts, outcome/horizon, and descriptions of derivation or validation datasets; author or institutional overlap was used only as supporting evidence and not as a sole criterion. Same-endpoint reports were not counted as independent when overlap was explicit or strongly supported. If overlap could not be resolved, reports were retained as separate source families but flagged as potential overlap rather than forcibly collapsed. Where a linked source family contributed more than one eligible estimate, one representative effect was retained for the primary outcome synthesis and alternative eligible representatives were examined in sensitivity analyses. Supplementary Table S2 reports the source family identifier and audit rationale for every included report, including linked and potential overlap cases.

### 2.3. Outcome Definitions, Risk of Bias, and Clinical Readiness

The primary quantitative endpoint was out-of-sample discrimination for all-cause mortality, measured by the AUC/C-statistic. Mortality models were additionally classified by prediction horizon (early/in-hospital, approximately 1 year, and >1 year) and by predictor timing (preprocedural vs. peri- or postprocedural or mixed). Electrical outcomes were harmonized conservatively: PPMI was analyzed separately from broader conduction disturbances/LBBB and was not combined with prosthesis–patient mismatch. PVL and stroke/cerebrovascular events were synthesized only when clinically comparable independent estimates were available; otherwise, outcomes were summarized descriptively rather than pooled solely to increase statistical power.

Risk of bias and applicability were assessed using a PROBAST + AI-aligned framework, covering participants/data sources, predictors, outcomes, and analysis [11,21]. These judgments were performed in duplicate by paired combinations of [S.N.], [D.N.D.], and [E.A.B.]; disagreements were discussed with the third reviewer, and unresolved judgments were adjudicated by [S.F.H.J.]. Particular attention was given to predictor timing, effective event numbers, missing-data handling, preprocessing or feature-selection leakage, separation of model tuning from evaluation, and independence of the validation sample. AUC magnitude was not considered when assigning risk-of-bias judgments. Separately, translational maturity was audited through explicit reporting of stronger evaluation, calibration/overall probabilistic accuracy, decision utility, precision–recall performance, and explainability. Stronger external/temporal evaluation was defined as performance assessment in a non-overlapping cohort separated from model development by institution/geography, calendar time, or an explicitly independent dataset; random splitting, bootstrapping, or cross-validation within the development source did not qualify. Calibration referred to agreement between predicted and observed risk (for example, calibration plots, slopes, or intercepts), whereas the Brier score was treated as an overall probabilistic accuracy measure. DCA or net benefit analysis assessed whether using predictions across clinically relevant thresholds could improve decisions compared with default strategies. AUPRC or other precision–recall performance and explicit explainability analyses were recorded separately. These were reporting/readiness signals rather than a composite clinical readiness score. Prospective data collection and multicentre or multisite setting were retained as descriptive report-level characteristics, consistent with TRIPOD + AI principles [10,11] (Supplementary Table S4).

### 2.4. Statistical Analysis

AUCs were synthesized on the logit scale, which provides more appropriate distributional properties for meta-analysis of bounded discrimination measures [22,23,24]:

(1) $$y_{i}=\mathrm{logit}\left(A_{i}\right)=\log\left(\frac{A_{i}}{1-A_{i}}\right)$$

When a 95% confidence interval (CI; $L_{i},U_{i}$) was reported, the corresponding logit-scale standard error was calculated as

(2) $$SE\left(y_{i}\right)=\frac{\mathrm{logit}\left(U_{i}\right)-\mathrm{logit}\left(L_{i}\right)}{2\times 1.96}$$

Author-reported sampling uncertainty was preferred. When no valid CI was available but the numbers of participants with ($n_{1}$) and without ($n_{0}$) the outcome were known, AUC variance was reconstructed using the Hanley–McNeil approximation [25]:

$$Q_{1}=\frac{A_{i}}{2-A_{i}},\;\;Q_{2}=\frac{2A_{i}^{2}}{1+A_{i}},$$

(3) $$\mathrm{Var}\left(A_{i}\right)=\frac{A_{i}\left(1-A_{i}\right)+\left(n_{1}-1\right)\left(Q_{1}-A_{i}^{2}\right)+\left(n_{0}-1\right)\left(Q_{2}-A_{i}^{2}\right)}{n_{1}n_{0}}$$

followed by delta-method propagation:

(4) $$SE\left[\mathrm{logit}\left(A_{i}\right)\right]\approx \frac{SE\left(A_{i}\right)}{A_{i}\left(1-A_{i}\right)}$$

Fold-level standard deviations (SDs), interquartile ranges (IQRs), or variability across repeated cross-validation runs were not interpreted as sampling CIs.

Clinically comparable estimates were pooled using restricted maximum likelihood (REML) random-effects models:

(5) $$y_{i}=\mu +u_{i}+\varepsilon_{i},\;\;u_{i}∼N\left(0,\tau^{2}\right),\;\;\varepsilon_{i}∼N\left(0,v_{i}\right)$$

with inverse-variance weights:

$$w_{i}=\frac{1}{v_{i}+\tau^{2}}.$$

To reduce anticonservative inference in small or heterogeneous meta-analyses, 95% CIs used the modified Hartung–Knapp approach, constraining the residual scale factor to be no smaller than 1 [26,27]:

(6) $$q^{*}=\max\left\{1,\frac{\sum_{i}w_{i}{\left(y_{i}-{\hat{y}}_{i}\right)}^{2}}{k-p}\right\},\;\;SE_{mHK}\left(\hat{\mu }\right)=\sqrt{\frac{q^{*}}{\sum_{i}w_{i}}}$$

Estimates were subsequently back-transformed to the AUC scale. Statistical heterogeneity was described using Cochran Q, I^2^, and τ2 [28]. When sufficient independent evidence was available, a 95% prediction interval was calculated to quantify expected performance in a comparable new setting:

(7) $$\hat{\mu }\pm t_{k-2,\;0.975}\sqrt{{\hat{\tau }}^{\;2}+SE{\left(\hat{\mu }\right)}^{2}}$$

Confidence intervals and prediction intervals answer different questions. The 95% CI quantifies uncertainty around the pooled mean AUC across the evidence base, whereas the 95% prediction interval estimates the range of true AUCs that could plausibly be encountered in a comparable new clinical setting under the random-effects model. Prediction intervals were therefore emphasized alongside pooled CIs because they more directly convey between-setting heterogeneity and transportability uncertainty [22,23,24].

#### Subgroup Analysis and Meta-Regression

Mortality heterogeneity was explored using clinically motivated moderators, including validation maturity, predictor timing, prediction horizon, geographic region, data modality, structured vs. high-dimensional inputs, data source type, publication period, calibration and missing-data reporting, evaluation sample size, event prevalence, precision provenance, and risk of bias.

For moderator $X_{i}$, random-effects meta-regression was specified as

(8) $$y_{i}=\beta_{0}+\beta_{1}X_{i}+u_{i}+\varepsilon_{i}$$

Multilevel categorical moderators were evaluated using indicator terms and omnibus tests where appropriate. Analyses were primarily univariable to limit overfitting and multiplicity; moderator effects were interpreted as study-level associations rather than causal effects.

### 2.5. Dependence-Aware, Bayesian, and Threshold-Level Analyses

#### 2.5.1. Algorithm and Outcome Family Comparisons

Because several algorithms were frequently evaluated within the same cohort, comparative model family analyses did not treat these estimates as independent. Multiple eligible effects were analyzed using multilevel random-effects meta-regression with bias-reduced cluster-robust variance estimation (CR2) and Satterthwaite small-sample degrees of freedom (df) [29,30]. Algorithm families included logistic regression, boosting methods, random forest, SVM, and neural/deep learning approaches. Adjusted marginal AUCs and contrasts were emphasized rather than algorithm rankings; Hotelling’s T^2^-Z (HTZ) small-sample omnibus inference was used where estimable.

A corresponding dependence-aware model compared discrimination across clinically distinct outcome classes while retaining outcome class as a moderator:

(9) $$y_{ij}=\beta_{0}+\sum_{m}\beta_{m}X_{m,ij}+u_{j}+\varepsilon_{ij}$$

where j denotes the source family. This analysis was used to compare outcome-specific discrimination and not to construct a grand pooled AUC across different complications.

#### 2.5.2. Bayesian Analyses

Frequentist synthesis was complemented by a Bayesian normal–normal random-effects model,

(10) $$y_{i}∼N\left(\mu ,v_{i}+\tau^{2}\right)$$

using a weakly informative prior for the pooled logit-AUC and

$$\tau ∼\mathrm{Half}-\mathrm{Normal}\left(0,0.5\right)$$

with a wider scale of 1.0 examined in sensitivity analysis [31]. Posterior predictive distributions were used to characterize discrimination expected in a comparable new setting.

A regularized Bayesian meta-regression further evaluated three prespecified sources of potential performance optimism—evaluation risk of bias, validation maturity, and predictor timing:

(11) $$y_{i}∼N\left(X_{i}\beta ,v_{i}+\tau^{2}\right),\;\;\beta_{j}∼N\left(0,0.5^{2}\right),\;\;\tau ∼\mathrm{Half}\;-\;\mathrm{Normal}\left(0,0.5\right)$$

Posterior effects were summarized using medians, 95% credible intervals (CrIs), posterior probabilities, and adjusted marginal AUCs.

#### 2.5.3. Threshold-Level Performance and Summary Receiver Operating Characteristic (SROC)

Sensitivity, specificity, thresholds, and available 2 × 2 data were independently audited. An operating point was considered eligible only when it corresponded to the same model, outcome, prediction horizon, evaluation cohort, and threshold definition. Individual predictor cut-offs, incomplete operating points, and internal threshold estimates paired with external AUCs were excluded.

Where sufficiently coherent operating points were available, sensitivity and false-positive rate were modelled jointly using a bivariate random-effects model [32]:

(12) $$\begin{array}{c} logit\;({Se}_{i})\\ logit\;(1-{Sp}_{i}) \end{array}\;∼BVN\left(\mu ,\Sigma_{within}+\Sigma_{between}\right)$$

SROC curves, summary sensitivity/specificity, and confidence and prediction regions were derived from this model. Zero cells received a 0.5 continuity correction for variance estimation only. Because threshold-level evidence was sparse, these analyses were considered exploratory.

### 2.6. Sensitivity, Influence, and Heterogeneity Diagnostics

The robustness of the primary mortality synthesis was assessed using leave-one-out analysis, influence and Baujat diagnostics, exclusion of influential high-performance studies, restriction to preprocedural models, alternative source family representatives, alternative mortality horizon selections, reported-CI-only analyses, and progressive inflation of reconstructed standard errors. These prespecified alternatives were also summarized using a specification-curve analysis.

Sensitivity to the heterogeneity estimator and inferential method was examined using REML, maximum likelihood, Paule–Mandel, and DerSimonian–Laird estimators, conventional and modified Hartung–Knapp inference, with a fixed-effect model retained only as a restrictive reference.

A graphical display of study heterogeneity (GOSH) analysis based on random subsets of studies was used to characterize the joint landscape of pooled discrimination and heterogeneity [33]. Radial/Galbraith plots and drapery plots provided complementary assessments of influential studies and confidence geometry [34]. Standard and contour-enhanced funnel plots were considered exploratory; asymmetry was not interpreted as direct evidence of publication bias because prediction model performance can vary with sample size, outcome prevalence, validation design, model selection, and genuine between-setting heterogeneity.

Clinical readiness patterns were summarized descriptively using an UpSet intersection analysis, while sparse or clinically non-exchangeable outcome domains were represented using a harvest/evidence readiness plot rather than statistically inappropriate pooling.

### 2.7. Software, Reproducibility, and Statistical Interpretation

The final numerical workflow was implemented and independently audited in Python (version 3.13.5; Python Software Foundation, Beaverton, OR, USA), using NumPy (version 2.5.3; NumPy Developers, fiscally sponsored by NumFOCUS, Austin, TX, USA), SciPy (version 1.18.1; SciPy Developers, fiscally sponsored by NumFOCUS, Austin, TX, USA), pandas (version 3.0.6; pandas Development Team, fiscally sponsored by NumFOCUS, Austin, TX, USA), and Matplotlib (version 3.11.2; Matplotlib Development Team, fiscally sponsored by NumFOCUS, Austin, TX, USA) for data processing, random-effects estimation, meta-regression, Bayesian computation, bivariate modelling, resampling analyses, and visualization. Equivalent specifications were prepared in R (version 4.4.1; R Foundation for Statistical Computing, Vienna, Austria), principally using metafor for conventional and multilevel meta-analysis and clubSandwich-compatible methods for CR2/Satterthwaite inference [35], providing an independent reproducibility pathway. Complete analysis inputs, uncertainty-provenance tables, code, and vector quality statistical figures were retained in the reproducibility package.

Primary inference centred on pooled mortality discrimination and its uncertainty. Outcome-specific, subgroup, meta-regression, algorithm family, Bayesian, hierarchical summary receiver operating characteristic (HSROC), GOSH, funnel plot, and clinical readiness analyses were interpreted as secondary or exploratory as appropriate. Emphasis was placed on effect magnitude, CIs or CrIs, prediction intervals, small-sample degrees of freedom, and consistency across sensitivity analyses, rather than isolated statistical significance.

### 2.8. Protocol Registration

The review was registered with the International Research Registry (registration number: reviewregistry2130; https://www.researchregistry.com/browse-the-registry/#registryofsystematicreviewsmeta-analyses/registryofsystematicreviewsmeta-analysesdetails/6a84e835fa9190dc53e0929d/) on 18 August 2026, after completion of the database search; therefore, it was not prospectively registered.

### 2.9. Ethical Considerations

Ethics committee review and approval were not required because this systematic review and meta-analysis used only aggregate data from previously published studies and involved no participant recruitment, intervention, or access to identifiable individual-level information. Individual informed consent was therefore not required.

## 3. Results

### 3.1. Study Characteristics, Evidence Structure, and Clinical Readiness

**Figure 1:**
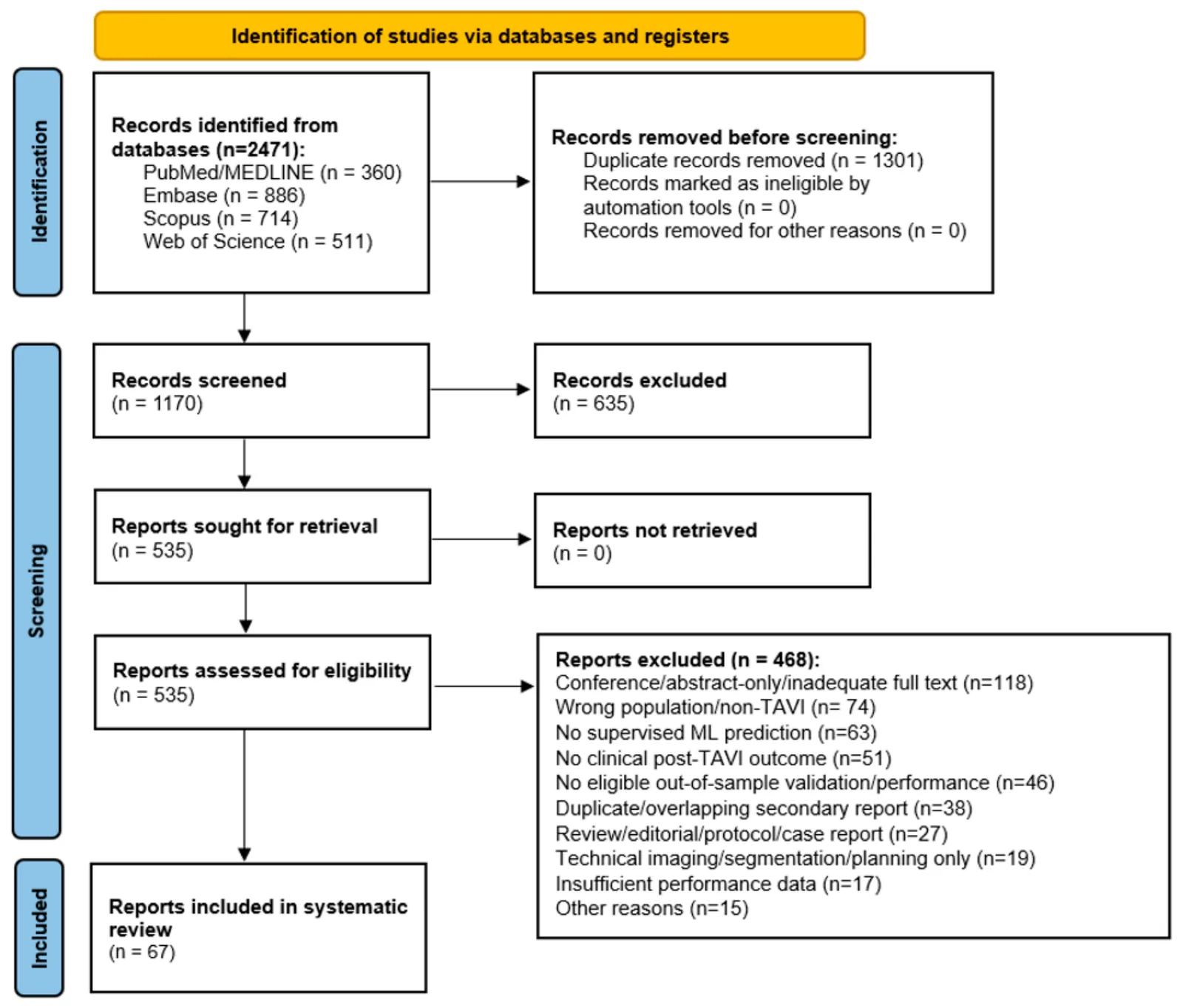
PRISMA 2020 flow diagram of study identification, screening, eligibility assessment, and inclusion.

The final evidence base comprised 67 full-text reports [6,7,8,36,37,38,39,40,41,42,43,44,45,46,47,48,49,50,51,52,53,54,55,56,57,58,59,60,61,62,63,64,65,66,67,68,69,70,71,72,73,74,75,76,77,78,79,80,81,82,83,84,85,86,87,88,89,90,91,92,93,94,95,96,97,98,99], most commonly addressing mortality/survival (27 reports) and electrical or PPMI-related outcomes (15 reports) (Figure 1). The remaining evidence was distributed across composite/patient-centred outcomes (7), valve/structural outcomes (5), multi-outcome studies (4), HF/readmission (3), resource/discharge outcomes (3), bleeding/vascular/technical complications (2), and neurologic outcomes (1). Thus, the available evidence was concentrated in mortality and conduction-related prediction, with substantially less replication for other clinically important post-TAVR outcomes.

**Figure 2:**
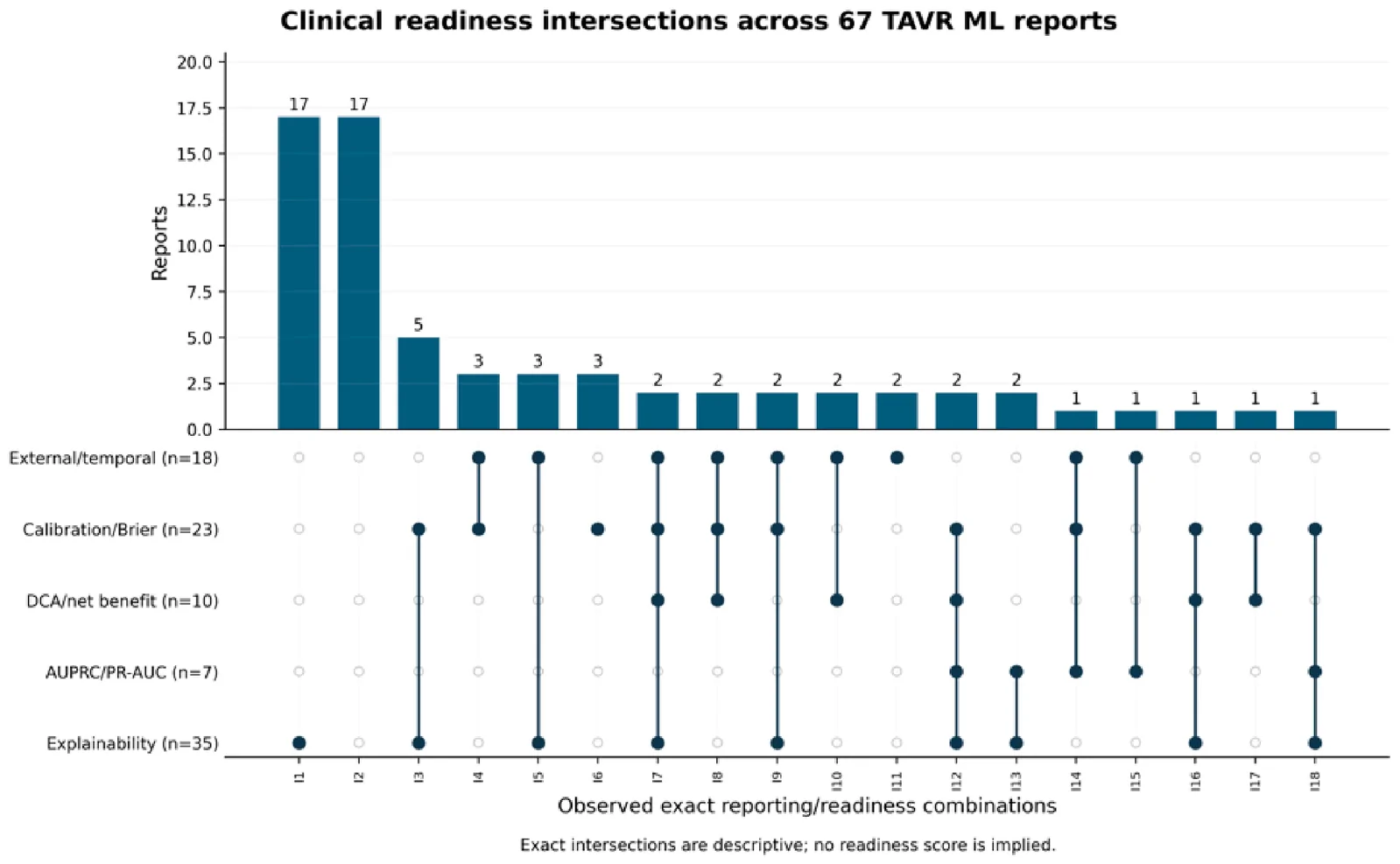
Clinical readiness intersections across the 67-report evidence base. UpSet representation of exact combinations of five strictly audited report-level signals: stronger external or temporal evaluation, calibration or Brier score reporting, DCA or net benefit analysis, AUPRC or precision–recall reporting, and explainability. The figure is descriptive and does not imply a readiness score. Study design/data source descriptors are reported separately in Supplementary Tables S2 and S3. Filled dots indicate signals present within a given intersection, open circles indicate signals absent from that intersection, and vertical connecting lines link signals that co-occur within the same exact combination. Bars above the matrix show the number of reports corresponding to each intersection.

Overall evaluation risk of bias was classified as low in 8/67 reports (11.9%), unclear in 36/67 (53.7%), and high in 23/67 (34.3%); applicability concern was low, unclear, and high in 32, 30, and 5 reports, respectively. Under the strict full-text reporting audit, stronger external, temporal, geographic, or otherwise independent evaluation was identified in 18/67 reports (26.9%), calibration or Brier score assessment in 23/67 (34.3%), DCA or net benefit analysis in 10/67 (14.9%), AUPRC or precision–recall performance in 7/67 (10.4%), and an explicit explainability analysis in 35/67 (52.2%). Study-design/data-source descriptors such as prospective data collection and multicentre or multisite settings were retained descriptively at report level because their terminology was not sufficiently uniform across publications for a single readiness count. Clinical readiness mapping therefore showed substantially greater emphasis on model development and discrimination than on independent validation, calibration, or direct assessment of clinical utility (Table 1; Figure 2; Supplementary Figures S1 and S2).

### 3.2. Primary Meta-Analysis of Mortality Discrimination

**Figure 3:**
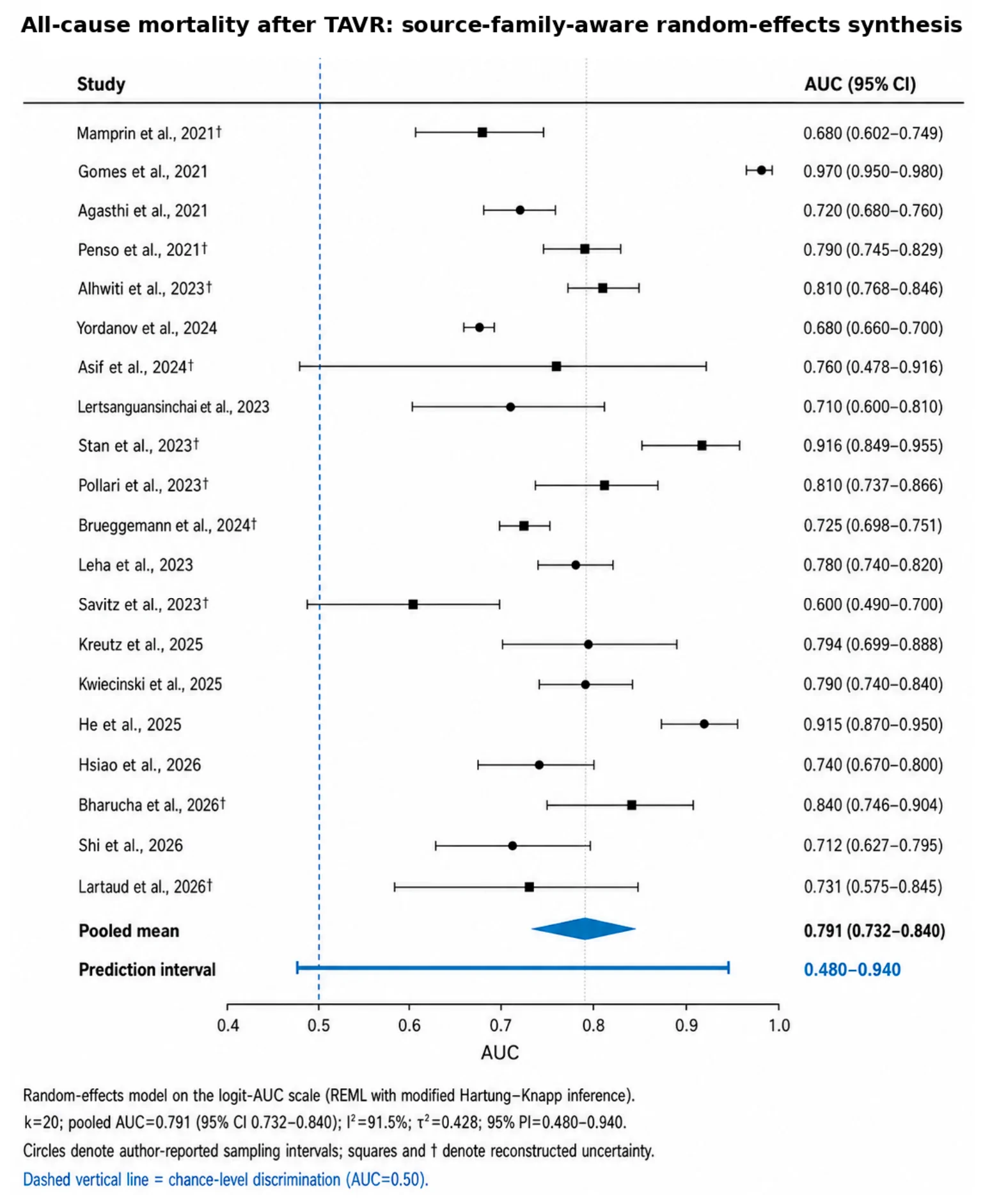
Primary random-effects meta-analysis of out-of-sample mortality discrimination after TAVR. The forest plot shows the 20 independent source family representatives included in the primary mortality synthesis. The pooled AUC was 0.791 (95% CI, 0.732–0.840), with a 95% prediction interval of 0.480–0.940. The CI describes uncertainty in the pooled mean AUC; the prediction interval describes expected variation in the true AUC across comparable new settings. Circles indicate studies with author-reported sampling uncertainty, whereas squares and the † symbol indicate studies for which sampling uncertainty was reconstructed. Horizontal lines represent study specific 95% confidence intervals. The blue diamond represents the pooled mean AUC, and the blue horizontal line represents the 95% prediction interval. The blue dashed vertical line indicates chance level discrimination (AUC = 0.50), whereas the gray dotted vertical line indicates the pooled AUC.

Twenty independent source families contributed to the primary all-cause mortality meta-analysis. The pooled AUC was 0.791 (95% CI, 0.732–0.840). Between-study heterogeneity was substantial (τ^2^ = 0.428; I^2^ = 91.5%; Q = 223.8, *p* < 0.001), and the 95% prediction interval ranged from 0.480 to 0.940 (Figure 3; Supplementary Table S5; Supplementary Figure S3). Given I^2^ = 91.5% and the wide prediction interval, the pooled AUC should be interpreted as an average across heterogeneous populations, outcome horizons, evaluation designs, and modelling contexts rather than as a single transportable performance estimate.

Thus, the mean discrimination was moderate to good, but performance expected in a new comparable population remained highly variable. In practical terms, the relatively narrower CI indicates that the average AUC is estimated with greater precision, whereas the much wider prediction interval indicates that a model evaluated in another clinical setting could perform substantially worse or better than that average.

#### 3.2.1. Validation Maturity

Models evaluated using stronger external, temporal, geographic, or inter-centre designs (k = 8) had a pooled AUC of 0.754 (95% CI, 0.701–0.800) compared with 0.814 (0.716–0.884) for internally evaluated or resampled models (k = 12). The between-group interaction was not statistically significant (*p* = 0.299).

When validation was separated into 3 levels, pooled AUCs were 0.751 (0.666–0.820) for independent/external evaluation (k = 5), 0.758 (0.556–0.887) for temporal/geographic validation (k = 3), and 0.814 (0.716–0.884) for internal holdout or cross-validation (k = 12). Both stronger validation categories therefore produced similar estimates, approximately 0.75, whereas internally evaluated models showed higher average discrimination.

#### 3.2.2. Predictor Timing and Prediction Horizon

Sixteen models restricted to preprocedural predictors yielded a pooled AUC of 0.767 (95% CI, 0.713–0.813), compared with 0.866 (0.520–0.975) among 4 models incorporating peri-procedural, postprocedural, or mixed information (*p* for interaction = 0.083).

**Figure 4:**
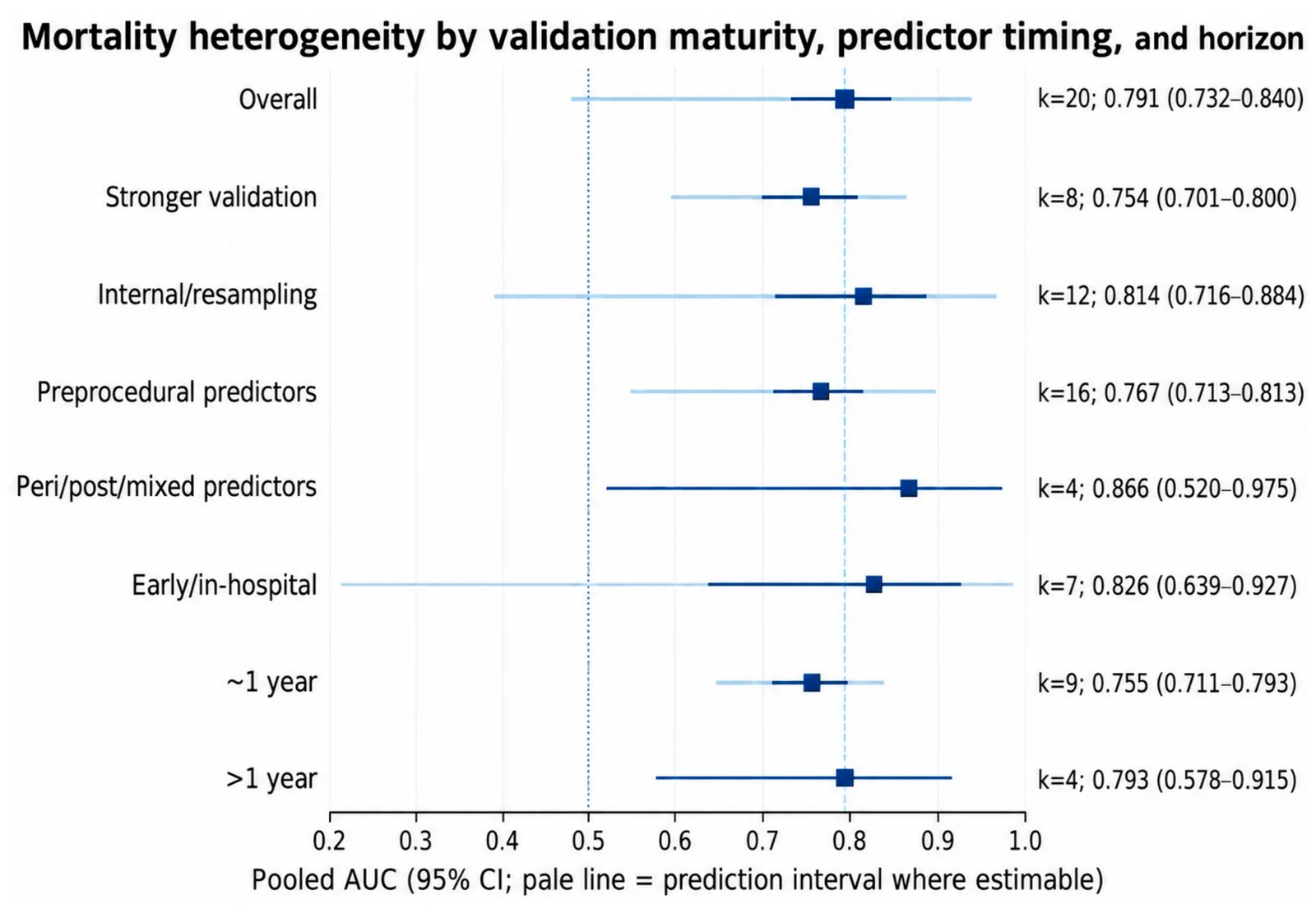
Mortality discrimination according to validation maturity, predictor timing, and prediction horizon. Squares indicate pooled random-effects AUC estimates for the prespecified mortality subgroups, and the darker horizontal lines represent the corresponding 95% confidence intervals. Pale horizontal lines, where estimable, represent 95% prediction intervals. The vertical dashed line at AUC = 0.50 indicates chance-level discrimination. Subgroup contrasts are study level associations and should not be interpreted as causal effects.

By prediction horizon, pooled AUCs were 0.826 (95% CI, 0.639–0.927) for early/in-hospital mortality (k = 7), 0.755 (0.711–0.793) for approximately 1-year mortality (k = 9), and 0.793 (0.578–0.915) for mortality beyond 1 year (k = 4), with no significant overall horizon effect (*p* = 0.550). The 1-year subgroup showed considerably lower heterogeneity (I^2^ = 49.8%) and a prediction interval of 0.646–0.838, representing the most statistically consistent mortality horizon (Figure 4).

### 3.3. Mortality Heterogeneity Across Study and Data Characteristics

Input architecture did not materially explain mortality performance. High-dimensional imaging, signal, or deep multimodal models (k = 7) yielded an AUC of 0.777 (95% CI, 0.682–0.850) compared with 0.798 (0.711–0.865) for structured or engineered models (k = 13; *p* = 0.730). In the more detailed modality classification, AUCs were 0.798 for structured/tabular models, 0.749 for imaging or anatomic models, and 0.807 for ECG or multimodal models (P for omnibus = 0.809) (Supplementary Table S6; Supplementary Figures S4 and S5).

Similarly, no material difference was observed between institutionally curated cohorts and registry/administrative/electronic health record (EHR)-scale datasets (0.796 vs. 0.775; *p* = 0.741).

Studies reporting calibration showed a lower pooled AUC than those not reporting calibration (0.754 vs. 0.814), although the interaction was not statistically significant (*p* = 0.288). Missing-data reporting (*p* = 0.833) and precision provenance (*p* = 0.598) were likewise not associated with mortality discrimination.

Regional estimates were 0.811 (95% CI, 0.726–0.875) for Europe, 0.780 (0.579–0.901) for North America, and 0.725 (0.612–0.816) for Asia; neither regional contrast with Europe was statistically supported.

There was also no evidence of increasing discrimination over time. Models published during 2024–2026 had an AUC of 0.773, compared with 0.806 for earlier studies (*p* = 0.598); continuous publication-year meta-regression was likewise null (β = −0.058 logit-AUC/year; *p* = 0.536). Evaluation sample size (β = −0.093 for log[N]; *p* = 0.449) and event prevalence (β = −0.037; *p* = 0.805) were not associated with AUC.

Collectively, these conventional study and model characteristics explained little of the observed heterogeneity.

### 3.4. Risk of Bias and Apparent Performance Optimism

**Figure 5:**
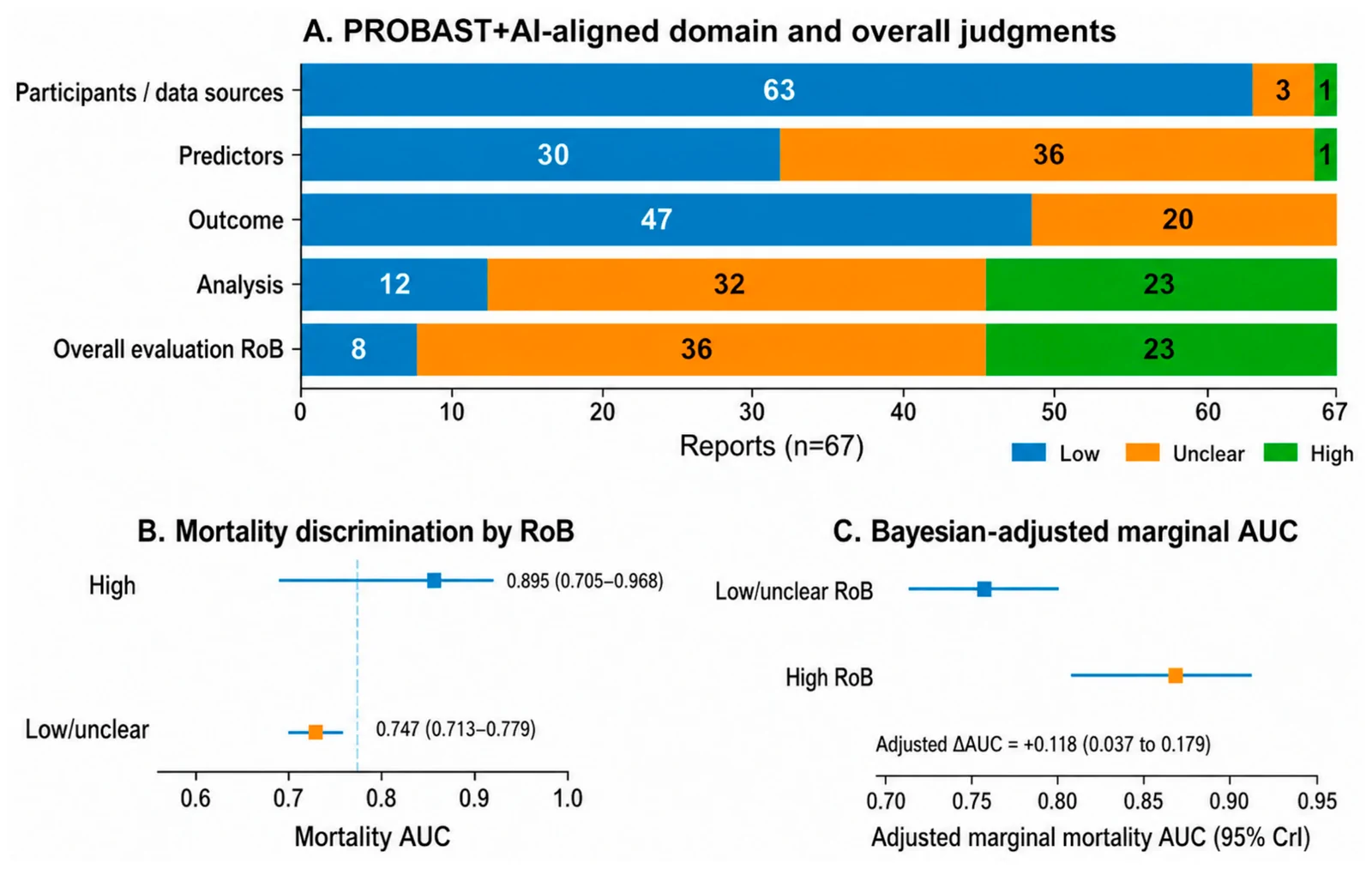
Methodological quality and apparent mortality performance. (**A**) PROBAST + AI-aligned domain and overall risk-of-bias distribution across the 67 reports. (**B**) Mortality discrimination stratified by evaluation risk of bias; the high-risk subgroup was small and the association is observational. (**C**) Bayesian-adjusted marginal mortality AUC for low or unclear versus high evaluation risk of bias after accounting for validation maturity and predictor timing. AUC magnitude was not used to assign risk-of-bias judgments. In panel (**B**), the vertical dashed line indicates the pooled AUC of the low/unclear-risk subgroup, which served as the reference group.

Evaluation risk of bias showed the clearest association with mortality discrimination. Five studies classified as high risk yielded a pooled AUC of 0.895 (95% CI, 0.705–0.968), compared with 0.747 (0.713–0.779) among 15 studies classified as low or unclear risk (*p* for interaction = 0.0009) (Figure 5; Supplementary Figures S6 and S7). Because the high-risk subgroup contained only five studies, this is an observational study-level association and not evidence that risk of bias itself causally increased AUC.

The association remained present in Bayesian meta-regression after simultaneous adjustment for validation maturity and predictor timing. The posterior median coefficient for high risk of bias was +0.833 logit-AUC units (95% CrI, +0.238 to +1.341), with a 99.6% posterior probability of a positive association (Supplementary Figure S8). However, residual confounding by correlated study characteristics (including small evaluation samples, single-centre design, outcome horizon, predictor timing, and model selection practices) cannot be excluded.

Adjusted marginal AUCs were 0.879 (95% CrI, 0.809–0.920) for high-risk studies and 0.759 (0.713–0.804) for low- or unclear-risk studies, corresponding to an adjusted difference of +0.118 AUC units (95% CrI, +0.037 to +0.179). In the same model, stronger validation showed little independent residual association, whereas peri- or postprocedural or mixed predictor timing retained a positive but uncertain effect. As a sensitivity analysis, excluding all five high-risk mortality studies leaves the 15 low/unclear-risk studies with the prespecified subgroup estimate of AUC 0.747 (95% CI, 0.713–0.779); this demonstrates sensitivity of the pooled level to study quality but does not identify the causal mechanism of the difference.

### 3.5. Comparison of ML Algorithm Families

**Figure 6:**
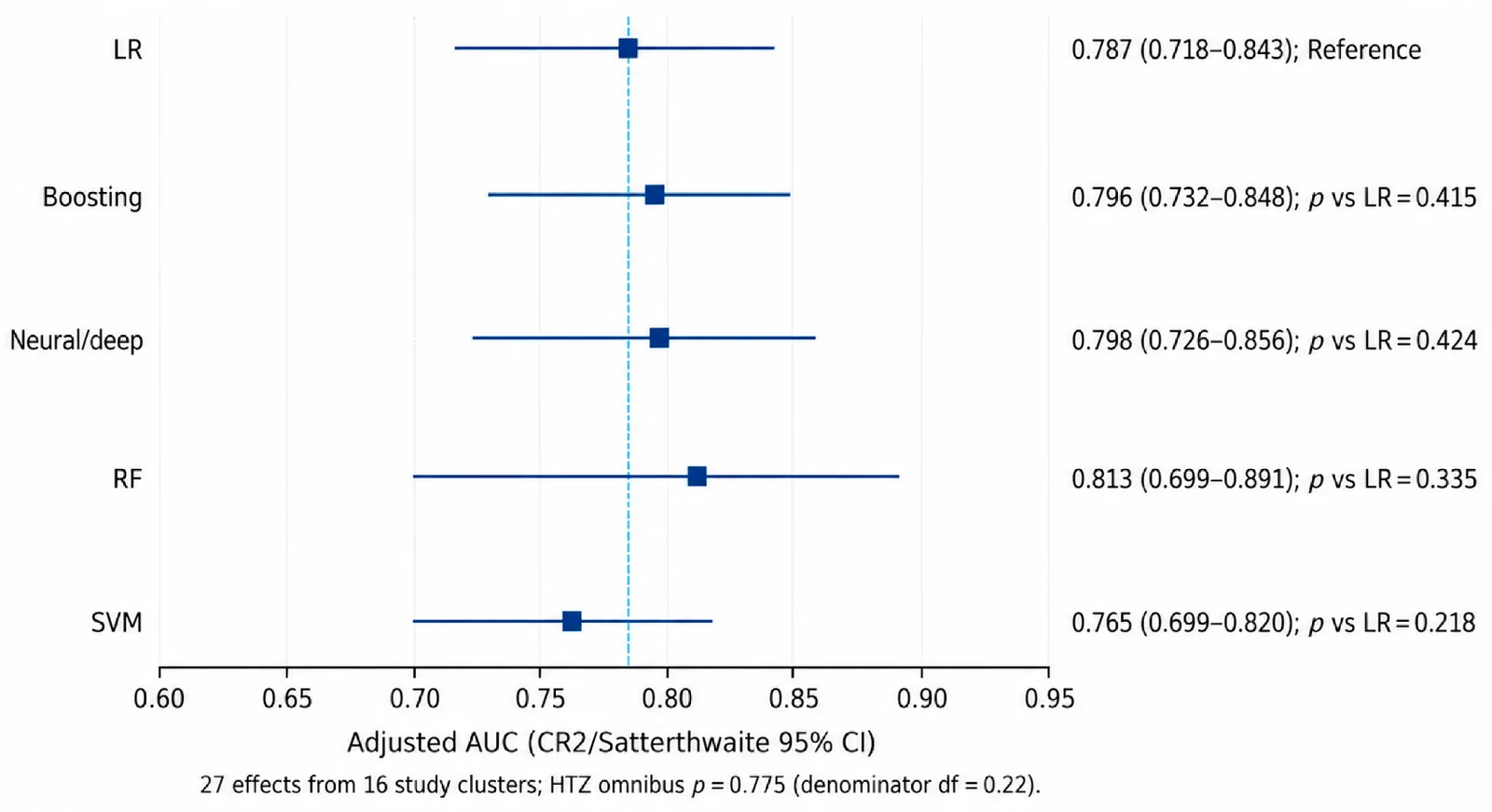
Dependence-aware comparison of mortality discrimination across algorithm families. Reported AUCs are model-based adjusted marginal estimates from multilevel meta-regression with CR2 cluster-robust variance estimation, accounting for multiple algorithms evaluated within the same source family. The available evidence did not establish reproducible superiority of any algorithm family; the estimates should not be interpreted as evidence of algorithm equivalence. The vertical dashed line indicates the adjusted marginal AUC of the logistic regression reference group (AUC = 0.787).

The dependence-aware algorithm analysis included 27 model family estimates from 16 independent study clusters. Adjusted AUCs were as follows (Figure 6; Supplementary Table S7):

- Logistic regression, 0.787 (95% CI, 0.718–0.843);
- Boosting methods, 0.796 (0.732–0.848);
- Neural/deep learning models, 0.798 (0.726–0.856);
- Random forest, 0.813 (0.699–0.891);
- SVM, 0.765 (0.699–0.820).

None of the contrasts with logistic regression were statistically significant (boosting, *p* = 0.415; neural/deep learning, *p* = 0.424; random forest, *p* = 0.335; SVM, *p* = 0.218). The overall HTZ test was also non-significant (*p* = 0.775), but the very small denominator degrees of freedom indicate limited information for a global comparison. Accordingly, the non-significant omnibus test represents absence of evidence for reproducible algorithm family superiority, not evidence that the algorithm families are equivalent.

No algorithm family therefore demonstrated reproducibly superior discrimination after accounting for within-study dependence; the comparison remains imprecise and should be interpreted cautiously.

### 3.6. Outcome-Specific Meta-Analyses Beyond Mortality

#### 3.6.1. PPMI and Conduction Abnormalities

Six independent studies contributed to the strict PPMI synthesis, yielding a pooled AUC of 0.760 (95% CI, 0.583–0.878), with substantial heterogeneity (I^2^ = 92.6%; τ^2^ = 0.554) and a prediction interval of 0.251–0.968. Including an additional matched case–control study produced a similar estimate (AUC, 0.751; 95% CI, 0.609–0.854) (Supplementary Figure S9A).

Broader conduction/LBBB outcomes were analyzed separately (k = 6) and yielded an AUC of 0.778 (95% CI, 0.544–0.912). Exclusion of one extreme internally evaluated estimate reduced the pooled AUC to 0.730 (0.608–0.825). The exploratory comparison between strict PPMI and broader conduction outcomes was not significant (*p* = 0.824).

#### 3.6.2. PVL and Cerebrovascular Outcomes

Three independent studies of significant/residual PVL yielded a pooled AUC of 0.747 (95% CI, 0.358–0.940; I^2^ = 73.2%). Three studies evaluating stroke/cerebrovascular events yielded an exploratory pooled AUC of 0.710 (95% CI, 0.323–0.926) (Supplementary Figure S9B).

#### 3.6.3. Dependence-Aware Comparison Across Outcomes

**Figure 7:**
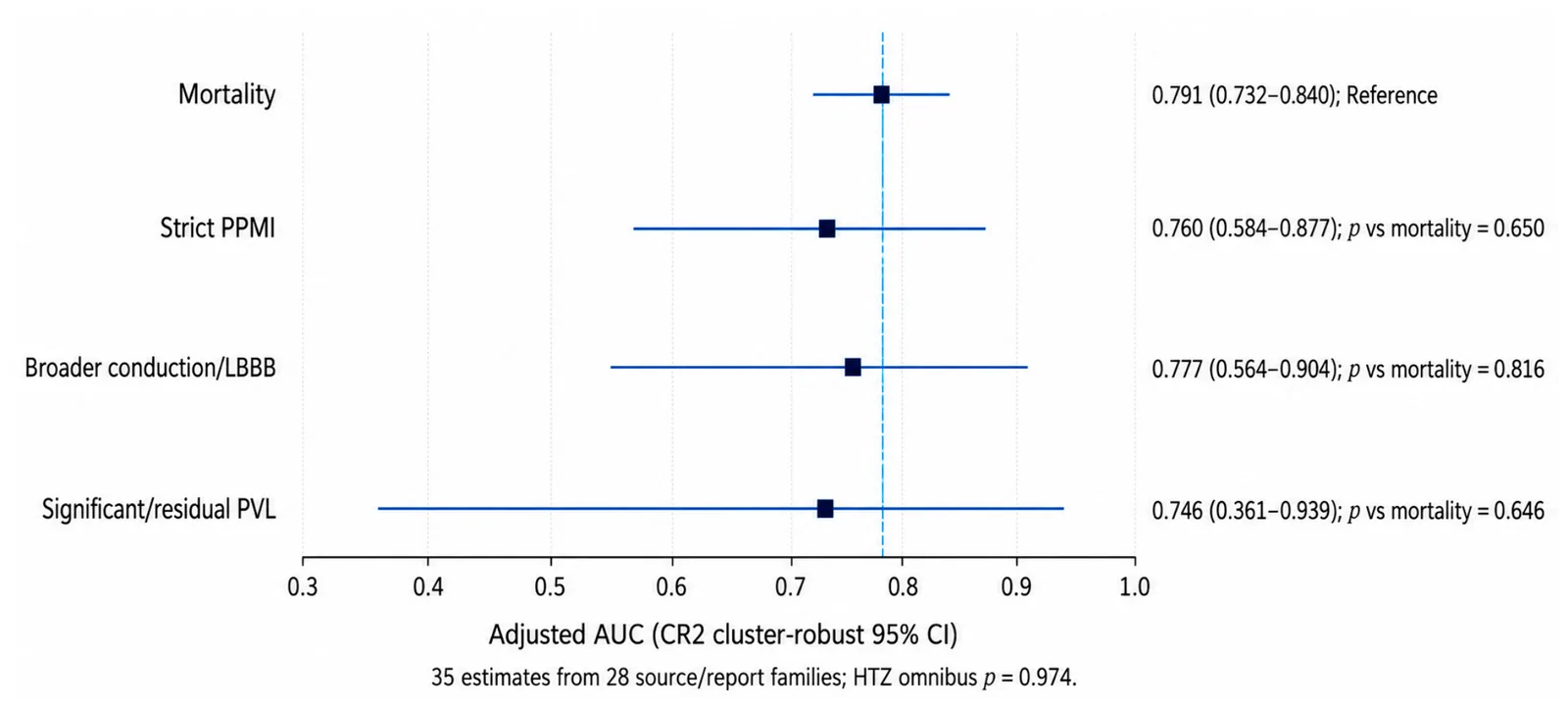
Dependence-aware comparison across clinical outcome classes. Reported AUCs are model-based adjusted marginal estimates from the cluster-robust multilevel meta-regression for mortality, strict PPMI, broader conduction/LBBB, and PVL. Squares indicate adjusted marginal AUC estimates and horizontal lines indicate the corresponding 95% confidence intervals. The vertical dashed line indicates the adjusted marginal AUC of the mortality reference group (AUC = 0.791), against which the other outcome-class contrasts were evaluated. The analysis compares outcome-specific discrimination and does not constitute a grand pooled endpoint.

The multilevel outcome-class analysis included 35 estimates from 28 independent source families. Adjusted AUCs were 0.791 for mortality, 0.760 for strict PPMI, 0.777 for broader conduction/LBBB, and 0.746 for PVL. None differed significantly from mortality, and the omnibus comparison was non-significant (*p* = 0.974; denominator df = 3.59) (Figure 7).

The point estimates were of a broadly similar order across the four outcome classes, but the non-significant omnibus comparison should not be interpreted as equivalence because precision, denominator degrees of freedom, and evidence maturity differed substantially.

### 3.7. Threshold-Level and Bayesian Assessment of Transportability

Four independent studies provided sufficiently comparable model-level operating points for exploratory bivariate analysis of early mortality [6,7,8,36]. The summary sensitivity was 0.818, specificity 0.795, and SROC AUC 0.855. When analysis was restricted to baseline/preprocedural model variants, sensitivity decreased to 0.620, specificity remained 0.808, and SROC AUC decreased to 0.792. Given the small number of studies and the combination of in-hospital and 30-day mortality, these estimates were considered exploratory (Supplementary Table S8; Supplementary Figure S10A,B).

**Figure 8:**
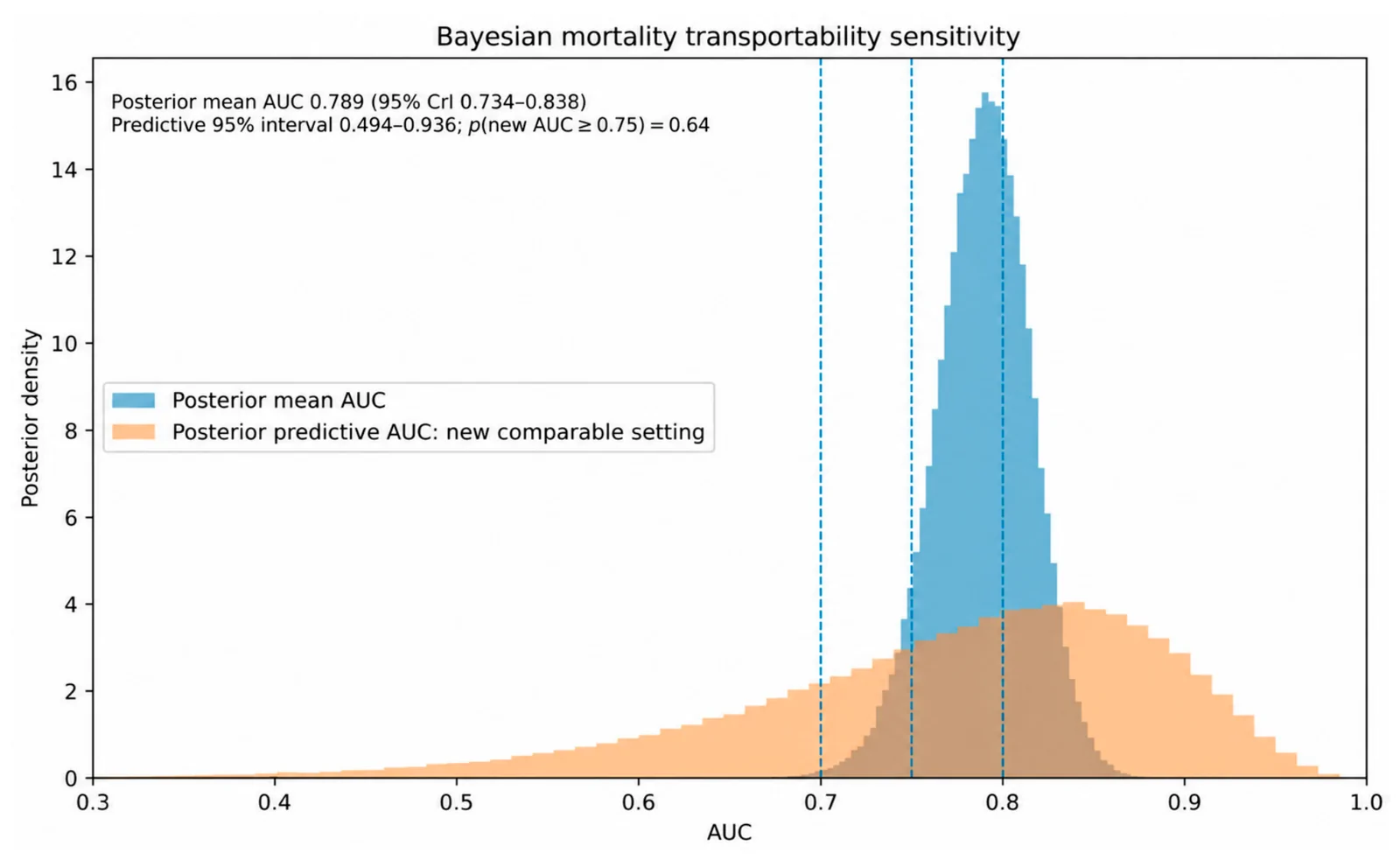
Bayesian assessment of mortality transportability. The posterior distribution for the mean mortality AUC is contrasted with the posterior predictive distribution for the AUC in a comparable new setting; the broader predictive distribution quantifies between-setting uncertainty. Vertical dashed lines indicate key posterior summary values, including the posterior mean mortality AUC, the AUC threshold of 0.75 used for the probability statement *p* (new AUC ≥ 0.75), and the relevant interval markers shown in the figure.

The Bayesian random-effects mortality analysis closely reproduced the frequentist estimate, with a posterior median mean AUC of 0.790 (95% CrI, 0.734–0.838). However, predicted performance in a new comparable setting remained uncertain: the posterior predictive median AUC was 0.791, with a 95% predictive CrI of 0.494–0.936 (Figure 8).

The posterior probabilities of achieving an AUC of at least 0.70, 0.75, and 0.80 in a new setting were 0.767, 0.637, and 0.465, respectively.

Only four model–outcome pairs provided directly traceable development/internal and external AUCs. External discrimination was lower in all four, with absolute changes ranging from −0.010 to −0.222 and a median change of −0.094 (Table 2). Because the pairs represented different outcomes, prediction horizons, and validation transitions and within-pair covariance was unavailable, the differences were summarized descriptively and were not meta-analyzed. This four-pair analysis is illustrative and hypothesis-generating only; it does not estimate an average external-validation decrement for TAVR ML models.

### 3.8. Robustness and Influence Analyses

The primary mortality result was stable across extensive sensitivity analyses. Leave-one-out pooled AUCs ranged from 0.769 to 0.799, and no single study reversed the overall conclusion. Exclusion of the most influential high-performing study reduced the pooled AUC to approximately 0.769, while simultaneous exclusion of two influential high-performing studies produced an AUC of 0.757 (95% CI, 0.718–0.791) (Supplementary Figures S11 and S12). In the reviewer-requested risk-of-bias restriction, exclusion of all five high-risk mortality studies left the 15 low/unclear-risk studies with the prespecified subgroup estimate of 0.747 (95% CI, 0.713–0.779) (Figure 5; Supplementary Table S9; Supplementary Figures S6–S8). This restriction is the exact complement of the five-study high-risk subgroup and is therefore reported as a sensitivity expression of the prespecified subgroup analysis rather than as a newly parameterized heterogeneity model. These analyses show that the overall mean signal persists, while its magnitude is lower when evidence with greater evaluation bias concerns is removed.

Across 16 predefined analytic specifications—including alternative source family representatives, restriction to preprocedural models, alternative outcome horizon selection, reported-CI-only analyses, influential-study exclusions, and progressive inflation of reconstructed standard errors—the pooled mortality AUC remained within 0.757–0.805 (Supplementary Table S9; Supplementary Figure S13).

Results were similarly stable across random-effects estimators: REML, maximum likelihood, Paule–Mandel, and DerSimonian–Laird models all produced pooled AUCs between 0.789 and 0.791. The substantially lower fixed-effect estimate (0.736; 95% CI, 0.725–0.746) occurred in the presence of pronounced heterogeneity and was therefore not considered an appropriate summary model (Supplementary Figure S14).

GOSH analysis of 25,000 random subsets produced a median pooled AUC of approximately 0.790, with the central 95% of subset estimates ranging from approximately 0.730 to 0.848. Study composition had considerably greater influence on estimated heterogeneity than on the pooled mean (Supplementary Figure S15).

Exploratory funnel and contour-enhanced funnel plots showed some asymmetry, but the continuous standard-error moderator was not significant (*p* = 0.279). Given the substantial variation in validation strategy, populations, event prevalence, and model-selection procedures, funnel asymmetry was not interpreted as direct evidence of publication bias (Supplementary Figure S16).

### 3.9. Overall Evidence Pattern

Across the quantitative analyses, mortality discrimination remained consistently centred around AUC 0.79, with little sensitivity to estimator choice, representative-study selection, or other defensible analytical decisions. In contrast, prediction intervals and Bayesian predictive distributions demonstrated substantial uncertainty regarding performance in new populations.

No reproducible superiority was established for a particular ML family or for high-dimensional/multimodal data over structured clinical data. The largest observed performance contrast was associated with evaluation risk of bias, but this finding was based on only five high-risk mortality studies and remains an observational association susceptible to confounding by correlated design characteristics.

At the evidence-base level, independent validation, calibration, DCA, and precision–recall evaluation remained much less common than model development or internal performance assessment. Thus, the principal limitation of the current literature was not the absence of models with high reported AUCs, but the limited evidence that such performance is reproducible, calibrated, and clinically transportable.

## 4. Discussion

### 4.1. Principal Findings

This source-audited synthesis of 67 reports found a stable average mortality signal but substantial uncertainty in transportability. The pooled mortality AUC was 0.791 (95% CI, 0.732–0.840), yet heterogeneity was high (I^2^ = 91.5%) and the 95% prediction interval spanned 0.480–0.940. Stronger validation and genuinely preprocedural prediction produced lower point estimates than internal evaluation or models using peri-/postprocedural information. High-risk studies also reported higher apparent AUCs, but that comparison is observational and based on only five high-risk mortality studies. Dependence-aware analyses did not establish superiority of any algorithm family. Accordingly, the pooled AUC is best interpreted as an average signal across heterogeneous evidence rather than as a clinically portable performance guarantee.

### 4.2. Mortality Prediction: A Reproducible Signal, but Not a Reproducible Guarantee

Earlier TAVR ML reviews generally reported mortality discrimination in a similar broad range, but their estimands differed because of eligibility criteria, outcome horizons, validation design, model selection, and handling of repeated cohorts [4,12,13,14,15,16] (Table 3). The present review therefore should not be interpreted as a head-to-head or temporal comparison with those meta-analyses; its main distinction is source family auditing, dependence-aware inference, explicit predictor–time alignment, and direct assessment of transportability and clinical readiness signals.

Source family auditing materially changes the unit of inference: repeated or overlapping patient sources are not automatically treated as independent evidence. One representative effect per linked source family was retained for the primary mortality synthesis, while alternative representatives were tested in sensitivity analyses and multiple within family algorithm estimates were handled using cluster-robust models. This approach reduces the risk of precision inflation from duplicated patient populations.

The distinction between the CI and prediction interval is clinically important. The CI quantifies uncertainty in the pooled mean AUC, whereas the prediction interval reflects the range of true performance that may occur in a comparable new setting. Here, the prediction interval ranged from near-chance to excellent discrimination, and the Bayesian analysis placed the probability of a new-setting AUC ≥ 0.80 below 50%. Thus, the apparently precise average AUC coexists with substantial uncertainty for deployment in another centre or population [22,23,24].

Only 18/67 reports met the stricter stronger evaluation definition, and mortality models evaluated with external, temporal, geographic, or inter-centre designs had a lower point estimate than internally evaluated models, although the subgroup interaction was not statistically significant. Differences in case mix, outcome incidence, measurements, treatment patterns, and device era can alter discrimination and calibration across settings [5,100]. The four traceable internal–external pairs all showed lower external AUCs, but these pairs were heterogeneous and too few for generalization; they are descriptive examples, not an estimate of the expected external-validation loss.

### 4.3. Prediction Time and Methodological Quality Shape Apparent Performance

Predictor timing is part of the clinical question. Peri- or postprocedural haemodynamics, new conduction abnormalities, procedural complications, and early physiological responses may be legitimate predictors for postprocedural triage, but they are not available to a pre-TAVR model intended for Heart Team counselling or treatment planning. Models with different index times should therefore not be compared as if they addressed the same decision [1,5,101].

Risk of bias was a major interpretive concern: 59/67 reports were classified as high or unclear overall evaluation risk of bias, and only five high-risk mortality studies drove the high-risk subgroup estimate. Although the adjusted AUC difference was approximately 0.12, this study-level association cannot be interpreted causally. Small evaluation samples, single-centre design, outcome horizon, predictor timing, leakage, and model-selection practices may be correlated with risk-of-bias classification and apparent performance. Excluding the five high-risk mortality studies reduced the pooled estimate to 0.747 (95% CI, 0.713–0.779), reinforcing the need to interpret very high AUCs in light of evaluation design and performance provenance rather than as proof of superior prediction [9,11].

### 4.4. Algorithmic Complexity Versus Clinically Meaningful Signal

After accounting for within-study dependence, adjusted marginal mortality AUCs ranged from 0.765 to 0.813 across the five algorithm families. None of the contrasts with logistic regression was statistically significant, but the small denominator degrees of freedom limit power. The non-significant omnibus test is therefore an absence-of-evidence result, not evidence of algorithm equivalence. Selecting the numerically highest AUC from many algorithms tested in the same cohort can exaggerate apparent differences.

High-dimensional imaging, signal, and multimodal inputs likewise did not show reproducibly higher mortality discrimination than structured data. This does not imply that richer data sources lack value; rather, their incremental contribution should be demonstrated against strong simpler baselines using rigorous independent evaluation. The externally evaluated AI-ECG study by Hsiao et al. illustrates this principle [8].

### 4.5. Non-Mortality Outcomes: Prediction Must Remain Clinically Task-Specific

Electrical complications were the most developed non-mortality domain, but strict PPMI still showed substantial heterogeneity and a wide prediction interval. Broader conduction outcomes were kept separate because permanent pacing, bundle-branch block, transient atrioventricular block, and composite conduction endpoints have different mechanisms and clinical consequences. PVL and cerebrovascular evidence was sparser, with only three independent effects in each pooled analysis; consistent endpoint definitions and timing, including VARC-3 conventions, would improve comparability [102,103].

Threshold-level and clinical utility evidence was sparse. AUC measures ranking ability over thresholds but does not establish calibration or whether acting on predictions improves patient-level decisions. Calibration evaluates agreement between predicted and observed risk, while DCA evaluates net benefit across clinically relevant thresholds. Consequently, a high AUC without adequate calibration and decision utility assessment is insufficient evidence for clinical use [104].

### 4.6. From Discrimination to Clinical Readiness

The readiness audit showed a clear implementation gap: only 18/67 reports had stronger independent/temporal/geographic/inter-centre evaluation, 23/67 reported calibration or a Brier score, and 10/67 reported DCA or net benefit. These metrics answer different questions: discrimination ranks patients, calibration assesses the accuracy of predicted risk, and DCA assesses whether threshold-based use could improve decisions. Their sparse reporting means that strong AUCs alone cannot establish generalizability or clinical readiness [10,104,105].

By the revised ESC evidence framework, a split sample from the development source is not independent external validation, and high-level evidence requires successful evaluation in multiple independent datasets [106]. Most current TAVR ML evidence therefore remains preliminary for implementation. Future work should evaluate models across centres, calendar periods, device platforms, and clinically relevant subgroups, with recalibration or updating when performance shifts.

These translational concerns are not unique to TAVR: systematic reviews and meta-analyses of ML after coronary interventions have also reported substantial between-study heterogeneity for some algorithm families and emphasized the need for further or prospective external validation before clinical translation [107,108]. Future studies should prespecify intended use, index time, outcome definition, and prediction horizon; keep preprocessing, feature selection, and tuning within resampling; reserve independent data for evaluation; and report discrimination, calibration, overall probabilistic accuracy, clinically actionable thresholds, and net benefit. Prospective impact studies are ultimately required to determine whether model-informed care changes management or patient outcomes [100,101,109].

### 4.7. Strengths and Limitations

This review has several strengths. The search was updated through August 2026; repeated patient sources were audited at the source family level; performance estimates were linked to specific models, outcomes, horizons, and evaluation datasets; non-exchangeable outcomes were not pooled merely to increase power; and within-study dependence was addressed explicitly. The mortality result was examined using prediction intervals, Bayesian predictive modelling, alternative heterogeneity estimators, leave-one-out analyses, source family swaps, uncertainty inflation, and 16 analytic specifications. Across those specifications the pooled mortality AUC remained 0.757–0.805, indicating that the estimated mean was more stable than the between-setting heterogeneity.

Several limitations remain. First, heterogeneity was substantial and clinically meaningful: studies differed in dataset size, event frequency, outcome definitions, follow-up horizons, predictor timing, data modality, model family, and validation design. These differences change case mix, effective information, and the estimand being evaluated, and study-level meta-regression cannot fully explain or causally attribute their effects. Second, 59/67 reports were at high or unclear evaluation risk of bias, limiting confidence in the apparent performance of the evidence base; the high-risk mortality subgroup contained only five studies and its higher AUC may reflect correlated features such as small samples or single-centre design. Third, independent/temporal evaluation, calibration, DCA, and threshold-level evidence were sparse, so generalizability and clinical usefulness remain incompletely established even when discrimination is high. Fourth, some source family relationships could only be classified as probable or potential, and reconstructed AUC uncertainty is less secure than author-reported sampling uncertainty. Finally, non-mortality and algorithm family comparisons contained limited independent information, and aggregate data precluded direct assessment of calibration transportability, subgroup fairness, model updating, or patient-level net benefit.

## 5. Conclusions

ML models for clinical outcomes after TAVR show a reproducible average discrimination signal for mortality, but the pooled AUC should not be interpreted as a transportable or clinically actionable performance estimate. The mean mortality AUC was approximately 0.79, yet I^2^ was 91.5% and the prediction interval was wide, indicating substantial uncertainty across new settings. Stronger validation and restriction to genuinely preprocedural predictors produced lower point estimates, while high-risk studies reported higher apparent performance in an observational comparison based on only five high-risk mortality studies. After accounting for dependence, the available evidence did not establish reproducible superiority or equivalence among algorithm families. Prediction of PPMI is promising but heterogeneous, whereas evidence for PVL, stroke, and other complications remains limited. Clinical translation requires independent and prospective validation, calibration, overall probabilistic accuracy, clinically actionable thresholds, DCA/net benefit, transparent reporting, and model updating across centres and treatment eras.

## Figures and Tables

**Table 1:** Outcome-specific evidence architecture and clinical readiness profile across 67-report corpus.

Outcome Domain	Reports, *n*	Stronger External/ Temporal	Calibration/ Brier	DCA/AUPRC	Explainability	Key Evidence Limitation
Mortality/survival	27	8 (29.6%)	10 (37.0%)	2 (7.4%)/4 (14.8%)	12 (44.4%)	Broad quantitative synthesis feasible; substantial heterogeneity remained.
Electrical/PPMI	15	1 (6.7%)	4 (26.7%)	1 (6.7%)/2 (13.3%)	6 (40.0%)	PPMI and broader conduction outcomes were non-exchangeable.
Composite/patient-centred	7	3 (42.9%)	3 (42.9%)	3 (42.9%)/1 (14.3%)	5 (71.4%)	Heterogeneous composite definitions limited pooling.
Valve/structural	5	2 (40.0%)	2 (40.0%)	2 (40.0%)/0 (0.0%)	4 (80.0%)	PVL remained exploratory; other targets were sparse.
Multi-outcome	4	0 (0.0%)	1 (25.0%)	0 (0.0%)/0 (0.0%)	3 (75.0%)	Multiple non-exchangeable endpoints per report.
HF/readmission	3	2 (66.7%)	1 (33.3%)	1 (33.3%)/0 (0.0%)	1 (33.3%)	Sparse outcome-specific replication.
Resource/discharge	3	1 (33.3%)	1 (33.3%)	0 (0.0%)/0 (0.0%)	3 (100.0%)	Sparse, workflow-specific targets.
Bleeding/vascular/technical	2	1 (50.0%)	1 (50.0%)	1 (50.0%)/0 (0.0%)	1 (50.0%)	Too few independent effects for pooling.
Neurologic	1	0 (0.0%)	0 (0.0%)	0 (0.0%)/0 (0.0%)	0 (0.0%)	Primary-domain evidence was sparse.

Values are report-level counts and percentages within each primary outcome domain. DCA, decision-curve analysis/net benefit; AUPRC, area under the precision–recall curve. Reporting/readiness signals are non-exclusive and are not themselves risk-of-bias judgments. Prospective data collection and multicentre or multisite descriptors are retained in Supplementary Tables S2 and S3 and are not collapsed into a single readiness count.

**Table 2:** Observed generalization gaps in model–outcome pairs with both internal/development and external performance.

Model/Outcome	Development or Internal AUC	External AUC (95% CI)	Delta AUC (External–Internal)	Validation Transition
PREDICT-TAVR bleeding 30-day bleeding	0.790	0.780 (0.720–0.820)	−0.010	bootstrap-corrected → external
major adverse cardiac and cerebrovascular events (MACCE) model 30-day MACCE	0.978	0.756 (0.631–0.881)	−0.222	training → independent validation
HF hospitalization 12-month HF hospitalization	0.957	0.824 (0.693–0.956)	−0.133	derivation → external
AI-ECG mortality 3-year mortality	0.767	0.712 (0.627–0.795)	−0.055	internal → external

The median observed external-minus-internal AUC difference across the four locked pairs was −0.094. These paired differences are descriptive only, were not pooled because outcomes and validation designs differed and covariance was unavailable, and should not be generalized to other outcomes, algorithms, or clinical settings.

**Table 3:** Comparison with recent systematic reviews and meta-analyses of ML prediction after TAVR.

Study	Evidence Base	Clinical Scope/Key Performance	Validation/Quality Focus	Methodological Distinction
Sazzad et al. [12], 2024	4 databases; through 2023; 10 studies/22,933 patients	All-cause mortality; pooled AI AUC ≈ 0.79	Mortality-focused; no source family/dependence model	Current review audits source overlap and separates prediction time, validation maturity, and transportability.
Zaka et al. [13], 2025	4 databases; through Dec 2023; 9 studies/29,608 patients	30-day/1-year mortality; top-performing ML C-statistic 0.79 vs. traditional 0.68	PROBAST; 2 externally validated studies; calibration uncommon	Current review avoids winner selection and uses CR2 dependence-aware model family inference.
Jacquemyn et al. [14], 2025	3 databases; through Aug 2023; 21 studies/118,153 patients; 76 models	Early mortality ≈ 0.81; 1-year mortality ≈ 0.76; PPMI ≈ 0.75	PRISMA/CHARMS/TRIPOD; PROBAST; model transparency	Adds source overlap audit, performance provenance, predictor timing, Bayesian prediction, and CR2-RVE.
Sulaiman et al. [15], 2025	PubMed + Scopus; 17 studies	Broad TAVI outcomes; narrative synthesis without pooled performance	Retrospective designs, heterogeneity, limited generalizability	Current review adds outcome-specific quantitative synthesis while keeping non-exchangeable outcomes separate.
Shojaei et al. [16], 2025	Broad search; through 2024; 43 studies/366,269 patients	Mortality AUC 0.78; PPI/LBBB AUC 0.75	40 internal vs. 3 external validation studies; high heterogeneity	Current review avoids best-AUC selection, audits repeated cohorts, and evaluates calibration/utility/readiness.
Wang et al. [4], 2026	4 databases; through Jun 2024; 36 studies/272,390 patients	Mortality; established scores + 23 ML models; summarized ML C-index 0.705	PROBAST; time-point subgrouping; mixed score/ML estimands	Current mortality estimand is source-audited out-of-sample ML discrimination with prediction intervals and Bayesian transportability.
Present review, 2026	4 databases; through Aug 6, 2026; 67 full-text reports	Mortality + electrical, PVL, cerebrovascular, and structured other outcomes; mortality AUC 0.791	PROBAST + AI-aligned appraisal; calibration/utility/readiness audit	One representative effect per source family; CR2-RVE; predictor–time alignment; mHK prediction intervals; Bayesian predictive analysis.

CHARMS, CHecklist for critical Appraisal and data extraction for systematic Reviews of prediction Modelling Studies; RVE, robust variance estimation; mHK, modified Hartung–Knapp. Values are shown only to clarify differences in review estimands; they are not head-to-head comparisons. Eligibility criteria, outcome horizons, model selection, validation design, and handling of repeated cohorts can materially change the summarized performance.

## Data Availability

The datasets analyzed during the current study are available upon request with no restrictions.

## Supplementary Materials

The following supporting information can be downloaded at: https://www.mdpi.com/article/10.3390/jcm15197692/s1. Figure S1: Strictly audited clinical readiness milestones across the 67-report corpus; Figure S2: Report-level matrix of five strictly audited clinical readiness/reporting signals; Figure S3: Drapery plot for the primary mortality synthesis; Figure S4: Mortality subgroup estimates by data modality and source type; Figure S5: Evaluation-sample-size and event-prevalence meta-regression diagnostics; Figure S6: Study-level PROBAST+AI-aligned evaluation risk-of-bias traffic-light assessment across the 67 included reports; Figure S7: Source-audited mortality design-quality multiverse; Figure S8: Regularized Bayesian meta-regression coefficients for high evaluation risk of bias, stronger validation, and predictor timing; Figure S9: Outcome-specific forest plots: strict permanent pacemaker implantation and exploratory significant/residual paravalvular leak; Figure S10: Exploratory early-mortality HSROC analyses: representative operating points and preprocedural-only sensitivity analysis; Figure S11: Leave-one-out robustness of the primary mortality meta-analysis; Figure S12: Influence diagnostic for the primary mortality synthesis; Figure S13: Selected mortality sensitivity and specification analyses; Figure S14: Sensitivity of the primary mortality estimate to heterogeneity estimator and inferential method; Figure S15: GOSH landscape based on 25,000 random subsets of the final mortality evidence set; Figure S16: Contour-enhanced mortality funnel plot; Table S1: Database-specific search strategies for the final 6 August 2026 update; Table S2: Full-text source-audited study, outcome, model, and evaluation characteristics of the 67 included reports; Table S3: Full-text methodological, performance-reporting, and clinical readiness extraction across the 67 included reports; Table S4: PROBAST+AI-aligned risk-of-bias and applicability assessment; Table S5: Primary mortality meta-analysis inputs and performance provenance; Table S6: Mortality subgroup and meta-regression results; Table S7: Dependence-aware algorithm-family meta-regression; Table S8: Audited early-mortality threshold-level operating points; Table S9: Mortality specification and sensitivity analyses. File S1: Completed PRISMA 2020 checklist.
