# Supplementary material for: Machine Learning for Clinical Outcome Prediction After Transcatheter Aortic Valve Replacement: A Systematic Review and Meta-Analysis of Predictive Performance, Validation, and Clinical Readiness
Source: J Clin Med. 2026 Oct 4;15(19):7692. doi: 10.3390/jcm15197692 (PMC13666408; doi:10.3390/jcm15197692)
Supplement: Supplementary file 1 [file jcm-15-07692-s001.zip › File S1-PRISMA Checklist.pdf]

PRISMA 2020 Checklist

Section and Topic	Item #	Checklist item	Location where item is reported
**TITLE**			
Title	1	Identify the report as a systematic review.	Main manuscript: Title, p. 1.
**ABSTRACT**			
Abstract	2	See the PRISMA 2020 for Abstracts checklist.	Main manuscript: Abstract, p. 2.
**INTRODUCTION**			
Rationale	3	Describe the rationale for the review in the context of existing knowledge.	Main manuscript: Introduction, pp. 3-5.
Objectives	4	Provide an explicit statement of the objective(s) or question(s) the review addresses.	Main manuscript: Introduction (review aims), p. 5.
**METHODS**			
Eligibility criteria	5	Specify the inclusion and exclusion criteria for the review and how studies were grouped for the syntheses.	Main manuscript: Methods 2.2-2.3, pp. 5-6.
Information sources	6	Specify all databases, registers, websites, organisations, reference lists and other sources searched or consulted to identify studies. Specify the date when each source was last searched or consulted.	Main manuscript: Methods 2.1, p. 5 (PubMed/MEDLINE, Embase, Scopus, Web of Science; searched through Aug 6, 2026).
Search strategy	7	Present the full search strategies for all databases, registers and websites, including any filters and limits used.	Main manuscript: Methods 2.1, p. 5. Supplement: Table S1, p. 2 (full database-specific strategies).
Selection process	8	Specify the methods used to decide whether a study met the inclusion criteria of the review, including how many reviewers screened each record and each report retrieved, whether they worked independently, and if applicable, details of automation tools used in the process.	Main manuscript: Methods 2.2, p. 6 (3 independent screeners; Rayyan; consensus/fourth-reviewer resolution).
Data collection process	9	Specify the methods used to collect data from reports, including how many reviewers collected data from each report, whether they worked independently, any processes for obtaining or confirming data from study investigators, and if applicable, details of automation tools used in the process.	Main manuscript: Methods 2.2, p. 6; Supplement: Tables S2-S3, pp. 3-17. PARTIAL: number/independence of data extractors and investigator contact procedures are not stated.
Data items	10a	List and define all outcomes for which data were sought. Specify whether all results that were compatible with each outcome domain in each study were sought (e.g. for all measures, time points, analyses), and if not, the methods used to decide which results to collect.	Main manuscript: Methods 2.2-2.3, pp. 5-6 (outcome domains, horizons, and outcome-grouping rules).
	10b	List and define all other variables for which data were sought (e.g. participant and intervention characteristics, funding sources). Describe any assumptions made about any missing or unclear information.	Main manuscript: Methods 2.2, p. 6; Supplement: Tables S2-S3, pp. 3-17. PARTIAL: variables are listed, but assumptions for missing/unclear information are not explicitly stated.
Study risk of bias assessment	11	Specify the methods used to assess risk of bias in the included studies, including details of the tool(s) used, how many reviewers assessed each study and whether they worked independently, and if applicable, details of automation tools used in the process.	Main manuscript: Methods 2.3, p. 6; Supplement: Table S4, pp. 18-19. PARTIAL: PROBAST+AI-aligned approach is described, but number/independence of risk-of-bias assessors is not stated.
Effect measures	12	Specify for each outcome the effect measure(s) (e.g. risk ratio, mean difference) used in the synthesis or presentation of results.	Main manuscript: Methods 2.3-2.5, pp. 6-9 (AUC/C-statistic; sensitivity/specificity and SROC for threshold-level analyses).
Synthesis methods	13a	Describe the processes used to decide which studies were eligible for each synthesis (e.g. tabulating the study intervention characteristics and comparing against the planned groups for each synthesis (item #5)).	Main manuscript: Methods 2.2-2.5, pp. 5-9 (clinical exchangeability, outcome grouping, source-family independence, eligible operating points). Supplement: Table S2, pp. 3-12.
	13b	Describe any methods required to prepare the data for presentation or synthesis, such as handling of missing summary statistics, or data conversions.	Main manuscript: Methods 2.4-2.5, pp. 6-9 (logit-AUC conversion, CI/SE reconstruction, delta method, continuity correction).

PRISMA 2020 Checklist

Section and Topic	Item #	Checklist item	Location where item is reported
	13c	Describe any methods used to tabulate or visually display results of individual studies and syntheses.	Main manuscript: Methods 2.6, p. 9; Figures 1-8, pp. 11-21. Supplement: Figures S1-S16, pp. 26-41.
	13d	Describe any methods used to synthesize results and provide a rationale for the choice(s). If meta-analysis was performed, describe the model(s), method(s) to identify the presence and extent of statistical heterogeneity, and software package(s) used.	Main manuscript: Methods 2.4-2.7, pp. 6-10 (REML random effects, modified Hartung-Knapp, heterogeneity statistics, CR2/multilevel and Bayesian models, software).
	13e	Describe any methods used to explore possible causes of heterogeneity among study results (e.g. subgroup analysis, meta-regression).	Main manuscript: Methods 2.4-2.5, pp. 7-8 (prespecified subgroup analyses and meta-regression moderators; Bayesian meta-regression).
	13f	Describe any sensitivity analyses conducted to assess robustness of the synthesized results.	Main manuscript: Methods 2.6, p. 9 (leave-one-out, influence, source-family/horizon alternatives, uncertainty inflation, estimator/inference sensitivity).
Reporting bias assessment	14	Describe any methods used to assess risk of bias due to missing results in a synthesis (arising from reporting biases).	Main manuscript: Methods 2.6, p. 9 (standard and contour-enhanced funnel plots; cautious interpretation of asymmetry).
Certainty assessment	15	Describe any methods used to assess certainty (or confidence) in the body of evidence for an outcome.	NOT ASSESSED/REPORTED: no formal certainty-of-evidence framework (e.g., GRADE) is described.
**RESULTS**			
Study selection	16a	Describe the results of the search and selection process, from the number of records identified in the search to the number of studies included in the review, ideally using a flow diagram.	Main manuscript: Results 3.1, pp. 10-11; Figure 1 (PRISMA 2020 flow diagram), p. 11.
	16b	Cite studies that might appear to meet the inclusion criteria, but which were excluded, and explain why they were excluded.	Main manuscript: Figure 1, p. 11 reports aggregate full-text exclusion reasons. PARTIAL: citation-level listing of potentially eligible excluded reports is not provided.
Study characteristics	17	Cite each included study and present its characteristics.	Main manuscript: Results 3.1, pp. 10-12. Supplement: Table S2, pp. 3-12 (characteristics of all 67 included reports).
Risk of bias in studies	18	Present assessments of risk of bias for each included study.	Main manuscript: Results 3.1 and 3.4, pp. 11 and 17-18. Supplement: Table S4, pp. 18-19; Figure S6, p. 31 (study-level assessments).
Results of individual studies	19	For all outcomes, present, for each study: (a) summary statistics for each group (where appropriate) and (b) an effect estimate and its precision (e.g. confidence/credible interval), ideally using structured tables or plots.	Main manuscript: Figures 3-8, pp. 14-21; Table 2, p. 22. Supplement: Tables S2, S5, S8 (pp. 3-12, 20, 23) and Figure S9, p. 34. PARTIAL for outcomes not entering quantitative synthesis.
Results of syntheses	20a	For each synthesis, briefly summarise the characteristics and risk of bias among contributing studies.	Main manuscript: Results 3.1-3.7, pp. 10-21; risk-of-bias synthesis in 3.4, pp. 17-18. Supplement: Tables S4-S8, pp. 18-23.
	20b	Present results of all statistical syntheses conducted. If meta-analysis was done, present for each the summary estimate and its precision (e.g. confidence/credible interval) and measures of statistical heterogeneity. If comparing groups, describe the direction of the effect.	Main manuscript: Results 3.2-3.8, pp. 13-22; Figures 3-8. Supplement: Tables S5-S9, pp. 20-24; Figures S3-S15, pp. 28-40.
	20c	Present results of all investigations of possible causes of heterogeneity among study results.	Main manuscript: Results 3.2-3.4, pp. 15-18 (validation, timing,

PRISMA 2020 Checklist

Section and Topic	Item #	Checklist item	Location where item is reported
			horizon, modality, region, sample size, prevalence, risk of bias). Supplement: Table S6, p. 21; Figures S4-S8, pp. 29-33.
	20d	Present results of all sensitivity analyses conducted to assess the robustness of the synthesized results.	Main manuscript: Results 3.8, p. 22. Supplement: Table S9, p. 24; Figures S11-S15, pp. 36-40.
Reporting biases	21	Present assessments of risk of bias due to missing results (arising from reporting biases) for each synthesis assessed.	Main manuscript: Results 3.8, p. 22 (funnel asymmetry assessment and SE moderator). Supplement: Figure S16, p. 41.
Certainty of evidence	22	Present assessments of certainty (or confidence) in the body of evidence for each outcome assessed.	NOT ASSESSED/REPORTED: no formal certainty/confidence assessment of the body of evidence is presented.
**DISCUSSION**			
Discussion	23a	Provide a general interpretation of the results in the context of other evidence.	Main manuscript: Discussion 4.1-4.6, pp. 23-27.
	23b	Discuss any limitations of the evidence included in the review.	Main manuscript: Discussion 4.7 (Strengths and limitations), p. 27.
	23c	Discuss any limitations of the review processes used.	Main manuscript: Discussion 4.7, p. 27 (aggregate-data limitations, incomplete reporting, source-family uncertainty, reconstructed precision, study-level meta-regression limitations).
	23d	Discuss implications of the results for practice, policy, and future research.	Main manuscript: Discussion 4.5-4.7, pp. 26-27; Conclusion, p. 28.
**OTHER INFORMATION**			
Registration and protocol	24a	Provide registration information for the review, including register name and registration number, or state that the review was not registered.	Main manuscript: Methods 2.8, p. 10 (International Research Registry; reviewregistry2130; registered Aug 18, 2026 after the database search).
	24b	Indicate where the review protocol can be accessed, or state that a protocol was not prepared.	Main manuscript: Methods 2.8, p. 10 (protocol access link provided).
	24c	Describe and explain any amendments to information provided at registration or in the protocol.	Main manuscript: Methods 2.8, p. 10 states that registration occurred after the database search. PARTIAL: amendments/deviations from the registered protocol are not otherwise described.
Support	25	Describe sources of financial or non-financial support for the review, and the role of the funders or sponsors in the review.	Main manuscript: Funding statement, p. 28 (no financial support reported).
Competing interests	26	Declare any competing interests of review authors.	Main manuscript: Declarations - Conflict of interest, p. 28.
Availability of data, code and other materials	27	Report which of the following are publicly available and where they can be found: template data collection forms; data extracted from included studies; data used for all analyses; analytic code; any other materials used in the review.	Main manuscript: Methods 2.7, pp. 9-10; Availability of data and materials, p. 28. PARTIAL: data are stated to be available on request and a reproducibility package was retained, but no public repository/location for data/code is specified.

From: Page MJ, McKenzie JE, Bossuyt PM, Boutron I, Hoffmann TC, Mulrow CD, et al. The PRISMA 2020 statement: an updated guideline for reporting systematic reviews. BMJ 2021;372:n71. doi: 10.1136/bmj.n71.
This work is licensed under CC BY 4.0. To view a copy of this license, visit https://creativecommons.org/licenses/by/4.0/
