# Supplementary material for: Machine Learning for Clinical Outcome Prediction After Transcatheter Aortic Valve Replacement: A Systematic Review and Meta-Analysis of Predictive Performance, Validation, and Clinical Readiness
Source: J Clin Med. 2026 Oct 4;15(19):7692. doi: 10.3390/jcm15197692 (PMC13666408; doi:10.3390/jcm15197692)

Supplementary Tables and Figures

Machine Learning for Clinical Outcome Prediction After Transcatheter Aortic Valve Replacement: A Systematic Review and Meta-Analysis of Predictive Performance, Validation, and Clinical Readiness

Azin Alizadehasl; Elahe Abdi Bastami; Seyedeh Fatemeh Hosseini Jebelli; Delaram Narimani Davani; Alia Bahramnejad; Azam Yalameh Aliabadi; Seyyedeh Maryam Moussavi; Azin Ghaffari; Reza Amani-Beni; Bahar Darouei; Ehsan Amini-Salehi; Seyyed Mohammad Hashemi; Samira Golkar; Hoda Hakimian; Soroush Najdaghi

Supplementary Table S1. Database-specific search strategies for the final August 6, 2026 update

Database	Search strategy
**PubMed/MEDLINE**	(("Aortic Valve Stenosis"[Mesh] OR "aortic stenosis"[tiab] OR "Transcatheter Aortic Valve Replacement"[Mesh] OR "transcatheter aortic valve replacement"[tiab] OR "transcatheter aortic valve implantation"[tiab] OR TAVR[tiab] OR TAVI[tiab]) AND ("Machine Learning"[Mesh] OR "Artificial Intelligence"[Mesh] OR "Deep Learning"[Mesh] OR "machine learning"[tiab] OR "artificial intelligence"[tiab] OR "deep learning"[tiab] OR "neural network*"[tiab] OR "random forest*"[tiab] OR "gradient boosting"[tiab] OR XGBoost[tiab] OR "support vector machine*"[tiab] OR "decision tree*"[tiab] OR "ensemble learning"[tiab]) AND (mortality[tiab] OR survival[tiab] OR pacemaker[tiab] OR "permanent pacemaker"[tiab] OR conduction[tiab] OR "left bundle branch block"[tiab] OR stroke[tiab] OR "cerebrovascular event*"[tiab] OR paravalvular[tiab] OR "heart failure"[tiab] OR readmission[tiab] OR bleeding[tiab] OR vascular[tiab] OR complication*[tiab] OR outcome*[tiab] OR futility[tiab] OR discharge[tiab]))
**Embase**	('aortic valve stenosis'/exp OR 'transcatheter aortic valve implantation'/exp OR 'aortic stenosis':ti,ab OR tavi:ti,ab OR tavr:ti,ab OR 'transcatheter aortic valve replacement':ti,ab OR 'transcatheter aortic valve implantation':ti,ab) AND ('machine learning'/exp OR 'artificial intelligence'/exp OR 'deep learning'/exp OR 'neural network'/exp OR 'machine learning':ti,ab OR 'artificial intelligence':ti,ab OR 'deep learning':ti,ab OR 'random forest':ti,ab OR 'gradient boosting':ti,ab OR xgboost:ti,ab OR 'support vector machine':ti,ab OR 'decision tree':ti,ab OR 'ensemble learning':ti,ab) AND (mortality:ti,ab OR survival:ti,ab OR pacemaker:ti,ab OR conduction:ti,ab OR stroke:ti,ab OR 'cerebrovascular event':ti,ab OR paravalvular:ti,ab OR 'heart failure':ti,ab OR readmission:ti,ab OR bleeding:ti,ab OR vascular:ti,ab OR complication*:ti,ab OR outcome*:ti,ab OR futility:ti,ab OR discharge:ti,ab)
**Scopus**	TITLE-ABS-KEY((TAVI OR TAVR OR "transcatheter aortic valve replacement" OR "transcatheter aortic valve implantation" OR "aortic stenosis") AND ("machine learning" OR "artificial intelligence" OR "deep learning" OR "neural network*" OR "random forest*" OR "gradient boosting" OR XGBoost OR "support vector machine*" OR "decision tree*" OR "ensemble learning") AND (mortality OR survival OR pacemaker OR "permanent pacemaker" OR conduction OR "left bundle branch block" OR stroke OR "cerebrovascular event*" OR paravalvular OR "heart failure" OR readmission OR bleeding OR vascular OR complication* OR outcome* OR futility OR discharge))
**Web of Science Core Collection**	TS=((TAVI OR TAVR OR "transcatheter aortic valve replacement" OR "transcatheter aortic valve implantation" OR "aortic stenosis") AND ("machine learning" OR "artificial intelligence" OR "deep learning" OR "neural network*" OR "random forest*" OR "gradient boosting" OR XGBoost OR "support vector machine*" OR "decision tree*" OR "ensemble learning") AND (mortality OR survival OR pacemaker OR "permanent pacemaker" OR conduction OR "left bundle branch block" OR stroke OR "cerebrovascular event*" OR paravalvular OR "heart failure" OR readmission OR bleeding OR vascular OR complication* OR outcome* OR futility OR discharge))

Searches were translated across database-specific controlled vocabulary and field syntax while preserving the same three concepts (TAVR (including TAVI terminology), artificial intelligence and machine learning, and clinically relevant post-TAVR outcomes). Syntax is normalized here for reproducibility; native-interface automatic term mapping and formatting may differ in the archived search history. The final database export files remain the audit trail for record counts and deduplication.

Supplementary Table S2. Full-text source-audited study, outcome, model, and evaluation characteristics of the 67 included reports

ID	Source-family ID | Study / year / title / DOI	Design / setting	Population + evaluation information base	Event frequency	Outcome + horizon	Predictor timing + modality	Algorithm families	Validation / performance provenance / source-family note
1	**SF-01** | 2019 — Value of machine learning in predicting TAVI outcomes<br>DOI: 10.1007/s12471-019-1285-7	Observational single-centre cohort	Population: n=1,478 source cohort; mortality status available for n=1,400; dyspnoea follow-up n=766<br>Evaluation: NR / resampling-based or no distinct evaluation cohort explicitly adjudicated	1-year cardiovascular mortality: 137/1,400 (9.8%); dyspnoea analysis: 605/766 improved, 161/766 did not improve	Cardiovascular mortality; dyspnoea improvement<br>Horizon: 1 year mortality; 60-day NYHA/dyspnoea	Mixed preprocedural + peri/postprocedural<br>CT/imaging; Echocardiography; ECG; Clinical/laboratory	Logistic regression; random forest; support vector machine; multilayer perceptron / neural network; additional conventional ML classifier(s) as reported	5-fold cross-validation<br>Performance: Reported; representative estimate retained in outcome-level analysis dataset/NR here<br>Audit/source-family: Amsterdam/Catharina inter-hospital family: likely overlapping institutional data; preserve family audit
2	**SF-02** | 2021 — Towards patient-specific prediction of conduction abnormalities induced by transcatheter aortic valve implantation: a combined mechanistic modelling and machine learning approach<br>DOI: 10.1093/ehjdh/ztab063	Multicentre observational study	Population: n=189<br>Evaluation: NR / resampling-based or no distinct evaluation cohort explicitly adjudicated	Conduction abnormality in analysed population: 89/151 (59%); no conduction abnormality 62/151 (41%)	Post-TAVI conduction abnormality / AV conduction disturbance<br>Horizon: Early/postprocedural	Mixed preprocedural + peri/postprocedural<br>CT/imaging; ECG; Biomechanical/mechanistic; Clinical/laboratory	LR; RF; SVM; GBM/boosting; DT; KNN	bootstrap<br>Performance: Reported; representative estimate retained in outcome-level analysis dataset/NR here<br>Audit/source-family: No specific overlap flag in current source-family audit
3	**SF-03** | 2023 — Temporal validation of 30-day mortality prediction models for transcatheter aortic valve implantation using statistical process control – An observational study in a national population<br>DOI: 10.1016/j.heliyon.2023.e17139	National multisite temporal-validation study	Population: 12,440 TAVI records/procedures assessed in the temporal-validation dataset; period-specific analysis per report<br>Evaluation: NR / resampling-based or no distinct evaluation cohort explicitly adjudicated	30-day mortality: 410/11,291 (3.36%) in the analysed national population	All-cause mortality<br>Horizon: 30 days	Mixed preprocedural + peri/postprocedural<br>Clinical/laboratory	Logistic regression; XGBoost	Temporal validation; External/independent validation; 10-fold crossvalidation<br>Performance: Reported; representative estimate retained in outcome-level analysis dataset/NR here<br>Audit/source-family: No specific overlap flag in current source-family audit
4	**SF-04** | 2023 — Prediction of permanent pacemaker implantation after transcatheter aortic valve replacement: The role of machine learning<br>DOI: 10.4330/wjc.v15.i3.95	Retrospective multicentre cohort (Mayo Clinic Rochester/Phoenix/Jacksonville)	Population: n=1,200 retrospective source cohort; n=964 analysed for model development/evaluation after eligibility filtering<br>Evaluation: NR / resampling-based or no distinct evaluation cohort explicitly adjudicated	NR / not explicitly resolved in the study-level table; retained in QA evidence	Permanent pacemaker implantation<br>Horizon: Post-TAVR; study-defined follow-up	Mixed preprocedural + peri/postprocedural<br>CT/imaging; Echocardiography; Clinical/laboratory	Random forest; Gradient boosting	5-fold cross-validation<br>Performance: Reported; representative estimate retained in outcome-level analysis dataset/NR here<br>Audit/source-family: No specific overlap flag in current source-family audit
5	**SF-05** | 2023 — Prediction Model of Post-TAVR Complication Using a Medical Twin Web Navigator<br>DOI: 10.13052/jwe1540-9589.2274	Observational/design NR	Population: n=43<br>Evaluation: NR / resampling-based or no distinct evaluation cohort explicitly adjudicated	NR / not explicitly resolved in the study-level table; retained in QA evidence	Post-TAVR complications (multi-outcome)<br>Horizon: Postprocedural; study-defined	Mixed preprocedural + peri/postprocedural<br>CT/imaging; ECG; Clinical/laboratory	Logistic regression; decision tree; random forest; XGBoost / gradient boosting	Held-out validation/test set<br>Performance: Reported; representative estimate retained in outcome-level analysis dataset/NR here<br>Audit/source-family: No specific overlap flag in current source-family audit
6	**SF-06** | 2024 — Predicting mortality after transcatheter aortic valve replacement using preprocedural CT<br>DOI: 10.1038/s41598-024-63022-x	Retrospective single-centre analysis of prospectively enrolled SwissTAVI registry participants	Population: n=741<br>Evaluation: Primary mortality evaluation information base: n=1449; events=NR; 10-fold CV full cohort; mortality prevalence reported 44%	Primary mortality evaluation event prevalence: 44.0%	All-cause mortality<br>Horizon: Long-term; study-defined CT survival endpoint	Mixed preprocedural + peri/postprocedural<br>CT/imaging; Clinical/laboratory	Neural network / deep learning	10-fold cross-validation<br>Performance: Reported; representative estimate retained in outcome-level analysis dataset/NR here<br>Audit/source-family: No specific overlap flag in current source-family audit
7	**SF-07** | 2021 — Predicting Long-Term Mortality in TAVI Patients Using Machine Learning Techniques<br>DOI: 10.3390/jcdd8040044	Retrospective single-centre cohort	Population: n=511 source cohort; n=471 reported in complete analysed subset<br>Evaluation: Primary mortality evaluation information base: n=471; events=212; Stratified 10-fold CV full cohort	Primary mortality evaluation: 212/471 (45.0%)	All-cause mortality<br>Horizon: Long-term; study-defined	Mixed preprocedural + peri/postprocedural<br>CT/imaging; Echocardiography; Clinical/laboratory; Administrative	Logistic regression; random forest; XGBoost; multilayer perceptron	Bootstrap<br>Performance: Reported; representative estimate retained in outcome-level analysis dataset/NR here<br>Audit/source-family: No specific overlap flag in current source-family audit
8	**SF-08** | 2023 — Predicting in-hospital mortality after transcatheter aortic valve replacement using administrative data and machine learning<br>DOI: 10.1038/s41598-023-37358-9	Retrospective nationwide administrative (NIS) cohort; multisite	Population: n=54,739<br>Evaluation: Primary mortality evaluation information base: n=13982; events=176; 2019 temporal test set	Primary mortality evaluation: 176/13982 (1.3%)	In-hospital mortality<br>Horizon: Index hospitalization	Mixed preprocedural + peri/postprocedural<br>Clinical/laboratory; Administrative	L2-regularized logistic regression; LightGBM; CatBoost; additional benchmark ML models	holdout<br>Performance: Reported; representative estimate retained in outcome-level analysis dataset/NR here<br>Audit/source-family: No specific overlap flag in current source-family audit

ID	Source-family ID | Study / year / title / DOI	Design / setting	Population + evaluation information base	Event frequency	Outcome + horizon	Predictor timing + modality	Algorithm families	Validation / performance provenance / source-family note
9	**SF-09** | 2024 — Performance of federated learning-based models in the Dutch TAVI population was comparable to central strategies and outperformed local strategies<br>DOI: 10.3389/fcvm.2024.1399138	Retrospective; multicentre; registry	Population: n=16,661 TAVI records analysed in nationwide multicentre cohort<br>Evaluation: Primary mortality evaluation information base: n=16661; events=NR; Leave-centre-out national registry; mortality prevalence reported 3.4%	30-day mortality: 3.4% of 16,661 TAVI records | Primary mortality evaluation event prevalence: 3.4%	Mortality / TAVI outcome prediction in federated setting<br>Horizon: Study-defined	Preprocedural/baseline<br>Clinical/laboratory	Federated learning	Temporal validation; Geographic validation; External/independent validation; train/test<br>Performance: Reported; representative estimate retained in outcome-level analysis dataset/NR here<br>Audit/source-family: No specific overlap flag in current source-family audit
10	**SF-10** | 2024 — Multilayer Perceptron Neural Network Analysis of Fluoroscopic Working Angle on Transcatheter Aortic Valve Implantation Complications<br>DOI: 10.7759/cureus.59144	Retrospective single-centre cohort	Population: n=834<br>Evaluation: Primary mortality evaluation information base: n=250; events=6; 30% held-out test set; exact event count implied by locked HM variance	Primary mortality evaluation: 6/250 (2.4%)	TAVI complications by fluoroscopic working angle<br>Horizon: Periprocedural/postprocedural	Mixed preprocedural + peri/postprocedural<br>Fluoroscopy/procedural imaging; Clinical/laboratory	Neural network / deep learning	train test<br>Performance: Reported; representative estimate retained in outcome-level analysis dataset/NR here<br>Audit/source-family: No specific overlap flag in current source-family audit
11	**SF-11** | 2019 — Machine Learning Prediction Models for In-Hospital Mortality After Transcatheter Aortic Valve Replacement<br>DOI: 10.1016/j.jcin.2019.06.013	Retrospective nationwide administrative (NIS) cohort; multisite	Population: n=10,883<br>Evaluation: NR / resampling-based or no distinct evaluation cohort explicitly adjudicated	In-hospital mortality: 3.6% of 10,883	In-hospital mortality<br>Horizon: Index hospitalization	Mixed preprocedural + peri/postprocedural<br>Clinical/laboratory; Administrative	Logistic regression; Random forest; Neural network / deep learning; Naive Bayes	Held-out validation/test set<br>Performance: Reported; representative estimate retained in outcome-level analysis dataset/NR here<br>Audit/source-family: No specific overlap flag in current source-family audit
12	SF-12 | 2021 — Machine learning method for predicting pacemaker implantation following transcatheter aortic valve replacement<br>DOI: 10.1111/pace.14163	Retrospective	Population: n=557<br>Evaluation: internal model evaluation; no independent external cohort reported	PPI: 95/557 (17.1%)	Permanent pacemaker implantation<br>Horizon: Post-TAVR; study-defined	Mixed preprocedural + early postprocedural<br>Echocardiography; ECG; Clinical/laboratory	Logistic regression; Random forest	Internal model evaluation; RF AUC 0.81 with post-TAVR ECG vs 0.72 without; logistic regression AUC 0.69<br>Audit/source-family: SF-12 representative strict-PPMI effect; report 42 is linked reuse and not counted independently.
13	**SF-13** | 2023 — Machine learning for prediction of all-cause mortality after transcatheter aortic valve implantation<br>DOI: 10.1093/ehjqcco/qcad002	Multicentre cohort (derivation + independent validation)	Population: Derivation n=604; independent validation n=823<br>Evaluation: n=823 external	1-year deaths: 68/604 (11%) derivation; 95/823 (12%) validation	All-cause mortality<br>Horizon: Study-defined follow-up	Mixed preprocedural + peri/postprocedural<br>Echocardiography; Fluoroscopy/procedural imaging; Clinical/laboratory	XGBoost; Gradient boosting	External/independent validation; hold-out<br>Performance: Reported; representative estimate retained in outcome-level analysis dataset/NR here<br>Audit/source-family: Kwiecinski/Warsaw mortality family: overlapping derivation era; one representative in primary mortality
14	**SF-01** | 2021 — Machine Learning for Predicting Mortality in Transcatheter Aortic Valve Implantation: An Inter-Center Cross Validation Study<br>DOI: 10.3390/jcdd8060065	Observational/design NR; multicentre	Population: Two-centre cohort, total n=1,171<br>Evaluation: Primary mortality evaluation information base: n=631; events=67; External CZE-TU/e validation centre	Primary mortality evaluation: 67/631 (10.6%)	Mortality<br>Horizon: Study-defined; inter-centre validation	Periprocedural<br>Clinical/laboratory; Administrative	Logistic regression; random forest; CatBoost / gradient boosting; XGBoost comparator	Inter-centre/site validation; External/independent validation; Cross-validation (fold scheme not resolved)<br>Performance: Reported; representative estimate retained in outcome-level analysis dataset/NR here<br>Audit/source-family: Amsterdam/Catharina inter-hospital family: likely overlapping institutional data; preserve family audit
15	**SF-15** | 2024 — Machine learning for pacemaker implantation prediction after TAVI using multimodal imaging data<br>DOI: 10.1038/s41598-024-76128-z	Retrospective single-centre cohort; CT measurements prospectively acquired/blinded	Population: n=5,740<br>Evaluation: NR / resampling-based or no distinct evaluation cohort explicitly adjudicated	NR / not explicitly resolved in the study-level table; retained in QA evidence	Permanent pacemaker implantation<br>Horizon: Post-TAVI; study-defined	Mixed preprocedural + peri/postprocedural<br>CT/imaging; Echocardiography; ECG; Clinical/laboratory	Logistic regression; Random forest; Support vector machine; XGBoost; Gradient boosting; Neural network / deep learning; Decision tree; k-nearest neighbours	nested cross-validation<br>Performance: Reported; representative estimate retained in outcome-level analysis dataset/NR here<br>Audit/source-family: No specific overlap flag in current source-family audit
16	**SF-16** | 2020 — Machine learning-based risk prediction of intrahospital clinical outcomes in patients undergoing TAVI<br>DOI: 10.1007/s00392-020-01691-0	Retrospective	Population: n=451 analysed<br>Evaluation: Primary mortality evaluation information base: n=451; events=25; Nested 5-fold CV full cohort	Primary mortality evaluation: 25/451 (5.5%)	Intrahospital clinical outcomes (multi-outcome)<br>Horizon: Index hospitalization	Mixed preprocedural + peri/postprocedural<br>CT/imaging; Echocardiography; ECG; Fluoroscopy/procedural imaging; Clinical/laboratory	Neural network; support vector machine; random forest; XGBoost feature-selection/modeling component	nested cross-validation<br>Performance: Reported; representative estimate retained in outcome-level analysis dataset/NR here<br>Audit/source-family: No specific

ID	Source-family ID | Study / year / title / DOI	Design / setting	Population + evaluation information base	Event frequency	Outcome + horizon	Predictor timing + modality	Algorithm families	Validation / performance provenance / source-family note
								overlap flag in current source-family audit
17	**SF-17** | 2022 — Machine learning-based predictive risk models for 30-day and 1-year mortality in severe aortic stenosis patients undergoing transcatheter aortic valve implantation<br>DOI: 10.1016/j.ijcard.2022.12.023	Retrospective	Population: n=186<br>Evaluation: Primary mortality evaluation information base: n=178; events=24; Complete-data cohort repeatedly split 80:20	Mortality: 7/186 (3.9%) by 30 days; 24/186 (13.5%) by 1 year | Primary mortality evaluation: 24/178 (13.5%)	All-cause mortality<br>Horizon: 30 days and 1 year	Mixed preprocedural + peri/postprocedural<br>ECG; Clinical/laboratory	Decision-tree models; random-forest-based imputation/supporting modeling	training/test<br>Performance: Reported; representative estimate retained in outcome-level analysis dataset/NR here<br>Audit/source-family: No specific overlap flag in current source-family audit
18	**SF-01** | 2021 — Local and Distributed Machine Learning for Inter-hospital Data Utilization: An Application for TAVI Outcome Prediction<br>DOI: 10.3389/fcvm.2021.787246	Two-centre distributed analysis using a dedicated prospective TAVI registry	Population: Two-centre distributed dataset, total n=1,791 (including AMC n=1,160)<br>Evaluation: NR / resampling-based or no distinct evaluation cohort explicitly adjudicated	NR / not explicitly resolved in the study-level table; retained in QA evidence	TAVI outcome prediction across hospitals<br>Horizon: Study-defined	Mixed preprocedural + peri/postprocedural<br>NR	Local/distributed XGBoost; distributed neural network; logistic-regression comparator	5-fold cross-validation<br>Performance: Reported; representative estimate retained in outcome-level analysis dataset/NR here<br>Audit/source-family: Amsterdam/Catharina inter-hospital family: likely overlapping institutional data; preserve family audit
19	**SF-19** | 2023 — Inflammatory Status Assessment by Machine Learning Techniques to Predict Outcomes in Patients with Symptomatic Aortic Stenosis Treated by Transcatheter AorticValve Replacement<br>DOI: 10.3390/diagnostics13182907	Retrospective	Population: n=338; 56 deaths (16.5%) during follow-up<br>Evaluation: Primary mortality evaluation information base: n=338; events=56; 5-fold pooled testing predictions	Deaths during follow-up: 56/338 (16.5%) | Primary mortality evaluation: 56/338 (16.6%)	Clinical outcomes using inflammatory signatures<br>Horizon: Post-TAVR; study-defined	Mixed preprocedural + peri/postprocedural<br>CT/imaging; Echocardiography; Clinical/laboratory	Random forest; Support vector machine; Gradient boosting	5-fold cross-validation<br>Performance: Reported; representative estimate retained in outcome-level analysis dataset/NR here<br>Audit/source-family: No specific overlap flag in current source-family audit
20	**SF-20** | 2023 — Direct deep learning-based survival prediction from pre-interventional CT prior to transcatheter aortic valve replacement<br>DOI: 10.1016/j.ejrad.2023.111150	Retrospective; single-centre	Population: n=760<br>Evaluation: NR / resampling-based or no distinct evaluation cohort explicitly adjudicated	Deaths during follow-up: 54% of 760	Survival / mortality<br>Horizon: Long-term survival	Preprocedural/baseline<br>CT/imaging; Clinical/laboratory	Neural network / deep learning; Cox model	holdout<br>Performance: Reported; representative estimate retained in outcome-level analysis dataset/NR here<br>Audit/source-family: No specific overlap flag in current source-family audit
21	**SF-21** | 2022 — Development of an Electronic Frailty Index for Predicting Mortality in Patients Undergoing Transcatheter Aortic Valve Replacement using Machine Learning<br>DOI: 10.4103/ACCJ.ACCJ_13_22	Retrospective multicentre observational cohort	Population: n=450<br>Evaluation: NR / resampling-based or no distinct evaluation cohort explicitly adjudicated	Deaths during follow-up: 22/450	Mortality<br>Horizon: Post-TAVR; study-defined	Preprocedural/baseline<br>ECG; Clinical/laboratory	Cox regression; gradient-boosted survival trees; random survival forest	Cross-validation (fold scheme not resolved)<br>Performance: Reported; representative estimate retained in outcome-level analysis dataset/NR here<br>Audit/source-family: No specific overlap flag in current source-family audit
22	**SF-22** | 2023 — Development and validation of explainable machine learning models for risk of mortality in transcatheter aortic valve implantation: TAVI risk machine scores<br>DOI: 10.1093/ehjdh/ztad021	Retrospective GARY analysis with external validation in prospective national SwissTAVI registry	Population: GARY training n=22,283; GARY test n=5,864; SwissTAVI external validation n=6,693<br>Evaluation: Primary mortality evaluation information base: n=5864; events=146; 2017 GARY temporal test set	30-day deaths: 729/22,283 training; 146/5,864 test; 205/6,693 Swiss external | Primary mortality evaluation: 146/5864 (2.5%)	Mortality<br>Horizon: Study-defined; development + validation	Mixed preprocedural + peri/postprocedural<br>Clinical/laboratory	Random forest (TAVI Risk Machine / TRIM framework)	training test<br>Performance: Reported; representative estimate retained in outcome-level analysis dataset/NR here<br>Audit/source-family: No specific overlap flag in current source-family audit
23	**SF-23** | 2023 — Development and Validation of a Machine Learning Risk-Prediction Model for 30-Day Readmission for Heart Failure Following Transcatheter Aortic Valve Replacement (TAVR-HF Score)<br>DOI: 10.1016/j.cpcardiol.2023.102143	Retrospective national administrative (NRD) cohort; multisite	Population: Administrative readmission cohort; study-specific 30-day analysis population reported in full text<br>Evaluation: NR / resampling-based or no distinct evaluation cohort explicitly adjudicated	NR / not explicitly resolved in the study-level table; retained in QA evidence	HF-related readmission<br>Horizon: 30 days	Mixed preprocedural + peri/postprocedural<br>Echocardiography; Clinical/laboratory; Administrative	Random-forest feature ranking + multivariable logistic regression score	Held-out validation/test set<br>Performance: Reported; representative estimate retained in outcome-level analysis dataset/NR here<br>Audit/source-family: No specific overlap flag in current source-family audit
24	**SF-24** | 2021 — Deep learning-based prediction of early cerebrovascular events after transcatheter aortic valve replacement<br>DOI: 10.1038/s41598-021-98265-5	Prospective single-centre registry cohort	Population: n=2,279<br>Evaluation: NR / resampling-based or no distinct evaluation cohort explicitly adjudicated	NR / not explicitly resolved in the study-level table; retained in QA evidence	Cerebrovascular event<br>Horizon: Early post-TAVR	Mixed preprocedural + peri/postprocedural<br>CT/imaging; Echocardiography; ECG; Clinical/laboratory	Logistic regression; Neural network / deep learning	Held-out validation/test set<br>Performance: Reported; representative estimate retained in outcome-level analysis dataset/NR here<br>Audit/source-family: No specific overlap flag in current source-family audit

ID	Source-family ID | Study / year / title / DOI	Design / setting	Population + evaluation information base	Event frequency	Outcome + horizon	Predictor timing + modality	Algorithm families	Validation / performance provenance / source-family note
25	**SF-25** | 2021 — Decision Trees for Predicting Mortality in Transcatheter Aortic Valve Implantation DOI: 10.3390/bioengineering8020022	Retrospective	Population: n=1,830 Evaluation: NR / resampling-based or no distinct evaluation cohort explicitly adjudicated	NR / not explicitly resolved in the study-level table; retained in QA evidence	Mortality Horizon: Study-defined	Mixed preprocedural + peri/postprocedural Echocardiography; Clinical/laboratory; Administrative	Gradient boosting on decision trees / CatBoost-type classifier	10-fold cross-validation Performance: Reported; representative estimate retained in outcome-level analysis dataset/NR here Audit/source-family: No specific overlap flag in current source-family audit
26	**SF-26** | 2021 — Biomechanical Identification of High-Risk Patients Requiring Permanent Pacemaker After Transcatheter Aortic Valve Replacement DOI: 10.3389/fbioe.2021.615090	Observational/design NR	Population: n=48 Evaluation: NR / resampling-based or no distinct evaluation cohort explicitly adjudicated	NR / not explicitly resolved in the study-level table; retained in QA evidence	Permanent pacemaker requirement Horizon: Post-TAVR	Mixed preprocedural + peri/postprocedural CT/imaging; Echocardiography; ECG; Biomechanical/mechanistic; Clinical/laboratory	LR; NN/DL; DT	leave-one-out cross validation Performance: Reported; representative estimate retained in outcome-level analysis dataset/NR here Audit/source-family: No specific overlap flag in current source-family audit
27	**SF-27** | 2020 — Artificial Intelligence Trumps TAVI2-SCORE and CoreValve Score in Predicting 1-Year Mortality Post-Transcatheter Aortic Valve Replacement DOI: 10.1016/j.carrev.2020.08.010	Retrospective multicentre cohort (Mayo Clinic, 3 sites)	Population: n=1,055 Evaluation: Primary mortality evaluation information base: n=1055; events=148; Repeated 5-fold CV full cohort	1-year mortality: 14.02% of 1,055 (148 deaths reported in study cohort) | Primary mortality evaluation: 148/1055 (14.0%)	All-cause mortality Horizon: 1 year	Mixed preprocedural + peri/postprocedural CT/imaging; Echocardiography; ECG; Clinical/laboratory	Gradient boosting	5-fold cross-validation Performance: Reported; representative estimate retained in outcome-level analysis dataset/NR here Audit/source-family: No specific overlap flag in current source-family audit
28	**SF-28** | 2023 — A Machine Learning Model for the Accurate Prediction of 1-Year Survival in TAVI Patients: A Retrospective Observational Cohort Study DOI: 10.3390/jcm12175481	Retrospective single-centre analysis; data prospectively collected in institutional database	Population: n=629 Evaluation: Primary mortality evaluation information base: n=565; events=67; 10-fold CV modelling cohort	1-year deaths: 67/565 (11.8%) among patients with complete 1-year survival status | Primary mortality evaluation: 67/565 (11.9%)	Survival / all-cause mortality Horizon: 1 year	Mixed preprocedural + peri/postprocedural CT/imaging; Echocardiography; ECG; Clinical/laboratory	Random forest; Support vector machine	10-fold cross-validation Performance: Reported; representative estimate retained in outcome-level analysis dataset/NR here Audit/source-family: No specific overlap flag in current source-family audit
29	**SF-29** | 2020 — Inflammatory signatures are associated with increased mortality after transfemoral transcatheter aortic valve implantation DOI: 10.1002/ehf2.12837	Observational/design NR; single-centre	Population: n=80 Evaluation: NR / resampling-based or no distinct evaluation cohort explicitly adjudicated	NR / not explicitly resolved in the study-level table; retained in QA evidence	12-month all-cause mortality Horizon: 12-month all-cause mortality	Mixed preprocedural + peri/postprocedural Clinical/laboratory	XGBoost gradient-boosted trees	Held-out validation/test set Performance: mortality (AUC 0.66±0.10 Audit/source-family: No specific overlap flag in current source-family audit
30	**SF-30** | 2021 — Machine Learning Algorithms for Prediction of Permanent Pacemaker Implantation After Transcatheter Aortic Valve Replacement DOI: 10.1161/CIRCEP.120.008941	Single-centre retrospective derivation with prospective temporal validation cohort	Population: Derivation group A n=888; prospective validation group B n=272 Evaluation: n=272 prospective validation	Derivation cohort: PPM implantation 184/888 (20.7%); validation cohort reported separately	Permanent pacemaker implantation Horizon: Study-defined; see outcome definition	Mixed preprocedural + peri/postprocedural Clinical/laboratory	Multiple ML classifiers; leading models include simple logistic regression and locally weighted learning	External/independent validation Performance: validation AUCROC 0.82 Audit/source-family: No specific overlap flag in current source-family audit
31	**SF-31** | 2021 — Development and Validation of a Practical Model to Identify Patients at Risk of Bleeding After TAVR DOI: 10.1016/j.jcin.2021.03.024	Prospective multicentre registries (RISPEVA derivation; POL-TAVI external validation)	Population: Derivation n=5,185; independent validation cohort reported separately Evaluation: NR / resampling-based or no distinct evaluation cohort explicitly adjudicated	Derivation cohort: 30-day bleeding 216/5,185 (4.2%)	30-day bleeding Horizon: 30-day bleeding	Mixed preprocedural + peri/postprocedural CT/imaging; Clinical/laboratory	ML feature-selection pipeline + multivariable logistic-regression PREDICT-TAVR model	External/independent validation; bootstrap Performance: external AUC 0.78 (0.72–0.82) Audit/source-family: No specific overlap flag in current source-family audit
32	**SF-32** | 2022 — Development and Validation of a Machine Learning Score for Readmissions After Transcatheter Aortic Valve Implantation DOI: 10.1016/j.jacadv.2022.100060	Retrospective national administrative (NRD/SID) cohort; multisite	Population: 30-day cohort n=117,398; 90-day cohort n=93,800 Evaluation: NR / resampling-based or no distinct evaluation cohort explicitly adjudicated	30-day readmission 12.4%; 90-day readmission 17.4% (study-specific cohorts)	30-day readmission Horizon: 30-day readmission	Mixed preprocedural + peri/postprocedural Clinical/laboratory; Administrative	LASSO feature selection; LightGBM prediction model	External/independent validation Performance: AUC 0.74 (0.70–0.78) Audit/source-family: No specific overlap flag in current source-family audit
33	**SF-33** | 2022 — Predicting short-term outcomes after transcatheter aortic valve replacement for aortic stenosis DOI: 10.1016/j.ahj.2022.11.007	Integrated health-system cohort (KPNC); multicentre status not explicitly designated	Population: n=1,563 Evaluation: Primary mortality evaluation information base: n=1565; events=31; 5-fold CV full cohort	Primary mortality evaluation: 31/1565 (2.0%)	30-day mortality and patient-centred outcomes Horizon: 30-day mortality and patient-centred outcomes	Mixed preprocedural + peri/postprocedural Echocardiography; Clinical/laboratory; Administrative	Gradient boosting machine; conventional logistic/Cox comparators by outcome	Cross-validation (fold scheme not resolved) Performance: mortality AUC 0.60 Audit/source-family: No specific overlap flag in current source-family audit
34	**SF-34** | 2023 — MemGCN: memory-augmented graph neural network for predict conduction disturbance after transcatheter aortic valve replacement DOI: 10.1007/s10489-023-04877-x	Observational/design NR	Population: n=3,500 Evaluation: NR / resampling-based or no distinct evaluation cohort explicitly adjudicated	NR / not explicitly resolved in the study-level table; retained in QA evidence	Conduction disturbance Horizon: Study-defined; see outcome definition	Mixed preprocedural + peri/postprocedural CT/imaging; ECG; Clinical/laboratory	Memory-augmented graph neural network (MemGCN)	Cross-validation (fold scheme not resolved) Performance: Reported; representative estimate retained in outcome-level analysis dataset/NR here

ID	Source-family ID | Study / year / title / DOI	Design / setting	Population + evaluation information base	Event frequency	Outcome + horizon	Predictor timing + modality	Algorithm families	Validation / performance provenance / source-family note
								Audit/source-family: No specific overlap flag in current source-family audit
35	**SF-35** | 2024 — Electrocardiogram-based prediction of conduction disturbances after transcatheter aortic valve replacement with convolutional neural network<br>DOI: 10.1093/ehjdh/ztae007	Single-centre retrospective analysis of a prospectively designed West China TAVR registry	Population: n=1,176 analysed<br>Evaluation: NR / resampling-based or no distinct evaluation cohort explicitly adjudicated	NR / not explicitly resolved in the study-level table; retained in QA evidence	Conduction disturbance<br>Horizon: Study-defined; see outcome definition	Mixed preprocedural + peri/postprocedural<br>ECG; Clinical/laboratory	XGBoost; Neural network / deep learning	hold-out<br>Performance: Reported; representative estimate retained in outcome-level analysis dataset/NR here<br>Audit/source-family: No specific overlap flag in current source-family audit
36	**SF-36** | 2024 — Machine Learning Identification of Modifiable Predictors of Patient Outcomes After Transcatheter Aortic Valve Replacement<br>DOI: 10.1016/j.jacadv.2024.101116	Retrospective 21-hospital Biome dataset	Population: Total n=14,562; held-out test n=2,500<br>Evaluation: n=2,500 held-out test	NR / not explicitly resolved in the study-level table; retained in QA evidence	Excellent outcome composite<br>Horizon: Study-defined; see outcome definition	Mixed preprocedural + peri/postprocedural<br>Clinical/laboratory	Random forest; logistic-regression comparator	bootstrap<br>Performance: Reported; representative estimate retained in outcome-level analysis dataset/NR here<br>Audit/source-family: No specific overlap flag in current source-family audit
37	**SF-37** | 2024 — Prediction of Major Adverse Cardiac Events After Transcatheter Aortic Valve Implantation: A Machine Learning Approach with GRACE Score<br>DOI: 10.14744/SEMB.2024.00836	Retrospective multicentre observational cohort	Population: n=453<br>Evaluation: NR / resampling-based or no distinct evaluation cohort explicitly adjudicated	Primary 30-day MACE composite includes all-cause mortality, MI and CVE; component rates include mortality 4.2% and MI 2.4%	30-day MACE composite<br>Horizon: 30-day MACE composite	Mixed preprocedural + peri/postprocedural<br>Echocardiography; ECG; Clinical/laboratory	XGBoost; binomial logistic-regression comparator	Evaluation strategy NR/unclear<br>Performance: Reported; representative estimate retained in outcome-level analysis dataset/NR here<br>Audit/source-family: No specific overlap flag in current source-family audit
38	**SF-38** | 2025 — AI-based prediction of left bundle branch block risk post-TAVI using pre-implantation clinical parameters<br>DOI: 10.1080/14796678.2025.2498866	Retrospective/single-centre clinical cohort; no explicit multicentre outcome evaluation identified	Population: n=1,113<br>Evaluation: NR / resampling-based or no distinct evaluation cohort explicitly adjudicated	New-onset persistent LBBB: 15.29%	Persistent LBBB<br>Horizon: Study-defined; see outcome definition	Mixed preprocedural + peri/postprocedural<br>ECG; Clinical/laboratory	XGBoost	Cross-validation (fold scheme not resolved)<br>Performance: Reported; representative estimate retained in outcome-level analysis dataset/NR here<br>Audit/source-family: No specific overlap flag in current source-family audit
39	**SF-39** | 2025 — An Integrative Machine Learning Model for Predicting Early Safety Outcomes in Patients Undergoing Transcatheter Aortic Valve Implantation<br>DOI: 10.3390/medicina61030374	Retrospective; single-centre	Population: n=224 analysed<br>Evaluation: NR / resampling-based or no distinct evaluation cohort explicitly adjudicated	NR / not explicitly resolved in the study-level table; retained in QA evidence	Early safety composite<br>Horizon: Early safety composite	Mixed preprocedural + peri/postprocedural<br>CT/imaging; Echocardiography; Fluoroscopy/procedural imaging; Clinical/laboratory	Random forest	10-fold cross validation<br>Performance: Reported; representative estimate retained in outcome-level analysis dataset/NR here<br>Audit/source-family: No specific overlap flag in current source-family audit
40	**SF-40** | 2025 — Construction of Predictive Models for Cardiovascular Mortality by Machine Learning Approaches in Patients Who Underwent Transcatheter Aortic Valve Implantation<br>DOI: 10.1253/circrep.CR-24-0182	Observational/design NR; single-centre	Population: n=252<br>Evaluation: NR / resampling-based or no distinct evaluation cohort explicitly adjudicated	Cardiovascular deaths: 13/252 (5.2%)	Cardiovascular mortality<br>Horizon: Study-defined; see outcome definition	Preprocedural/baseline<br>Echocardiography; Clinical/laboratory	Logistic regression; Random forest; Random survival forest; Cox model	10-fold cross-validation<br>Performance: Reported; representative estimate retained in outcome-level analysis dataset/NR here<br>Audit/source-family: No specific overlap flag in current source-family audit
41	**SF-41** | 2024 — ECG, clinical and novel CT-imaging predictors of necessary pacemaker implantation after transfemoral aortic valve replacement<br>DOI: 10.1016/j.ejrad.2024.111835	Retrospective; single-centre	Population: n=2,105 screened; matched analysis 312 PPM vs 305 controls (n=617)<br>Evaluation: NR / resampling-based or no distinct evaluation cohort explicitly adjudicated	Matched design: 312 PPM cases vs 305 controls; observed prevalence intentionally altered by matching	Permanent pacemaker implantation<br>Horizon: Study-defined; see outcome definition	Mixed preprocedural + peri/postprocedural<br>CT/imaging; ECG; Clinical/laboratory	Scaled LASSO regression	10-fold cross-validation<br>Performance: Reported; representative estimate retained in outcome-level analysis dataset/NR here<br>Audit/source-family: No specific overlap flag in current source-family audit
42	**SF-12** | 2025 — Federated Learning Lifecycle Management for Distributed Medical Artificial Intelligence Applications: A Case Study on Post-Transcatheter Aortic Valve Replacement Complication Prediction Solution<br>DOI: 10.3390/app15010378	Observational/design NR	Population: n=553<br>Evaluation: NR / resampling-based or no distinct evaluation cohort explicitly adjudicated	NR / not explicitly resolved in the study-level table; retained in QA evidence	Post-TAVR PPI federated-learning reanalysis<br>Horizon: Study-defined; see outcome definition	Periprocedural<br>Clinical/laboratory	Federated/centralized XGBoost	Evaluation strategy NR/unclear<br>Performance: Reported; representative estimate retained in outcome-level analysis dataset/NR here<br>Audit/source-family: Christ Hospital PPMI/PPI family: secondary/federated reuse; do not count as independent same-endpoint evidence

ID	Source-family ID | Study / year / title / DOI	Design / setting	Population + evaluation information base	Event frequency	Outcome + horizon	Predictor timing + modality	Algorithm families	Validation / performance provenance / source-family note
43	**SF-43** | 2025 — Multimodal Machine Learning-Based Technical Failure Prediction in Patients Undergoing Transcatheter Aortic Valve Replacement<br>DOI: 10.1016/j.jacadv.2025.102168	Prospective registry cohort; Bern clinical outcome cohort single-centre	Population: n=2,937 consecutive TAVR patients<br>Evaluation: NR / resampling-based or no distinct evaluation cohort explicitly adjudicated	NR / not explicitly resolved in the study-level table; retained in QA evidence	VARC-3 cardiac/vascular technical failure<br>Horizon: Study-defined; see outcome definition	Mixed preprocedural + peri/postprocedural CT/imaging; Echocardiography; ECG; Clinical/laboratory	Random forest; LASSO logistic regression; logistic regression	holdout<br>Performance: Reported; representative estimate retained in outcome-level analysis dataset/NR here<br>Audit/source-family: No specific overlap flag in current source-family audit
44	**SF-44** | 2025 — Multimodal Visualization and Explainable Machine Learning–Driven Markers Enable Early Identification and Prognosis Prediction for Symptomatic Aortic Stenosis and Heart Failure With Preserved Ejection Fraction After Transcatheter Aortic Valve Replacement: Multicenter Cohort Study<br>DOI: 10.2196/70587	Retrospective multicentre cohort	Population: n=326<br>Evaluation: NR / resampling-based or no distinct evaluation cohort explicitly adjudicated	NR / not explicitly resolved in the study-level table; retained in QA evidence	MACCE in AS+HFpEF<br>Horizon: Study-defined; see outcome definition	Mixed preprocedural + peri/postprocedural Echocardiography; Clinical/laboratory	Decision tree; k-nearest neighbours; random forest; support vector machine; XGBoost; LASSO variable selection	10-fold cross-validation<br>Performance: Reported; representative estimate retained in outcome-level analysis dataset/NR here<br>Audit/source-family: No specific overlap flag in current source-family audit
45	**SF-45** | 2025 — Patient risk evaluation for transcatheter aortic valve replacement (PRE-TAVR) — identification of real-time predictors of short- and long-term mortality<br>DOI: 10.1007/s00392-025-02704-6	Retrospective two-centre cohort	Population: n=2,256 overall; 1-year outcome cohort n=1,774; validation subset n=364<br>Evaluation: Primary mortality evaluation information base: n=364; events=NR; Internal validation cohort without EuroSCORE II; event count not separately reported	Overall 30-day deaths: 69/2,256 (3.1%); 1-year outcome analysed in n=1,774	30-day and 1-year mortality<br>Horizon: 30-day and 1-year mortality	Periprocedural CT/imaging; Echocardiography; ECG; Clinical/laboratory	LASSO regression / PRE-TAVR model	Evaluation strategy NR/unclear<br>Performance: validation cohort AUC 0.794 (95% CI 0.699–0.888)<br>Audit/source-family: No specific overlap flag in current source-family audit
46	**SF-46** | 2025 — Predictors of Length-of-Stay Among Transcatheter Aortic Valve Replacement Patients Using a Supervised Machine Learning Algorithm<br>DOI: 10.1016/j.jacadv.2025.101902	Multicentre registry/database cohort (21 centres)	Population: n=12,184 source cohort; n=9,172 analysed<br>Evaluation: NR / resampling-based or no distinct evaluation cohort explicitly adjudicated	NR / not explicitly resolved in the study-level table; retained in QA evidence	Short/long length of stay<br>Horizon: Study-defined; see outcome definition	Mixed preprocedural + peri/postprocedural Clinical/laboratory	Random forest; multivariable logistic-regression comparator	Cross-validation (fold scheme not resolved)<br>Performance: Reported; representative estimate retained in outcome-level analysis dataset/NR here<br>Audit/source-family: No specific overlap flag in current source-family audit
47	**SF-13** | 2025 — Preprocedural CT angiography and machine learning for mortality prediction after transcatheter aortic valve replacement<br>DOI: 10.1016/j.pcad.2025.04.007	Derivation cohort plus independent external sites; multicentre/multisite evaluation	Population: Derivation n=631; external validation n=596<br>Evaluation: Primary mortality evaluation information base: n=596; events=70; External multicentre validation cohort	Primary mortality evaluation: 70/596 (11.7%)	1-year all-cause mortality<br>Horizon: 1-year all-cause mortality	Mixed preprocedural + peri/postprocedural CT/imaging; Echocardiography; Clinical/laboratory	XGBoost	External/independent validation; hold-out<br>Performance: External validation AUC ~0.79<br>Audit/source-family: Kwiecinski/Warsaw mortality family: overlapping derivation era; one representative in primary mortality
48	**SF-48** | 2025 — Risk factors and predictive models for post-operative moderate-to-severe mitral regurgitation following transcatheter aortic valve replacement: a machine learning approach<br>DOI: 10.1186/s12872-025-04759-9	Retrospective	Population: n=987<br>Evaluation: NR / resampling-based or no distinct evaluation cohort explicitly adjudicated	NR / not explicitly resolved in the study-level table; retained in QA evidence	Moderate-to-severe post-TAVR MR<br>Horizon: Study-defined; see outcome definition	Mixed preprocedural + peri/postprocedural CT/imaging; Echocardiography; Clinical/laboratory	Logistic regression; Random forest	Held-out validation/test set<br>Performance: Reported; representative estimate retained in outcome-level analysis dataset/NR here<br>Audit/source-family: No specific overlap flag in current source-family audit
49	**SF-49** | 2025 — The Application of Multimodal Data Fusion Algorithm MULTINet in Postoperative Risk Assessment of TAVR<br>DOI: 10.3390/jcm14248620	Retrospective single-centre West China cohort	Population: n=773<br>Evaluation: Primary mortality evaluation information base: n=761; events=32; 5-fold OOF predictions, MIMIC-IV TAVR cohort	Primary mortality evaluation: 32/761 (4.2%)	Postoperative risk (MULTINet)<br>Horizon: Postoperative risk (MULTINet)	Mixed preprocedural + peri/postprocedural CT/imaging; ECG; Clinical/laboratory	MULTINet multimodal deep-learning model; XGBoost comparator	5-fold cross-validation<br>Performance: AUC 0.915<br>Audit/source-family: No specific overlap flag in current source-family audit
50	**SF-50** | 2025 — Using novel machine learning tools predict optimal discharge<br>DOI: 10.1016/j.acvd.2024.08.008	Retrospective nationwide administrative (NIS) cohort; multisite	Population: n=25,874 final analysed NIS admissions<br>Evaluation: NR / resampling-based or no distinct evaluation cohort explicitly adjudicated	NR / not explicitly resolved in the study-level table; retained in QA evidence	Optimal discharge / LOS<br>Horizon: Study-defined; see outcome definition	Mixed preprocedural + peri/postprocedural ECG; Clinical/laboratory; Administrative	Logistic regression; artificial neural network; XGBoost	holdout<br>Performance: Reported; representative estimate retained in outcome-level analysis dataset/NR here<br>Audit/source-family: No specific overlap flag in current source-family audit

ID	Source-family ID | Study / year / title / DOI	Design / setting	Population + evaluation information base	Event frequency	Outcome + horizon	Predictor timing + modality	Algorithm families	Validation / performance provenance / source-family note
51	**SF-51** | 2026 — A Clinical Risk Prediction Model for Identifying Patient Candidates for Same-day Discharge After Transcatheter Aortic Valve Replacement<br>DOI: 10.1016/j.jscai.2025.104110	Retrospective single-centre cohort with temporal/external testing within report	Population: Development n=730; temporal/external test n=158<br>Evaluation: n=158 temporal test	Composite adverse outcome: 91/730 (12.5%)	Same-day discharge candidacy/adverse-event composite<br>Horizon: Study-defined; see outcome definition	Mixed preprocedural + peri/postprocedural<br>CT/imaging; Clinical/laboratory	Random forest	External/independent validation; Cross-validation (fold scheme not resolved)<br>Performance: median AUC 0.76 (IQR 0.73–0.77)<br>Audit/source-family: No specific overlap flag in current source-family audit
52	**SF-52** | 2026 — A Transformer-Based Machine Learning Framework for Risk Stratification of Left Bundle Branch Block After Transcatheter Aortic Valve Replacement<br>DOI: 10.3390/diagnostics16101422	Retrospective multicentre cohort (3 institutions)	Population: n=242<br>Evaluation: NR / resampling-based or no distinct evaluation cohort explicitly adjudicated	NR / not explicitly resolved in the study-level table; retained in QA evidence	New-onset persistent LBBB<br>Horizon: Study-defined; see outcome definition	Mixed preprocedural + peri/postprocedural<br>CT/imaging; Echocardiography; ECG; Clinical/laboratory	Transformer-based feature selection + gradient boosting classifier	bootstrap<br>Performance: AUC 0.61<br>Audit/source-family: No specific overlap flag in current source-family audit
53	**SF-53** | 2026 — Clinical significance of machine learning algorithm in predicting PPM during TAVR in small annuli<br>DOI: 10.1007/s12928-025-01215-5	Retrospective derivation cohort with external validation from other centres	Population: Derivation n=273; external validation n=118<br>Evaluation: n=118 external	NR / not explicitly resolved in the study-level table; retained in QA evidence	Prosthesis–patient mismatch / PGAV measurement<br>Horizon: Study-defined; see outcome definition	Mixed preprocedural + peri/postprocedural<br>CT/imaging; Echocardiography; Fluoroscopy/procedural imaging; Clinical/laboratory	XGBoost	External/independent validation<br>Performance: Reported; representative estimate retained in outcome-level analysis dataset/NR here<br>Audit/source-family: No specific overlap flag in current source-family audit<br>Terminology lock: PPM = prosthesis–patient mismatch, not pacemaker implantation.
54	**SF-54** | 2026 — Construction and validation of risk prediction models for new- onset conduction block complications after transcatheter aortic valve replacement<br>DOI: 10.1186/s12872-025-05397-x	Retrospective single-centre cohort	Population: n=163 analysed<br>Evaluation: NR / resampling-based or no distinct evaluation cohort explicitly adjudicated	NR / not explicitly resolved in the study-level table; retained in QA evidence	New-onset conduction block<br>Horizon: Study-defined; see outcome definition	Mixed preprocedural + peri/postprocedural<br>CT/imaging; Echocardiography; ECG; Clinical/laboratory	LASSO variable selection + multivariable logistic regression / nomogram	bootstrap<br>Performance: Reported; representative estimate retained in outcome-level analysis dataset/NR here<br>Audit/source-family: No specific overlap flag in current source-family audit
55	**SF-55** | 2026 — Development and Validation of a Prognostic Nomogram for Post–Transcatheter Aortic Valve Replacement Heart Failure Hospitalization in Patients With Concurrent Symptomatic Aortic Stenosis and Heart Failure With Preserved Ejection Fraction: A Multicenter Study<br>DOI: 10.1161/JAHA.125.046606	Retrospective multicentre cohort	Population: Derivation n=191; external validation n=130<br>Evaluation: n=130 external	NR / not explicitly resolved in the study-level table; retained in QA evidence	Heart-failure hospitalization<br>Horizon: Study-defined; see outcome definition	Mixed preprocedural + peri/postprocedural<br>CT/imaging; Echocardiography; Clinical/laboratory	Random forest; XGBoost; SuperPC; plsRcox; LASSO-Cox; GBM; CoxBoost; Cox regression/nomogram	External/independent validation; bootstrap<br>Performance: Reported; representative estimate retained in outcome-level analysis dataset/NR here<br>Audit/source-family: No specific overlap flag in current source-family audit
56	**SF-56** | 2026 — Development of a multi-label deep neural network model for predicting immediate paravalvular leakage and new-onset conduction disturbances after transcatheter aortic valve replacement: A retrospective cohort study<br>DOI: 10.1177/20552076261427502	Retrospective single-centre West China cohort	Population: n=966<br>Evaluation: NR / resampling-based or no distinct evaluation cohort explicitly adjudicated	NR / not explicitly resolved in the study-level table; retained in QA evidence	Immediate PVL + new conduction disturbance<br>Horizon: Immediate PVL + new conduction disturbance	Mixed preprocedural + peri/postprocedural<br>CT/imaging; Echocardiography; ECG; Clinical/laboratory	Multi-label deep neural network (Muex optimization) + neural/traditional multilabel comparators	bootstrap<br>Performance: Reported; representative estimate retained in outcome-level analysis dataset/NR here<br>Audit/source-family: No specific overlap flag in current source-family audit
57	**SF-57** | 2026 — Explainable machine learning for risk stratification following TAVI<br>DOI: 10.1177/20552076261466380	Retrospective single-centre Qatar registry cohort	Population: n=256<br>Evaluation: NR / resampling-based or no distinct evaluation cohort explicitly adjudicated	NR / not explicitly resolved in the study-level table; retained in QA evidence	Multiple post-TAVI outcomes<br>Horizon: Study-defined; see outcome definition	Postprocedural predictors included/unclear index time<br>Echocardiography; ECG; Clinical/laboratory	Support vector machine; k-nearest neighbours; artificial neural network	train/test<br>Performance: Reported; representative estimate retained in outcome-level analysis dataset/NR here<br>Audit/source-family: No specific overlap flag in current source-family audit
58	**SF-58** | 2026 — Generalisable artificial intelligence ECG trained on public data for outcome prediction after transcatheter aortic valve replacement<br>DOI: 10.1136/heartjnl-2025-327462	Prospective single-centre TAVR registry external AI-ECG evaluation cohort	Population: TAVR evaluation cohort n=439 for pretrained AI-ECG<br>Evaluation: Primary mortality evaluation information base: n=439; events=NR; External TAVR cohort; 1-year mortality rate 13.0%	30-day mortality 2.7%; 1-year mortality 13.0% | Primary mortality evaluation event prevalence: 13.0%	30-day and 1-year all-cause mortality<br>Horizon: 30-day and 1-year all-cause mortality	Mixed preprocedural + peri/postprocedural<br>CT/imaging; ECG; Clinical/laboratory	Transformer-based AI-ECG deep-learning model	External/independent validation; bootstrap<br>Performance: AUC 0.85 (30 d)<br>Audit/source-family: No specific overlap flag in current source-family audit
59	**SF-59** | 2026 — Identification of patients at risk for adverse events and poor symptom improvement after transcatheter aortic	Retrospective derivation cohort with external prospective Bern-registry validation	Population: Derivation/eligible n=827 (primary outcome analysis	Adverse-event + poor symptom-improvement	Adverse-event + poor symptom-improvement composite	Mixed preprocedural + peri/postprocedural	LASSO logistic regression	External/independent validation; bootstrap<br>Performance: Reported;

ID	Source-family ID | Study / year / title / DOI	Design / setting	Population + evaluation information base	Event frequency	Outcome + horizon	Predictor timing + modality	Algorithm families	Validation / performance provenance / source-family note
	valve implantation<br>DOI: 10.1016/j.ahjo.2026.100735		n=630); external validation n=3,963<br>Evaluation: n=3,963 external	composite: 101/827 (12%)	Horizon: Study-defined; see outcome definition	Echocardiography; Clinical/laboratory		representative estimate retained in outcome-level analysis dataset/NR here<br>Audit/source-family: No specific overlap flag in current source-family audit
60	**SF-60** | 2026 — Machine Learning-Driven Probability of Permanent Pacemaker Implantation After Transcatheter Aortic Valve Replacement<br>DOI: 10.3390/diagnostics16111720	Prospective single-centre analytic cohort	Population: n=179; PPI events 17 (9.5%) within 30 days<br>Evaluation: NR / resampling-based or no distinct evaluation cohort explicitly adjudicated	PPI within 30 days: 17/179 (9.5%)	Permanent pacemaker implantation<br>Horizon: Study-defined; see outcome definition	Mixed preprocedural + peri/postprocedural CT/imaging; Echocardiography; ECG; Clinical/laboratory	Class-weighted logistic regression; RBF-SVM; random forest; Extra Trees; Balanced Random Forest; multilayer perceptron	3-fold cross-validation<br>Performance: Reported; representative estimate retained in outcome-level analysis dataset/NR here<br>Audit/source-family: No specific overlap flag in current source-family audit
61	**SF-61*** | 2026 — Neural Network-Based Prediction of Residual Paravalvular Leak in Bicuspid Aortic Valve TAVI Using CT-Derived Anatomical Features<br>DOI: 10.3390/biomedicines14040946	Retrospective single-centre observational cohort	Population: n=402; residual ≥moderate PVL events 36 (9.0%)<br>Evaluation: Whole report cohort n=402/36 events; retained model AUC evaluated in Stage-2 information base n=210 with 35 events using 5-fold out-of-fold CV	Whole cohort: 36/402 (9.0%). Stage-2 OOF evaluation base for retained AUC: 35/210 (16.7%).	Residual ≥moderate PVL in BAV TAVI<br>Horizon: Study-defined; see outcome definition	Mixed preprocedural + peri/postprocedural CT/imaging; Echocardiography; ECG; Clinical/laboratory	Multimodal deep neural network (3D ResNet + MLP)	5-fold stratified CV.<br>Performance: mean fold AUC 0.822 ± 0.114; the paper's 0.680–0.964 interval is fold-derived and was not used as a sampling CI.<br>Meta-analysis precision: Hanley–McNeil/delta reconstruction from Stage-2 OOF n=210/35, 95% CI 0.717–0.894. Source-family: BAV/PVL ecosystem; overlap possible; independence audited.
62	**SF-62** | 2025 — Parsimonious machine learning model to predict 1-year mortality and procedural futility after transcatheter aortic valve replacement<br>DOI: 10.1136/heartjnl-2025-325928	Retrospective derivation at KCH with independent GSTT validation	Population: Discovery n=1,025; independent external validation n=270<br>Evaluation: Primary mortality evaluation information base: n=270; events=NR; Independent external cohort; ~15% 1-year mortality	1-year deaths in discovery cohort: 158/1,025 (15%); external cohort events reported separately | Primary mortality evaluation event prevalence: 15.0%	1-year all-cause mortality<br>Horizon: 1-year all-cause mortality	Mixed preprocedural + peri/postprocedural Echocardiography; Clinical/laboratory	Support vector machine; random forest; elastic-net logistic regression	External/independent validation; bootstrapping<br>Performance: external AUC 0.84 | discovery AUC 0.81<br>Audit/source-family: No specific overlap flag in current source-family audit<br>Audit lock: abstract/results support external AUC 0.84; Figure 3 caption transposition tested separately.
63	**SF-63*** | 2026 — Periprocedural evaluation of patients with BAV stenosis undergoing TAVR: a machine learning-based study<br>DOI: 10.1136/openhrt-2025-003749	Retrospective multicentre cohort	Population: n=1,427 screened; derivation n=1,129; external validation n=137; analysed total n=1,266<br>Evaluation: n=137 external	Derivation training set: 113/1,016 events (11.1%); internal test: 13/113 (11.5%); external cohort reported separately	Periprocedural adverse-event composite in BAV<br>Horizon: Periprocedural adverse-event composite in BAV	Mixed preprocedural + peri/postprocedural CT/imaging; Echocardiography; ECG; Clinical/laboratory	Logistic regression; random forest; XGBoost; LightGBM; additional benchmark classifiers	External/independent validation<br>Performance: Reported; representative estimate retained in outcome-level analysis dataset/NR here<br>Audit/source-family: BAV/PVL ecosystem: institutions/authors overlap possible; endpoint- and cohort-specific independence audit required
64	**SF-64** | 2026 — Predictive Value of Machine Learning–Based Echocardiographic Myocardial Texture After Transcatheter Aortic Valve Replacement<br>DOI: 10.1111/echo.70439	Retrospective two-centre cohort (authors explicitly describe it as two-centre, not multicentre)	Population: n=121 analysed<br>Evaluation: NR / resampling-based or no distinct evaluation cohort explicitly adjudicated	NR / not explicitly resolved in the study-level table; retained in QA evidence	1-year all-cause mortality + HF-event composite<br>Horizon: 1-year all-cause mortality + HF-event composite	Mixed preprocedural + peri/postprocedural Echocardiography; ECG; Clinical/laboratory	Random forest; AdaBoost; Extra Trees; decision tree; SVM; k-nearest neighbours; ANN; XGBoost	External/independent validation; 10-fold cross-validation<br>Performance: Reported; representative estimate retained in outcome-level analysis dataset/NR here<br>Audit/source-family: No specific overlap flag in current source-family audit
65	**SF-65*** | 2026 — Predictors of Paravalvular Leakage After Transcatheter Aortic Valve Replacement in Patients With BAV<br>DOI: 10.1016/j.jacadv.2025.102566	Retrospective national multicentre cohort (8 centres)	Population: n=1,370 screened; derivation n=1,080; independent external cohort reported (eligible subset n=109)<br>Evaluation: n=109 eligible external subset	NR / not explicitly resolved in the study-level table; retained in QA evidence	PVL in BAV TAVR<br>Horizon: Study-defined; see outcome definition	Mixed preprocedural + peri/postprocedural CT/imaging; Echocardiography; Clinical/laboratory	Random forest; logistic-regression comparator	External/independent validation<br>Performance: Reported; representative estimate retained in outcome-level analysis dataset/NR here<br>Audit/source-family: BAV/PVL ecosystem: institutions/authors overlap possible; endpoint- and cohort-specific independence audit required
66	**SF-66** | 2026 — Using AI-ECG to Stratify Long-Term Mortality Risk and Prognosis in TAVR Patients<br>DOI: 10.1016/j.jacasi.2026.05.034	Dual-centre retrospective cohort	Population: n=711<br>Evaluation: Primary mortality evaluation information base: n=251; events=59; Independent external cohort; 59 3-year	3-year mortality: high-risk stratum 42.8%–74.1% vs low-risk 35/212 (16.5%) | Primary mortality evaluation: 59/251 (23.5%)	3-year all-cause mortality<br>Horizon: 3-year all-cause mortality	Mixed preprocedural + peri/postprocedural CT/imaging; Echocardiography; ECG; Fluoroscopy/procedural imaging; Clinical/laboratory	CNN-based AI-ECG model; Cox-regression clinical comparator	External/independent validation; bootstrap<br>Performance: external validation AUC 0.712 (0.627–0.795)<br>Audit/source-family: No specific overlap flag in current source-family audit

ID	Source-family ID | Study / year / title / DOI	Design / setting	Population + evaluation information base	Event frequency	Outcome + horizon	Predictor timing + modality	Algorithm families	Validation / performance provenance / source-family note
			deaths from reported risk groups					
67	**SF-67** | 2026 — eCAPRI: a novel tool combining clinical and imaging data for post-TAVI mortality prediction<br>DOI: 10.1007/s00330-025-12184-x	Retrospective TAVI-Lyon clinical outcome cohort; separate segmentation data from 4 hospitals	Population: TAVI-Lyon retrospective cohort n=957; CalciumEval segmentation/evaluation set n=1,111; mortality-score evaluation uses the prespecified held-out subset<br>Evaluation: Primary mortality evaluation information base: n=192; events=18; Held-out evaluation set: 174 no-event / 18 event	TAVI-Lyon cohort: 81/957 deaths (9.3%) over reported follow-up; 1-year score endpoint extracted separately in model evaluation | Primary mortality evaluation: 18/192 (9.4%)	1-year all-cause mortality<br>Horizon: 1-year all-cause mortality	Mixed preprocedural + peri/postprocedural<br>CT/imaging; Clinical/laboratory	Logistic regression; multilayer perceptron; gradient boosting; Cox regression; deep-learning CT segmentation component	bootstrap<br>Performance: AUC 0.731<br>Audit/source-family: No specific overlap flag in current source-family audit

Source-family ID and representative-effect note. Shared IDs identify reports judged to reuse or substantially overlap the same underlying patient source and therefore not treated as independent for the same endpoint. SF-01 (reports 1/14/18) is the Amsterdam/Catharina inter-hospital family: report 14 (Mamprin et al., 2021) supplies the representative mortality effect in the locked primary mortality synthesis; report 1 (Lopes et al., 2019) is an alternate family representative examined in the source-family sensitivity analysis, and report 18 is retained as a linked distributed-learning report but is not counted as independent same-endpoint evidence. SF-12 (reports 12/42) is the Christ Hospital PPMI/PPI family: report 12 (Truong et al., 2021) supplies the strict-PPMI effect shown in Supplementary Figure S9; report 42 is a later federated/platform reanalysis of the same clinical data ecosystem and is not counted independently for PPMI. SF-13 (reports 13/47) is the Kwiecinski/Warsaw mortality family: report 47 (Kwiecinski et al., 2025) supplies the representative mortality effect in the locked primary mortality dataset; the linked 2023 report is retained as an alternate family representative and the family-representative choice is stress-tested in Supplementary Table S9. An asterisk (SF-61, SF-63*, SF-65*) flags the potential BAV/PVL overlap ecosystem. These reports were not forcibly collapsed: SF-61 is a West China single-centre CT-derived residual-PVL cohort, whereas SF-63 and SF-65 are multicentre BAV studies with overlapping investigators but different stated analytic cohorts and targets; because explicit patient-level reuse could not be established from the published reports, they remain separate source families and are visibly flagged as potential overlap. Linkage decisions used explicit cohort reuse or a convergence of registry/database or institution, recruitment period, eligibility criteria, cohort/event counts, outcome/horizon, and derivation/validation descriptions; author or institutional overlap alone was insufficient.*

Supplementary Table S3. Full-text methodological, performance-reporting, and clinical-readiness extraction across the 67 included reports

ID	External/temporal validation	Internal CV / resampling	Calibration / Brier	DCA / net benefit	AUPRC / PR-AUC	Explainability	Prospective-data status	Multicentre / multisite status	Missing-data handling	Feature selection / dimensionality reduction	Hyperparameter tuning	Class-imbalance handling
1	No independent external/temporal evaluation identified	5-fold cross-validation	Not reported	Not reported	Not reported	Feature importance	NR/unclear	NR/unclear	Complete-case/exclusion	NR/none explicit	Grid search; Bayesian optimization	Stratified sampling
2	No independent external/temporal evaluation identified	Not reported/NA	Not reported	Not reported	Not reported	Not reported	NR/unclear	Yes — multicentre	Not reported	NR/none explicit	Not reported/NA	Not reported
3	Yes — stronger external/temporal/geographic/inter-centre evaluation	10-fold crossvalidation	Calibration plot/curve; Brier score; Brier detail: Brier Score (BS), which is related to calibration, in terms of accuracy of the predicted probabilitie	Not reported	Not reported	Not reported	Yes	Yes — national multisite	Multiple imputation; Iterative/model-based imputation	NR/none explicit	Hyperparameter tuning reported; search strategy NR	Not reported
4	No independent external/temporal evaluation identified	5-fold cross-validation	Not reported	Not reported	Not reported	Not reported	No — retrospective	Yes — multicentre (3 Mayo sites)	Not reported	NR/none explicit	Hyperparameter tuning reported; search strategy NR	Not reported
5	No independent external/temporal evaluation identified	Not reported/NA	Not reported	Not reported	Not reported	Feature importance	NR/unclear	NR/unclear	Handling mentioned; method NR	NR/none explicit	Not reported/NA	SMOTE; Oversampling; Undersampling
6	No independent external/temporal evaluation identified	10-fold cross-validation	Not reported	Not reported	Not reported	Not reported	Partial — retrospective analysis of prospectively enrolled registry participants	No — single-centre	Mean imputation	NR/none explicit	Hyperparameter tuning reported; search strategy NR	Stratified sampling
7	No independent external/temporal evaluation identified	Reported; fold scheme NR	Not reported	Not reported	Not reported	SHAP; Feature importance	No — retrospective	No — single-centre	Not reported	LASSO; RFE; Boruta	Hyperparameter tuning reported; search strategy NR	Not reported
8	No independent external/temporal evaluation identified	Reported; fold scheme NR	Not reported	Not reported	Not reported	Feature importance	No — retrospective	Yes — nationwide multisite administrative data	Not reported	LASSO; Feature-importance selection	Not reported/NA	Oversampling; Undersampling
9	Yes — stronger external/temporal/geographic/inter-centre evaluation	10-fold cross-validation	Calibration plot/curve; Calibration slope/intercept	Not reported	area under the precision-recall curve (AUC-PR), and the F1 score	Not reported	No — retrospective	Yes	Multiple imputation	LASSO	Hyperparameter tuning reported; search strategy NR	Not reported
10	No independent external/temporal evaluation identified	Not reported/NA	Not reported	Not reported	Not reported	Feature importance	No — retrospective	No — single-centre	Handling mentioned; method NR	NR/none explicit	Bayesian optimization; Hyperopt	Oversampling
11	No independent external/temporal evaluation identified	Not reported/NA	Not reported	Not reported	Not reported	Feature importance	No — retrospective	Yes — nationwide multisite administrative data	Handling mentioned; method NR	Feature-importance selection	Not reported/NA	Oversampling
12	No independent external/temporal evaluation identified	Not reported/NA	Brier score; Brier detail: Brier scores, and the area under the curve (AUC)	Not reported	Not reported	Feature importance	No — retrospective	NR/unclear	Multiple imputation	NR/none explicit	Not reported/NA	SMOTE; Oversampling
13	Yes — stronger external/temporal/geographic/inter-centre evaluation	Not reported/NA	Calibration plot/curve	Not reported	Not reported	Feature importance	NR/unclear	Yes	Not reported	NR/none explicit	Hyperparameter tuning reported; search strategy NR	Not reported
14	Yes — stronger external/temporal/geographic/inter-centre evaluation	Reported; fold scheme NR	Calibration plot/curve; Brier score; Brier detail: Brier Score loss of each classifier involved in the experiments	Not reported	Not reported	Not reported	NR/unclear	Yes	Multiple imputation; Mean imputation	NR/none explicit	Hyperparameter tuning reported; search strategy NR	SMOTE; Oversampling; Class weighting
15	No independent external/temporal evaluation identified	nested cross-validation	Not reported	Not reported	Precision-Recall Curves	SHAP; Feature importance	Partial — retrospective cohort; CT measurements prospectively acquired/blinded	No — single-centre	Handling mentioned; method NR	RFE; Feature-importance selection	Nested tuning	SMOTE; Oversampling

ID	External/temporal validation	Internal CV / resampling	Calibration / Brier	DCA / net benefit	AUPRC / PR-AUC	Explainability	Prospective-data status	Multicentre / multisite status	Missing-data handling	Feature selection / dimensionality reduction	Hyperparameter tuning	Class-imbalance handling
16	No independent external/temporal evaluation identified	nested cross-validation	Not reported	Not reported	Not reported	Feature importance	No — retrospective	NR/unclear	Mean imputation	Feature-importance selection	Grid search; Nested tuning	Class weighting
17	No independent external/temporal evaluation identified	10-fold cross-validation	Not reported	Not reported	Not reported	Not reported	No — retrospective	NR/unclear	Complete-case/exclusion	NR/none explicit	Not reported/NA	Not reported
18	No independent external/temporal evaluation identified	5-fold cross-validation; 20-fold crossvalidation	Not reported	Not reported	Not reported	Not reported	Partial — analysis of prospectively collected registry data	Yes — two-centre/multisite	Multiple imputation; Mean imputation	NR/none explicit	Grid search	Oversampling; Class weighting
19	No independent external/temporal evaluation identified	5-fold cross-validation	Not reported	Not reported	area under the precision-recall curve (AUC-PR)	SHAP	No — retrospective	NR/unclear	Not reported	NR/none explicit	Grid search	Handling reported; method NR
20	No independent external/temporal evaluation identified	Reported; fold scheme NR	Not reported	Not reported	Not reported	Not reported	No — retrospective	No — single-centre	Not reported	NR/none explicit	Grid search	Not reported
21	No independent external/temporal evaluation identified	Reported; fold scheme NR	Not reported	Not reported	Not reported	Not reported	No — retrospective	Yes — multicentre	Not reported	NR/none explicit	Not reported/NA	Not reported
22	No independent external/temporal evaluation identified	5-fold cross validation	Calibration plot/curve	Not reported	Not reported	Not reported	Partial — retrospective primary analysis; prospective external SwissTAVI validation	Yes — multisite primary/external registry data	Multiple imputation; Missingness-indicator/explicit handling	Feature-importance selection	Grid search	Not reported
23	No independent external/temporal evaluation identified	Not reported/NA	Not reported	Not reported	Not reported	Not reported	No — retrospective	Yes — nationwide multisite administrative data	Handling mentioned; method NR	NR/none explicit	Hyperparameter tuning reported; search strategy NR	Not reported
24	No independent external/temporal evaluation identified	Not reported/NA	Not reported	Not reported	Not reported	Not reported	Yes	NR/unclear	Multiple imputation	RFE; Univariate screening	Not reported/NA	Not reported
25	No independent external/temporal evaluation identified	10-fold cross-validation; leave-one-out crossvalidation	Calibration plot/curve	Not reported	Not reported	SHAP	No — retrospective	NR/unclear	Handling mentioned; method NR	Feature-importance selection	Hyperparameter tuning reported; search strategy NR	Oversampling; Class weighting
26	No independent external/temporal evaluation identified	leave-one-out cross validation; LOO-CV	Not reported	Not reported	Not reported	Not reported	NR/unclear	NR/unclear	Not reported	Feature-importance selection	Not reported/NA	Not reported
27	No independent external/temporal evaluation identified	5-fold cross-validation; 5-fold cross validation	Not reported	Not reported	Not reported	Not reported	No — retrospective	Yes — multicentre (3 sites)	Handling mentioned; method NR	NR/none explicit	Hyperparameter tuning reported; search strategy NR	Not reported
28	No independent external/temporal evaluation identified	10-fold cross-validation	Not reported	Not reported	Not reported	Not reported	Partial — retrospective analysis of prospectively collected institutional data	No — single-centre	Handling mentioned; method NR	Feature-importance selection	Not reported/NA	Not reported
29	No independent external/temporal evaluation identified	Not reported/NA	Not reported	Not reported	Not reported	Feature importance	NR/unclear	No — single-centre	Multiple imputation	Univariate screening; Feature-importance selection	Not reported/NA	Not reported
30	Yes — stronger external/temporal/geographic/inter-centre evaluation	Not reported/NA	Not reported	Not reported	Not reported	Not reported	Partial — retrospective derivation; prospective temporal validation cohort	No — single-centre	Not reported	NR/none explicit	Not reported/NA	Not reported
31	Yes — stronger external/temporal/geographic/inter-centre evaluation	Reported; fold scheme NR	Calibration plot/curve	decision curve analysis, comparing the net benefit of each score across different bleeding-risk thresholds (expressed as 30	Not reported	Not reported	Yes — prospective registries	Yes — multicentre	Multiple imputation	RFE; Univariate screening	Not reported/NA	Not reported
32	Yes — stronger external/temporal/geographic/inter-centre evaluation	Not reported/NA	Not reported	Not reported	Not reported	SHAP	No — retrospective	Yes — nationwide multisite administrative data	Not reported	LASSO; PCA/dimensionality reduction	Not reported/NA	Not reported

ID	External/temporal validation	Internal CV / resampling	Calibration / Brier	DCA / net benefit	AUPRC / PR-AUC	Explainability	Prospective-data status	Multicentre / multisite status	Missing-data handling	Feature selection / dimensionality reduction	Hyperparameter tuning	Class-imbalance handling
33	No independent external/temporal evaluation identified	Reported; fold scheme NR	Calibration plot/curve; Hosmer-Lemeshow	Not reported	Not reported	Not reported	NR/unclear	NR/unclear — integrated health-system cohort; centre count not explicitly designated	Not reported	NR/none explicit	Hyperparameter tuning reported; search strategy NR	Not reported
34	No independent external/temporal evaluation identified	Reported; fold scheme NR	Not reported	Not reported	Not reported	Not reported	NR/unclear	NR/unclear	Handling mentioned; method NR	NR/none explicit	Not reported/NA	Handling reported; method NR
35	No independent external/temporal evaluation identified	Reported; fold scheme NR	Not reported	Not reported	Not reported	Not reported	Partial — retrospective analysis of prospectively designed registry	No — single-centre	Handling mentioned; method NR	NR/none explicit	Hyperparameter tuning reported; search strategy NR	Oversampling
36	No independent external/temporal evaluation identified	Reported; fold scheme NR	Not reported	Not reported	Not reported	SHAP; Feature importance	No — retrospective	Yes — 21 hospitals	Median imputation	RFE	Grid search	Not reported
37	No independent external/temporal evaluation identified	Not reported/NA	Calibration plot/curve	Not reported	Not reported	SHAP	No — retrospective	Yes — multicentre	Not reported	NR/none explicit	Not reported/NA	Not reported
38	No independent external/temporal evaluation identified	Reported; fold scheme NR	Not reported	Not reported	Not reported	Not reported	No/unclear — no prospective clinical evaluation identified	NR/unclear — no explicit multicentre outcome evaluation identified	Handling mentioned; method NR	NR/none explicit	Grid search	SMOTE
39	No independent external/temporal evaluation identified	10-fold cross validation	Not reported	Not reported	Not reported	SHAP	No — retrospective	No — single-centre	Not reported	Feature-importance selection	Hyperparameter tuning reported; search strategy NR	SMOTE; ADASYN; Oversampling; Class weighting; Stratified sampling
40	No independent external/temporal evaluation identified	10-fold cross-validation; 3-fold cross-validation	Not reported	Not reported	Not reported	SHAP; Feature importance	NR/unclear	No — single-centre	Handling mentioned; method NR	LASSO; Feature-importance selection	Grid search	Not reported
41	No independent external/temporal evaluation identified	10-fold cross-validation	Not reported	Not reported	Not reported	Not reported	No — retrospective	No — single-centre	Not reported	LASSO; Univariate screening; Feature-importance selection	Not reported/NA	Not reported
42	No independent external/temporal evaluation identified	Not reported/NA	Not reported	Not reported	Not reported	SHAP	NR/unclear	NR/unclear	Handling mentioned; method NR	NR/none explicit	Hyperparameter tuning reported; search strategy NR	SMOTE; Oversampling
43	No independent external/temporal evaluation identified	10-fold cross-validation	Not reported	Not reported	Not reported	SHAP	Yes — prospective registry cohort	No — single-centre clinical outcome cohort	Median imputation; Mean imputation	LASSO; RFE; Feature-importance selection	Hyperparameter tuning reported; search strategy NR	SMOTE; Oversampling; Stratified sampling
44	No independent external/temporal evaluation identified	10-fold cross-validation	Calibration plot/curve; Brier score; Brier detail: Brier score	Decision-curve/net-benefit analysis reported	area under the precision-recall curve, specificity, sensitivity, F1-score, recall, negative predictive value, positive predictive value,	SHAP; Feature importance	No — retrospective	Yes — multicentre	Not reported	LASSO; Boruta; Feature-importance selection	Not reported/NA	Not reported
45	No independent external/temporal evaluation identified	Not reported/NA	Not reported	Not reported	Not reported	Not reported	No — retrospective	Yes — two-centre	Multiple imputation; Complete-case/exclusion	LASSO	Not reported/NA	Not reported
46	No independent external/temporal evaluation identified	Reported; fold scheme NR	Not reported	Not reported	Not reported	SHAP	No — retrospective	Yes — 21 centres	Median imputation	RFE	Not reported/NA	Not reported
47	Yes — stronger external/temporal/geographic/inter-centre evaluation	10-fold crossvalidation	Not reported	Not reported	Not reported	SHAP; Feature importance	NR/unclear	Yes — multicentre/multisite	Not reported	Feature-importance selection	Not reported/NA	Not reported
48	No independent external/temporal evaluation identified	Not reported/NA	Not reported	Not reported	Not reported	SHAP; Feature importance	No — retrospective	NR/unclear	Median imputation	Univariate screening	Not reported/NA	Not reported
49	No independent external/temporal evaluation identified	5-fold cross-validation	Calibration plot/curve; Brier score; Brier detail: Brier scores, and its	decision curve analysis (DCA)	area under the precision-recall curve (AUPR) and the recall rate	Feature importance	No — retrospective	No — single-centre	Multiple imputation; Complete-case/exclusion	NR/none explicit	Hyperparameter tuning reported; search strategy NR	Class weighting

ID	External/temporal validation	Internal CV / resampling	Calibration / Brier	DCA / net benefit	AUPRC / PR-AUC	Explainability	Prospective-data status	Multicentre / multisite status	Missing-data handling	Feature selection / dimensionality reduction	Hyperparameter tuning	Class-imbalance handling
			clinical practicability was assessed through decision curve analysis (DCA)		were used for prediction evaluation							
50	No independent external/temporal evaluation identified	Reported; fold scheme NR	Not reported	Not reported	Not reported	Feature importance	No — retrospective	Yes — nationwide multisite administrative data	Not reported	NR/none explicit	Not reported/NA	Not reported
51	Yes — stronger external/temporal/geographic/inter-centre evaluation	Reported; fold scheme NR	Calibration plot/curve; Calibration slope/intercept	Not reported	Not reported	SHAP	No — retrospective	No — single-centre	Complete-case/exclusion	NR/none explicit	Not reported/NA	Not reported
52	No independent external/temporal evaluation identified	5-fold cross-validation	Calibration plot/curve	Not reported	Not reported	SHAP; Feature importance	No — retrospective	Yes — 3 institutions	Not reported	Feature-importance selection	Hyperparameter tuning reported; search strategy NR	Handling reported; method NR
53	Yes — stronger external/temporal/geographic/inter-centre evaluation	Not reported/NA	Not reported	Not reported	Not reported	SHAP; Feature importance	No — retrospective	Yes — multisite derivation/external validation	Not reported	PCA/dimensionality reduction	Not reported/NA	Not reported
54	No independent external/temporal evaluation identified	Reported; fold scheme NR	Calibration plot/curve	decision curve analysis	Not reported	Not reported	No — retrospective	No — single-centre	Not reported	LASSO	Not reported/NA	Not reported
55	Yes — stronger external/temporal/geographic/inter-centre evaluation	10-fold cross-validation	Calibration plot/curve	decision curve analysis at the 12-and 20-month marks, assessing the net benefit across clinically relevant threshold probab	Not reported	Not reported	No — retrospective	Yes — multicentre	Not reported	LASSO; Boruta; Feature-importance selection	Hyperparameter tuning reported; search strategy NR	Not reported
56	No independent external/temporal evaluation identified	Not reported/NA	Calibration plot/curve	Not reported	Not reported	SHAP; Feature importance	No — retrospective	No — single-centre	Handling mentioned; method NR	Feature-importance selection	Hyperparameter tuning reported; search strategy NR	SMOTE; Oversampling; Class weighting
57	No independent external/temporal evaluation identified	5-fold cross validation	Not reported	Not reported	Not reported	SHAP; Feature importance	No — retrospective	No — single-centre	Multiple imputation	Feature-importance selection	Bayesian optimization	SMOTE; Oversampling
58	Yes — stronger external/temporal/geographic/inter-centre evaluation	Not reported/NA	Not reported	Not reported	precision-recall curve (PRC)	Not reported	Yes — prospective registry cohort	No — single-centre	Complete-case/exclusion	NR/none explicit	Hyperparameter tuning reported; search strategy NR	Not reported
59	Yes — stronger external/temporal/geographic/inter-centre evaluation	Not reported/NA	Not reported	Not reported	Not reported	Not reported	Partial — retrospective derivation; prospective external registry validation	Yes — multisite validation	Handling mentioned; method NR	LASSO	Not reported/NA	Not reported
60	No independent external/temporal evaluation identified	3-fold cross-validation	Brier score; Brier detail: Brier score was worse than the prevalence-only baseline, suggesting suboptimal calibration of the pre	Not reported	precision-recall area under the curve (PR-AUC)	SHAP; Permutation importance; Feature importance	Yes — prospective analytic cohort	No — single-centre	Not reported	NR/none explicit	Hyperparameter tuning reported; search strategy NR	SMOTE; Oversampling; Class weighting
61	No independent external/temporal evaluation identified	5-fold cross-validation	Calibration plot/curve; Brier score; Brier detail: Brier score, computed across all out-of-fold predictions from the 5-fold cross-validation	decision curve analysis (DCA)	Not reported	Attention/heatmap	No — retrospective	No — single-centre	Not reported	NR/none explicit	Grid search	Stratified sampling
62	Yes — stronger external/temporal/geographic/inter-centre evaluation	Not reported/NA	Calibration plot/curve; Calibration slope/intercept	Not reported	Not reported	Not reported	No — retrospective	Yes — multisite derivation/external validation	Handling mentioned; method NR	Feature-importance selection	Hyperparameter tuning reported; search strategy NR	Handling reported; method NR
63	Yes — stronger external/temporal/geographic/inter-centre evaluation	Not reported/NA	Not reported	decision curve analysis was conducted to	Not reported	Not reported	No — retrospective	Yes — multicentre	Not reported	LASSO	Not reported/NA	Not reported

ID	External/temporal validation	Internal CV / resampling	Calibration / Brier	DCA / net benefit	AUPRC / PR-AUC	Explainability	Prospective-data status	Multicentre / multisite status	Missing-data handling	Feature selection / dimensionality reduction	Hyperparameter tuning	Class-imbalance handling
				determine the applicability of this risk score in clinical practice								
64	Yes — stronger external/temporal/geographic/inter-centre evaluation	10-fold cross-validation	Calibration plot/curve	decision curve analysis	Not reported	SHAP	No — retrospective	Yes — two-centre/multisite (not termed multicentre by authors)	Not reported	LASSO; Feature-importance selection	Grid search	Not reported
65	Yes — stronger external/temporal/geographic/inter-centre evaluation	Not reported/NA	Not reported	decision curve analysis and clinical impact curve analysis verified the clinical applicability and net benefit	Not reported	Not reported	No — retrospective	Yes — 8 centres	Not reported	Univariate screening	Not reported/NA	Not reported
66	Yes — stronger external/temporal/geographic/inter-centre evaluation	Reported; fold scheme NR	Calibration plot/curve; Brier score reported	Decision-curve analysis confirmed clinical net benefit across the evaluated threshold-probability range	Not reported	Attention/heatmap	No — retrospective	Yes — dual-centre	Mean imputation	Univariate screening	Not reported/NA	Not reported
67	No independent external/temporal evaluation identified	4-fold cross-validation	Calibration plot/curve; Brier scores reported for EuroSCORE Logistic, CAPRI, and eCAPRI	Not reported	Not reported	Not reported	No — retrospective	No — clinical outcome cohort single-centre; segmentation data multisite	Not reported	Univariate screening	Not reported/NA	Not reported

Supplementary Table S4. PROBAST+AI-aligned risk-of-bias and applicability assessment

ID	Year	Overall evaluation RoB	Applicability	Participants/data	Predictors	Outcome	Analysis	Evaluation type
1	2019	Unclear	Unclear concern	Low	Unclear	Unclear	Unclear	Internal evaluation only
2	2021	High	Unclear concern	Unclear	Unclear	Unclear	High	Development/unclear evaluation
3	2023	Unclear	Unclear concern	Low	Unclear	Low	Low	Stronger external/temporal evaluation
4	2023	Unclear	Unclear concern	Low	Unclear	Low	Unclear	Internal evaluation only
5	2023	High	Unclear concern	Unclear	Unclear	Unclear	High	Internal evaluation only
6	2024	Unclear	Low concern	Low	Low	Low	Unclear	Internal evaluation only
7	2021	Unclear	Unclear concern	Low	Unclear	Low	Unclear	Internal evaluation only
8	2023	Unclear	Unclear concern	Low	Unclear	Low	Unclear	Internal evaluation only
9	2024	Unclear	Unclear concern	Low	Unclear	Low	Low	Stronger external/temporal evaluation
10	2024	High	Unclear concern	Low	Unclear	Unclear	High	Internal evaluation only
11	2019	Unclear	Unclear concern	Low	Unclear	Low	Unclear	Internal evaluation only
12	2021	Unclear	Unclear concern	Low	Unclear	Low	Unclear	Internal evaluation only
13	2023	Unclear	Low concern	Low	Unclear	Low	Unclear	Stronger external/temporal evaluation
14	2021	Low	Low concern	Low	Low	Low	Low	Stronger external/temporal evaluation
15	2024	High	Unclear concern	Low	Unclear	Low	High	Internal evaluation only
16	2020	High	Low concern	Low	High	Low	High	Internal evaluation only
17	2022	Unclear	Unclear concern	Low	Unclear	Low	Unclear	Internal evaluation only
18	2021	High	Unclear concern	Low	Unclear	Unclear	High	Internal evaluation only
19	2023	High	Unclear concern	Low	Unclear	Unclear	High	Internal evaluation only
20	2023	Unclear	Low concern	Low	Low	Low	Unclear	Internal evaluation only
21	2022	Unclear	Unclear concern	Low	Unclear	Low	Unclear	Internal evaluation only
22	2023	Unclear	Low concern	Low	Unclear	Low	Unclear	Internal evaluation only
23	2023	High	Unclear concern	Low	Unclear	Low	High	Development/unclear evaluation
24	2021	High	Low concern	Low	Low	Low	High	Internal evaluation only
25	2021	Unclear	Unclear concern	Low	Unclear	Low	Unclear	Internal evaluation only
26	2021	High	Unclear concern	Unclear	Unclear	Low	High	Internal evaluation only
27	2020	Unclear	Low concern	Low	Low	Low	Unclear	Internal evaluation only
28	2023	Unclear	Unclear concern	Low	Unclear	Low	Unclear	Internal evaluation only
29	2020	High	Low concern	Low	Unclear	Low	High	Internal evaluation only
30	2021	Unclear	Unclear concern	Low	Unclear	Low	Unclear	Stronger external/temporal evaluation
31	2021	Low	Low concern	Low	Low	Low	Low	Stronger external/temporal evaluation
32	2022	Low	Low concern	Low	Low	Low	Low	Stronger external/temporal evaluation
33	2022	Unclear	Low concern	Low	Unclear	Unclear	Unclear	Internal evaluation only
34	2023	High	Low concern	Low	Low	Unclear	High	Internal evaluation only
35	2024	Unclear	Low concern	Low	Low	Low	Unclear	Internal evaluation only
36	2024	Unclear	Unclear concern	Low	Unclear	Unclear	Unclear	Internal evaluation only
37	2024	High	Unclear concern	Low	Unclear	Unclear	High	Development/unclear evaluation
38	2025	Unclear	Low concern	Low	Low	Low	Unclear	Internal evaluation only
39	2025	High	Unclear concern	Low	Unclear	Unclear	High	Internal evaluation only
40	2025	Unclear	Low concern	Low	Low	Low	Unclear	Internal evaluation only
41	2024	High	Unclear concern	High	Unclear	Low	High	Internal evaluation only
42	2025	High	Unclear concern	Low	Unclear	Unclear	High	Development/unclear evaluation
43	2025	Unclear	Unclear concern	Low	Unclear	Unclear	Unclear	Internal evaluation only
44	2025	Unclear	High concern	Low	Unclear	Unclear	Unclear	Internal evaluation only
45	2025	Unclear	Low concern	Low	Low	Low	Unclear	Internal evaluation only

46	2025	Unclear	Unclear concern	Low	Unclear	Low	Unclear	Internal evaluation only
47	2025	Unclear	Low concern	Low	Low	Low	Unclear	Stronger external/temporal evaluation
48	2025	Unclear	Low concern	Low	Low	Low	Unclear	Internal evaluation only
49	2025	High	Low concern	Low	Unclear	Unclear	High	Internal evaluation only
50	2025	Unclear	Low concern	Low	Low	Low	Unclear	Internal evaluation only
51	2026	Low	Low concern	Low	Low	Low	Low	Stronger external/temporal evaluation
52	2026	Unclear	Low concern	Low	Low	Low	Unclear	Internal evaluation only
53	2026	High	High concern	Low	Low	Low	High	Stronger external/temporal evaluation
54	2026	High	Low concern	Low	Low	Low	High	Internal evaluation only
55	2026	Unclear	Low concern	Low	Low	Low	Unclear	Stronger external/temporal evaluation
56	2026	High	Low concern	Low	Unclear	Unclear	High	Development/unclear evaluation
57	2026	High	Low concern	Low	Unclear	Unclear	High	Internal evaluation only
58	2026	Low	Low concern	Low	Low	Low	Low	Stronger external/temporal evaluation
59	2026	Unclear	Unclear concern	Low	Low	Unclear	Low	Stronger external/temporal evaluation
60	2026	High	Low concern	Low	Low	Low	High	Internal evaluation only
61	2026	Unclear	High concern	Low	Low	Low	Unclear	Internal evaluation only
62	2025	Low	Low concern	Low	Low	Low	Low	Stronger external/temporal evaluation
63	2026	Unclear	High concern	Low	Low	Unclear	Low	Stronger external/temporal evaluation
64	2026	Unclear	Unclear concern	Low	Low	Unclear	Unclear	Stronger external/temporal evaluation
65	2026	Low	High concern	Low	Low	Low	Low	Stronger external/temporal evaluation
66	2026	Low	Low concern	Low	Low	Low	Low	Stronger external/temporal evaluation
67	2026	High	Low concern	Low	Low	Low	High	Internal evaluation only

AUC magnitude was not considered in assigning risk-of-bias judgments. The assessment is aligned with PROBAST+AI concepts and focuses on credibility of performance evaluation.

Supplementary Table S5. Primary mortality meta-analysis inputs and performance provenance

Study	AUC (95% CI)	Precision provenance	Validation	Predictor timing	Horizon	Reconstructed precision
Mamprin et al., 2021	0.680 (0.602-0.749)	existing	external inter-centre	preprocedural	1 year	Yes
Gomes et al., 2021	0.970 (0.950-0.980)	existing	internal nested CV	peri/post/mixed	early/in-hospital	No
Agasthi et al., 2021	0.720 (0.680-0.760)	existing	internal repeated CV	preprocedural	1 year	No
Penso et al., 2021	0.790 (0.745-0.829)	existing	internal stratified CV	preprocedural	>1 year	Yes
Alhwiti et al., 2023	0.810 (0.768-0.846)	existing	temporal holdout	preprocedural	early/in-hospital	Yes
Yordanov et al., 2024	0.680 (0.660-0.700)	existing	geographic leave-centre-out	preprocedural	early/in-hospital	No
Asif et al., 2024	0.760 (0.478-0.916)	existing	held-out test	peri/post/mixed	early/in-hospital	Yes
Lertsanguansinchai et al., 2023	0.710 (0.600-0.810)	existing	internal repeated split/CV	preprocedural	1 year	No
Stan et al., 2023	0.916 (0.849-0.955)	existing	internal 5-fold CV	preprocedural	>1 year	Yes
Pollari et al., 2023	0.810 (0.737-0.866)	existing	internal 10-fold CV	peri/post/mixed	1 year	Yes
Bruggemann et al., 2024	0.725 (0.698-0.751)	existing	internal stratified CV	preprocedural	>1 year	Yes
Leha et al., 2023	0.780 (0.740-0.820)	existing	temporal/generalisation test	peri/post/mixed	early/in-hospital	No
Savitz et al., 2023	0.600 (0.490-0.700)	HM from whole cohort; 5-fold OOF	internal 5-fold CV	preprocedural	early/in-hospital	Yes
Kreutz et al., 2025	0.794 (0.699-0.888)	reported validation CI	held-out validation cohort	preprocedural	1 year	No
Kwiecinski et al., 2025	0.790 (0.740-0.840)	reported external CI	external multicentre	preprocedural	1 year	No
He et al., 2025	0.915 (0.870-0.950)	reported bootstrap 95% CI of OOF CV	internal 5-fold OOF CV	preprocedural	early/in-hospital	No
Hsiao et al., 2026	0.740 (0.670-0.800)	reported external CI	external prospective TAVR cohort	preprocedural	1 year	No
Bharucha et al., 2026	0.840 (0.746-0.904)	HM from external validation n/event rate	external independent centre	preprocedural	1 year	Yes
Shi et al., 2026	0.712 (0.627-0.795)	reported external CI	external independent centre	preprocedural	>1 year	No
Lartaud et al., 2026	0.731 (0.575-0.845)	HM from held-out evaluation n/events	held-out evaluation	preprocedural	1 year	Yes

Supplementary Table S6. Mortality subgroup and meta-regression results

Dimension	Level/term	k	Estimate	I²	Prediction interval / P	Analysis
validation	stronger	8	0.754 (0.701-0.800)	86.6%	0.596-0.864	Subgroup estimate
validation	internal	12	0.814 (0.716-0.884)	92.9%	0.390-0.968	Subgroup estimate
timing	preprocedural	16	0.767 (0.713-0.813)	86.2%	0.544-0.900	Subgroup estimate
timing	peri/post/mixed	4	0.866 (0.520-0.975)	95.6%		Subgroup estimate
horizon	early/in-hospital	7	0.826 (0.639-0.927)	96.7%	0.217-0.988	Subgroup estimate
horizon	1 year	9	0.755 (0.711-0.793)	49.8%	0.646-0.838	Subgroup estimate
horizon	>1 year	4	0.793 (0.578-0.915)	86.3%		Subgroup estimate
validation	validation contrast	-	β=-0.336 (-0.997 to 0.325)	-	P = 0.299	Meta-regression
timing	timing contrast	-	β=0.696 (-0.101 to 1.494)	-	P = 0.083	Meta-regression
horizon	1-year vs early	-	β=-0.401 (-1.162 to 0.361)	-	P = 0.282	Meta-regression
horizon	>1-year vs early	-	β=-0.197 (-1.134 to 0.740)	-	P = 0.663	Meta-regression
small-study/SE	SE moderator	-	β=1.534 (-1.353 to 4.422)	-	P = 0.279	Meta-regression

Meta-regression coefficients are on the logit-AUC scale. Moderator analyses are exploratory study-level associations and should not be interpreted causally.

Supplementary Table S7. Dependence-aware algorithm-family meta-regression

Algorithm family	Effects	Adjusted AUC (95% CI)	Effective df	P vs LR	Delta AUC vs LR
LR	5	0.787 (0.718-0.843)	13.459		
Boosting	7	0.796 (0.732-0.848)	11.568	0.415	0.009
Neural/deep	8	0.798 (0.726-0.856)	13.796	0.424	0.011
RF	4	0.813 (0.699-0.891)	7.586	0.335	0.026
SVM	3	0.765 (0.699-0.820)	7.355	0.218	-0.022

CR2 cluster-robust variance estimation accounted for multiple algorithm estimates within the same source/report family. Non-significant contrasts do not establish equivalence.

Supplementary Table S8. Audited early-mortality threshold-level operating points

Analysis	Study	Outcome	Model	TP	FN	TN	FP	Sensitivity	Specificity	Provenance note
representative	Alhwiti 2023 (F8)	In-hospital mortality	L2 logistic regression, temporal holdout	109	67	11680	2126	0.619	0.846	Counts algebraically reconstructed from N=13,982 and rounded sens/spec/PPV/NPV.
representative	Gomes 2021 (F16)	In-hospital mortality	Random forest, all features	24	1	383	43	0.960	0.899	N=451, 25 deaths; sensitivity 0.96 at prespecified specificity 0.90.
representative	Leha 2023 (F22)	30-day mortality	TRIMpost, temporal GARY test set	106	40	4146	1572	0.726	0.725	Counts reconstructed from N=5,864 and reported sens/spec/PPV/NPV.
representative	Hsiao 2026 (F58)	30-day mortality	AI-ECG T-score, external TAVR cohort	12	0	275	152	1.000	0.644	N=439; 30-day mortality 2.7%; threshold 1.04, sensitivity 1.00, specificity 0.644.
Preprocedural only	Alhwiti 2023 (F8)	In-hospital mortality	L2 logistic regression, preoperative inputs	109	67	11680	2126	0.619	0.846	Same temporal holdout operating point; inputs are preoperative.
Preprocedural only	Gomes 2021 (F16)	In-hospital mortality	Random forest, baseline-only	10	15	383	43	0.400	0.899	Baseline-only RF; sensitivity 0.40 at specificity 0.90.
Preprocedural only	Leha 2023 (F22)	30-day mortality	TRIMpre, temporal GARY test set	101	71	4494	1260	0.587	0.781	Counts reconstructed from N=5,926 and reported sens/spec/PPV/NPV.
Preprocedural only	Hsiao 2026 (F58)	30-day mortality	AI-ECG T-score, preprocedural ECG	12	0	275	152	1.000	0.644	Same external preprocedural AI-ECG operating point.

Threshold-level analyses were exploratory. Reconstructed cell counts are explicitly identified; operating points were included only when model, outcome, horizon, evaluation cohort, and threshold definition could be linked.

Supplementary Table S9. Mortality specification and sensitivity analyses

Analysis	k	AUC (95% CI)	tau²	I²	Prediction interval	Purpose
Primary	20	0.791 (0.732-0.840)	0.428	91.5%	0.480-0.940	Reference
Reported CI only	10	0.805 (0.693-0.883)	0.661	94.7%	0.365-0.967	Remove reconstructed precision
Reconstructed SE ×1.25	20	0.791 (0.731-0.840)	0.424	90.7%	0.481-0.939	Precision stress-test
Reconstructed SE ×1.5	20	0.791 (0.731-0.841)	0.421	90.2%	0.482-0.939	Precision stress-test
Reconstructed SE ×2.0	20	0.791 (0.729-0.842)	0.421	89.7%	0.482-0.939	Precision stress-test
Exclude Gomes et al., 2021	19	0.769 (0.725-0.808)	0.170	84.9%	0.575-0.891	Influence/timing sensitivity
Exclude He et al., 2025	19	0.782 (0.722-0.832)	0.391	90.9%	0.480-0.933	Influence/timing sensitivity
Exclude Gomes et al., 2021 + He et al., 2025	18	0.757 (0.718-0.791)	0.100	81.5%	0.608-0.862	Influence/timing sensitivity
Preprocedural only	16	0.767 (0.713-0.813)	0.207	86.2%	0.544-0.900	Influence/timing sensitivity
Kwiecinski et al., 2025 family swap	20	0.793 (0.734-0.841)	0.430	91.7%	0.481-0.940	Alternate overlapping-family representative
Add Hoffmann et al. (uncertain precision)	21	0.786 (0.728-0.834)	0.423	91.1%	0.476-0.937	Boundary inclusion sensitivity
Alternate earliest horizon for multi-horizon reports	20	0.797 (0.737-0.846)	0.450	91.7%	0.480-0.944	Source/horizon audit
Bharucha et al., 2026 caption-transposition sensitivity	20	0.789 (0.730-0.838)	0.422	91.4%	0.479-0.938	Source/horizon audit
Amsterdam/Catharina family swap to Lopes et al., 2019	20	0.792 (0.733-0.840)	0.422	91.6%	0.483-0.939	Source/horizon audit
Netherlands registry family swap to Lopes et al., 2023	20	0.790 (0.729-0.840)	0.443	89.5%	0.471-0.941	Source/horizon audit
Both source-family swaps	20	0.790 (0.730-0.840)	0.437	89.9%	0.475-0.940	Source/horizon audit

Risk-of-bias restriction *(not counted among the 16 predefined analytic specifications above): excluding all five high-risk mortality studies leaves k=15 low/unclear-risk studies, with pooled AUC 0.747 (95% CI, 0.713-0.779). This is exactly the complement of the prespecified five-study high-risk subgroup and therefore reproduces the prespecified low/unclear-risk subgroup estimate. Tau², I², and a new prediction interval were not separately re-estimated for this reviewer-requested restriction and are intentionally not reported.*

Supplementary Figures

Supplementary Figure S1. Strictly audited clinical-readiness milestones across the 67-report corpus. Counts require explicit full-text evidence under the prespecified definition.

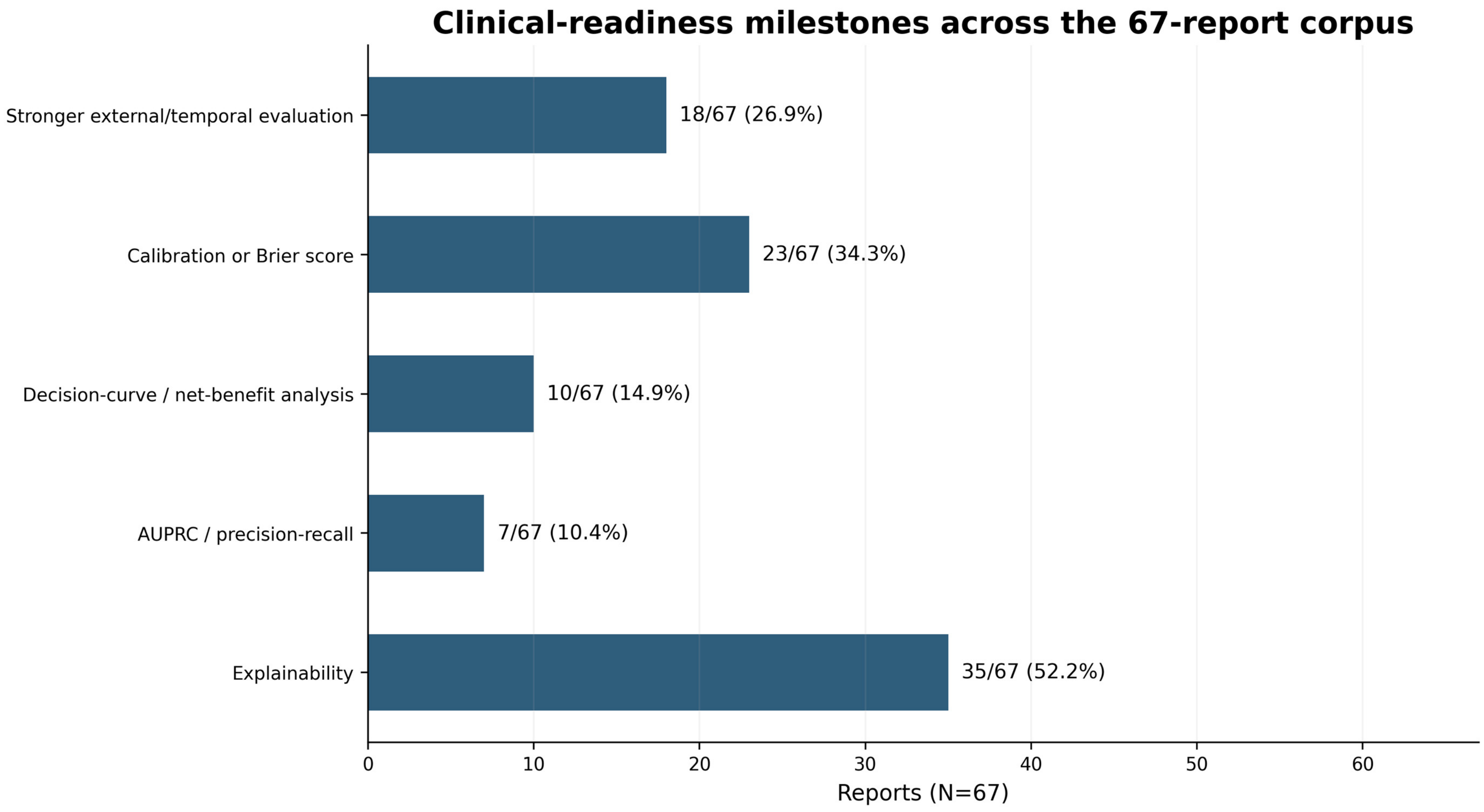

Supplementary Figure S2. Report-level matrix of five strictly audited clinical-readiness/reporting signals. Filled cells denote explicit full-text evidence; prospective and multicentre data-source descriptors are reported separately in Supplementary Tables S2–S3.

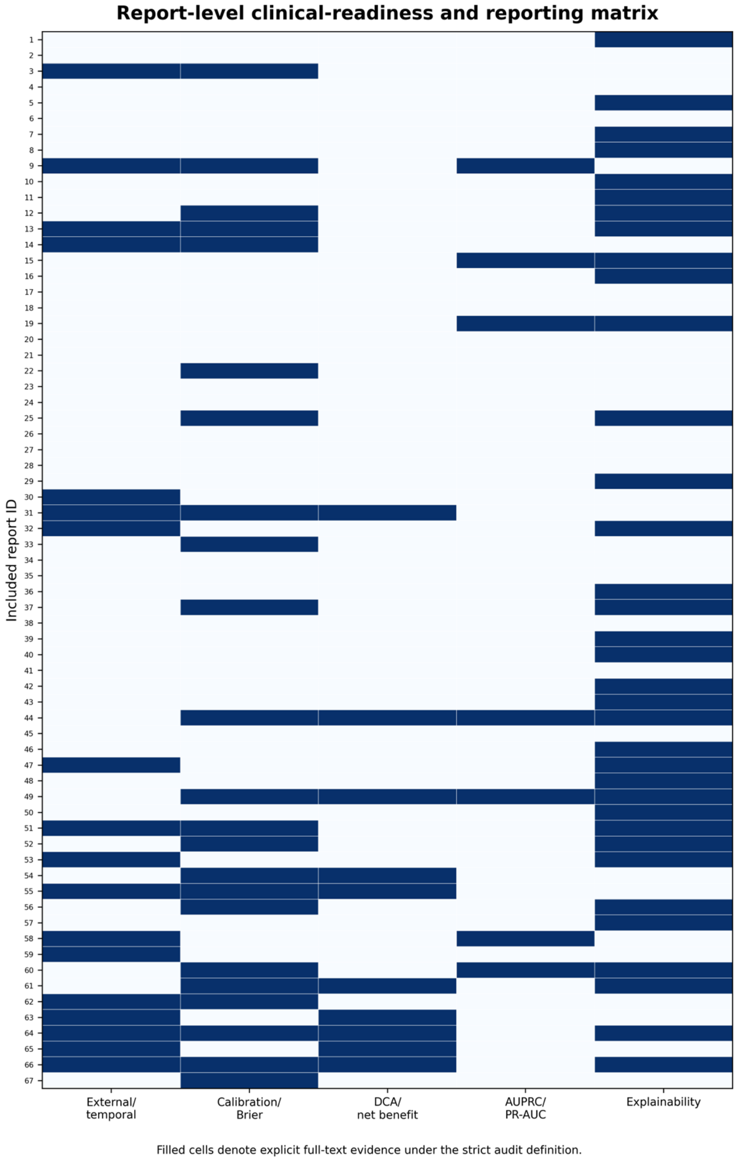

Supplementary Figure S3. Drapery plot for the primary mortality synthesis.

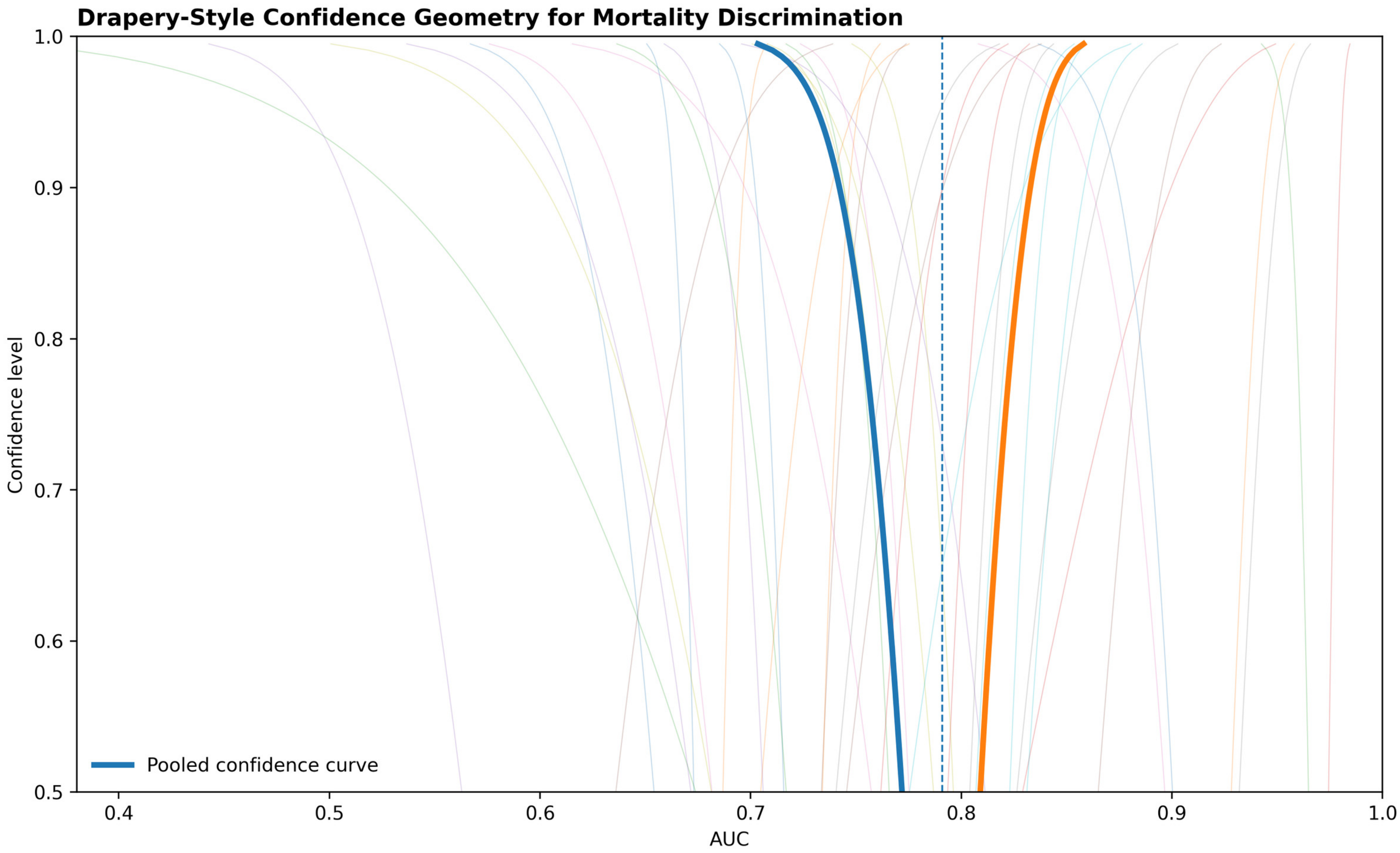

Supplementary Figure S4. Mortality subgroup estimates by data modality and source type.

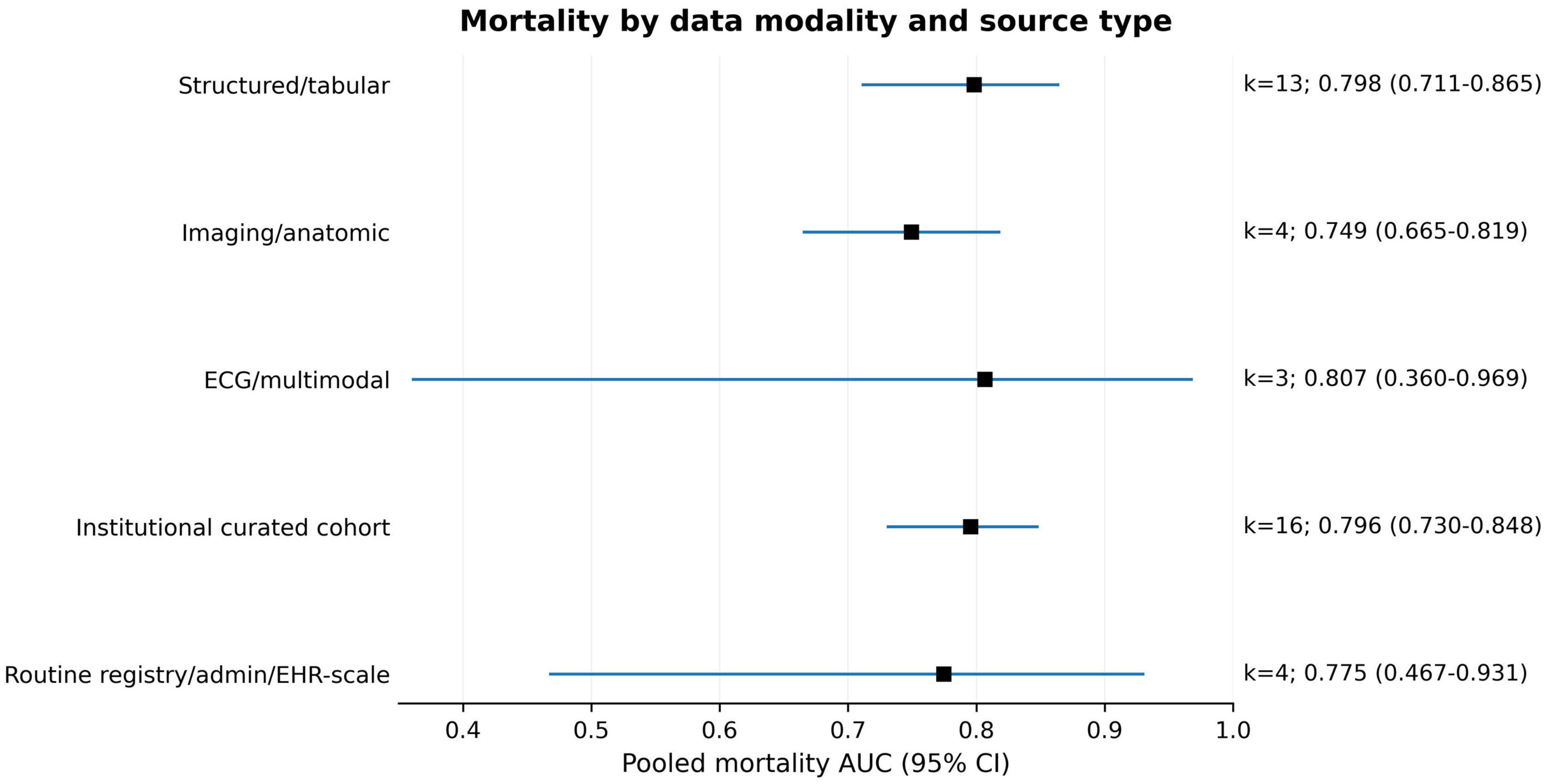

Supplementary Figure S5. Evaluation-sample-size and event-prevalence meta-regression diagnostics. Both are exploratory study-level analyses.

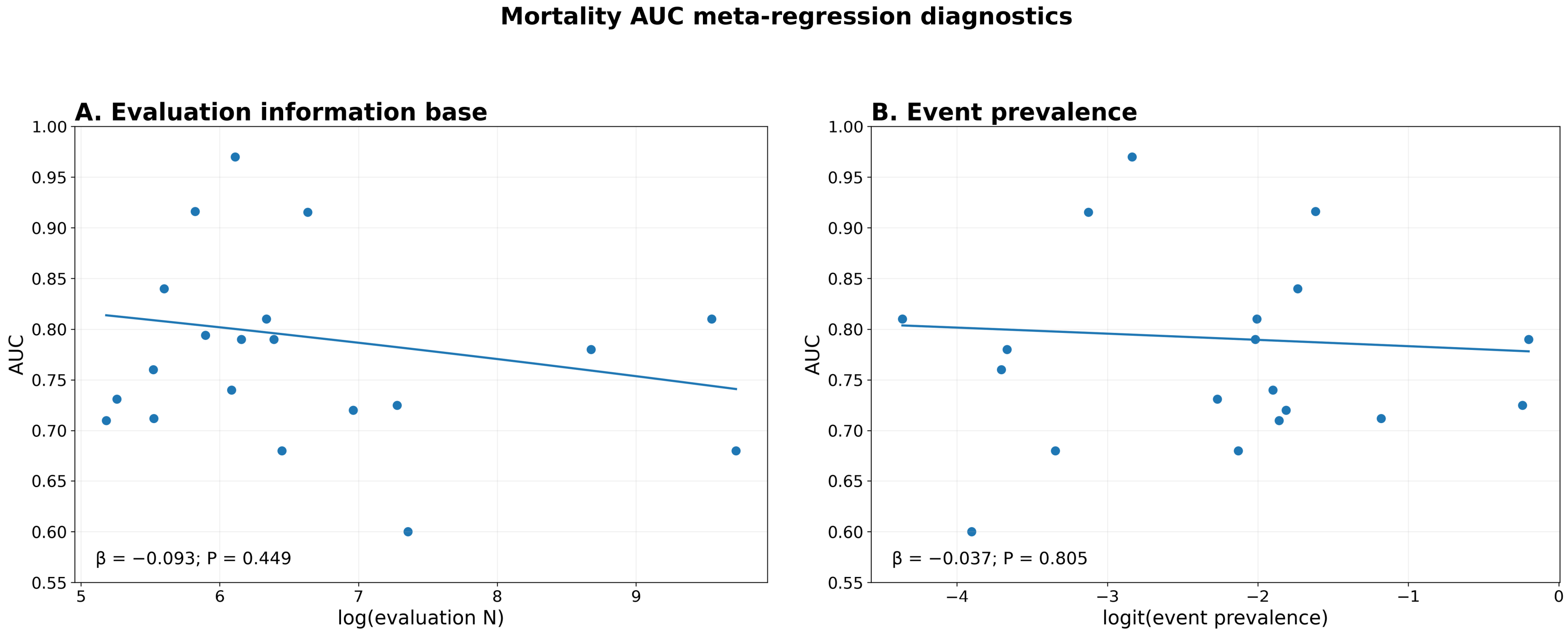

Meta-regression coefficients are on the logit-AUC scale.

Supplementary Figure S6. Study-level PROBAST+AI-aligned evaluation risk-of-bias traffic-light assessment across the 67 included reports.

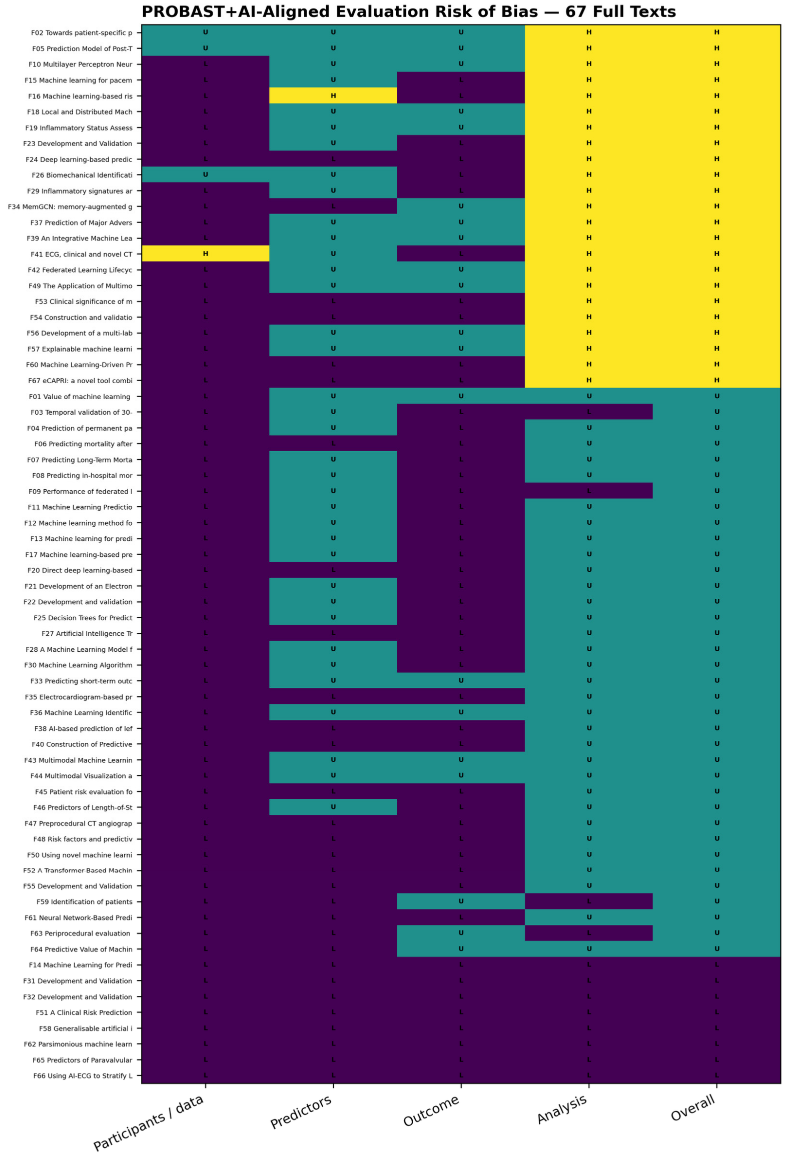

Supplementary Figure S7. Source-audited mortality design-quality multiverse. The figure retains univariable comparisons with consistently defined full-text coding (evaluation risk of bias, calibration reporting, and missing-data reporting). Ambiguously defined report-level prospective and multicentre modifiers were not retained as inferential moderators. Exploratory.

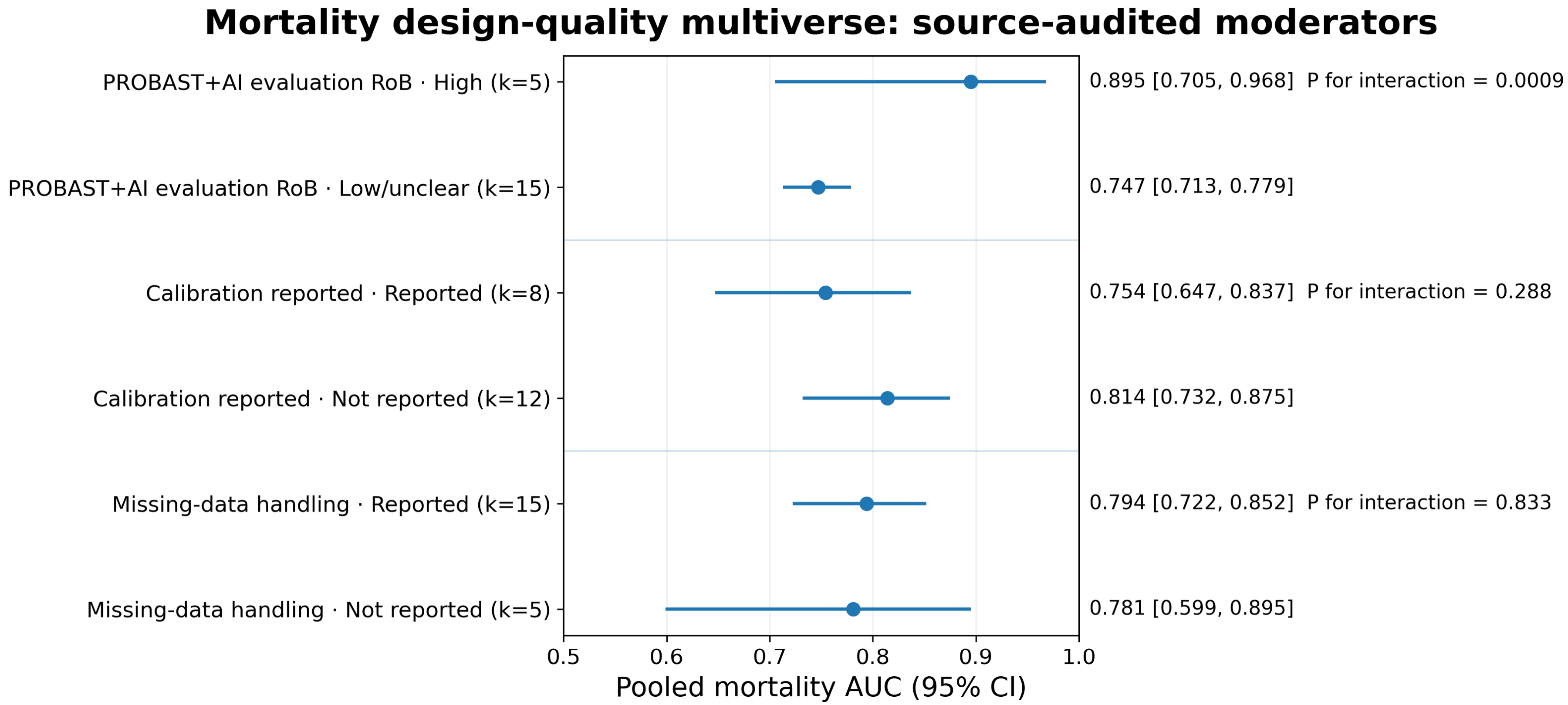

Univariable exploratory comparisons. Ambiguously defined report-level prospective/multicentre modifiers were not retained as inferential moderators.

Supplementary Figure S8. Regularized Bayesian meta-regression coefficients for high evaluation risk of bias, stronger validation, and predictor timing. The adjusted marginal risk-of-bias contrast is displayed in Main Figure 5C.

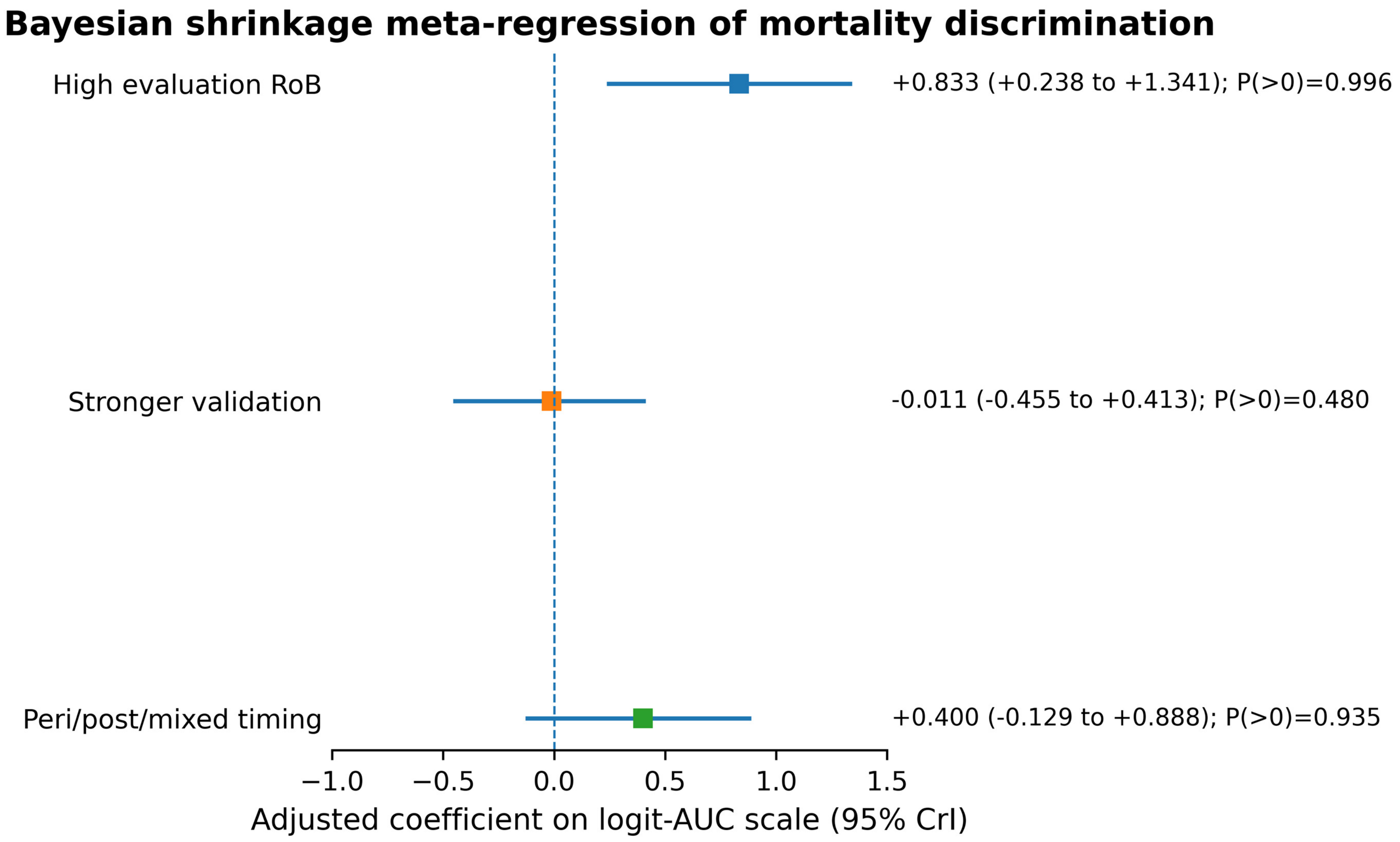

Supplementary Figure S9. Outcome-specific forest plots: strict permanent pacemaker implantation and exploratory significant/residual paravalvular leak. The residual-PVL deep-learning study is identified as Yao et al., 2026; its retained AUC precision uses the audited Stage-2 evaluation information base.

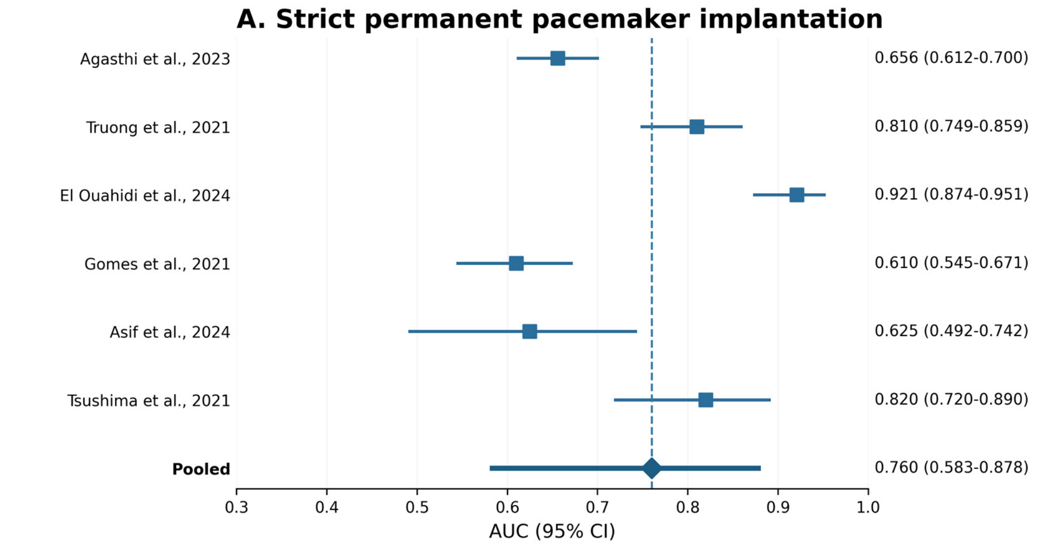

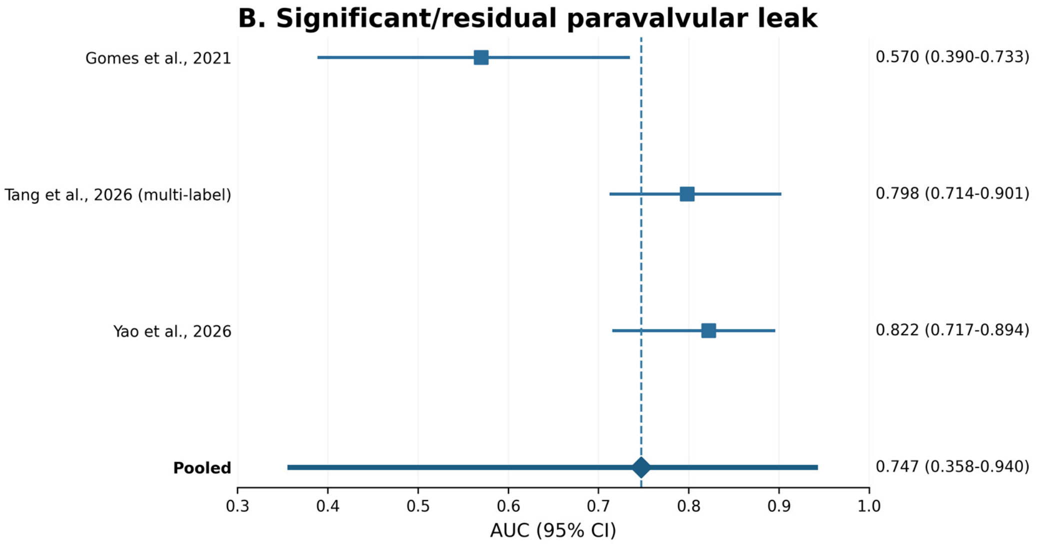

Supplementary Figure S10. Exploratory early-mortality HSROC analyses: representative operating points and preprocedural-only sensitivity analysis.

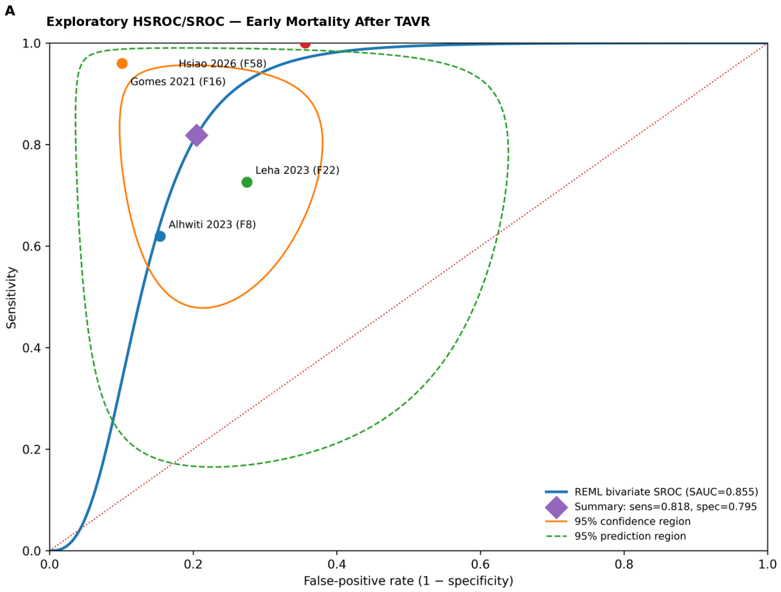

k=4 independent reports; in-hospital or 30-day mortality. Operating points are directly reported or algebraically reconstructed from rounded metrics. Use as an exploratory supplementary analysis because k is small and model/prediction timing differs.

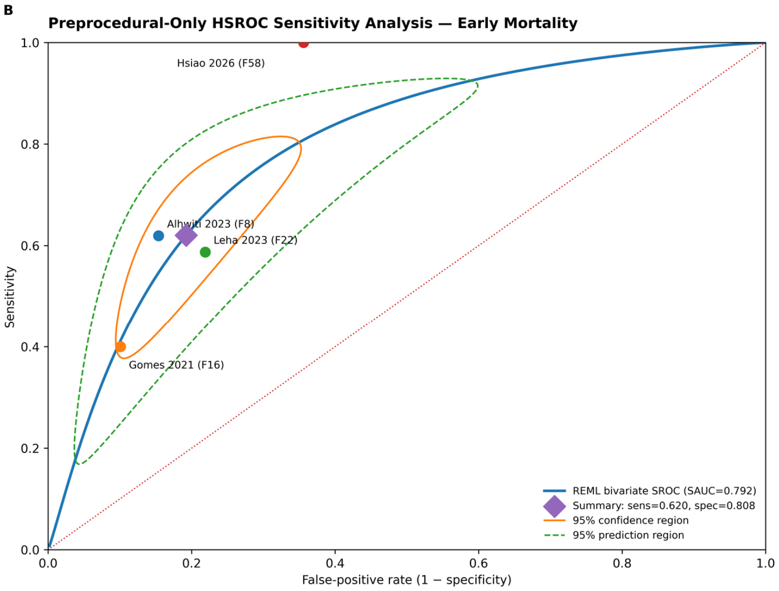

Same four independent reports, restricting to baseline/preprocedural model variants where available. This timing-restricted analysis remains exploratory because k=4 and in-hospital/30-day horizons are combined.

Supplementary Figure S11. Leave-one-out robustness of the primary mortality meta-analysis. Study labels are normalized to author-year format; each estimate is the pooled AUC after excluding the indicated study.

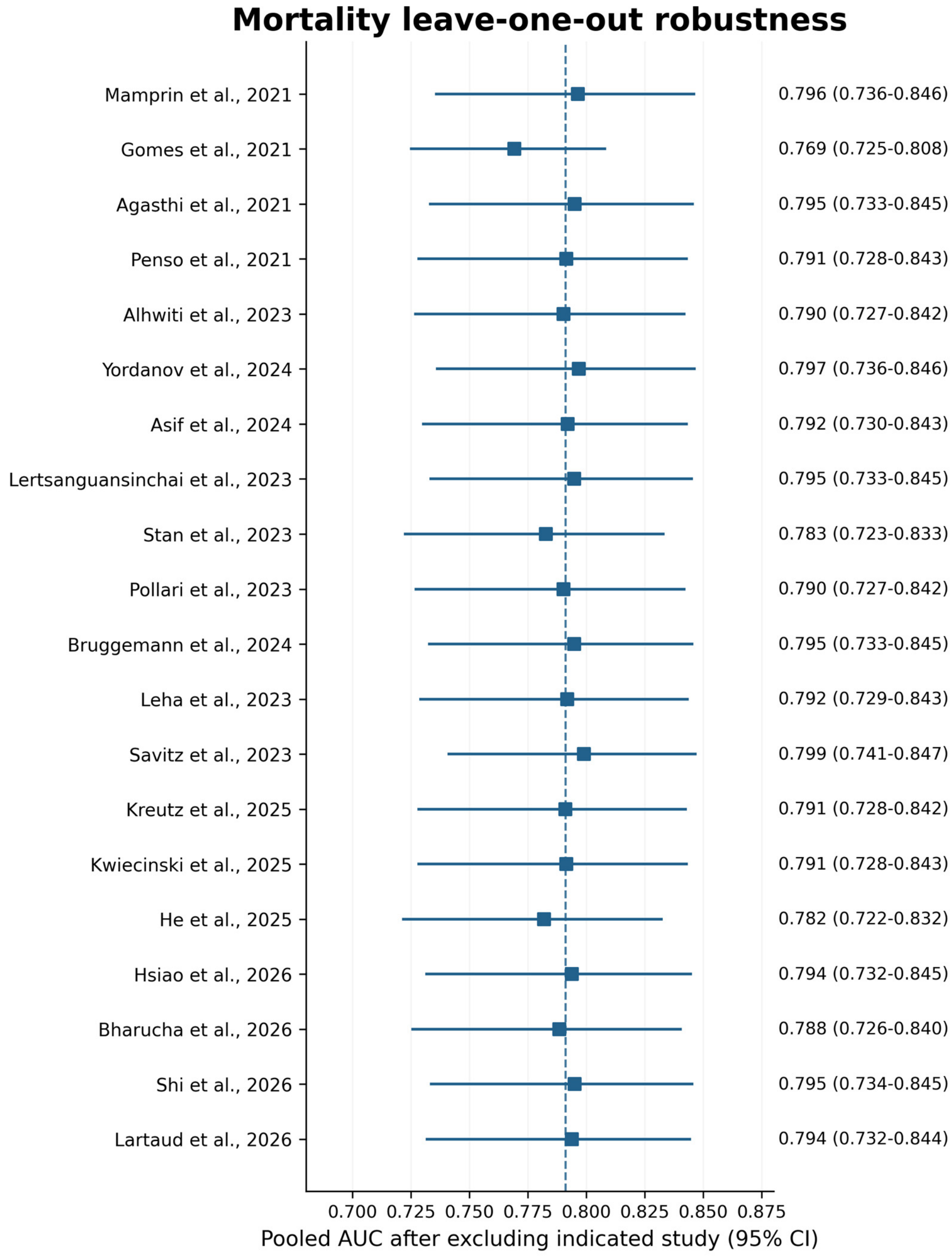

Supplementary Figure S12. Influence diagnostic for the primary mortality synthesis. Study labels are normalized to author-year format; influence was used diagnostically rather than as an automatic exclusion criterion.

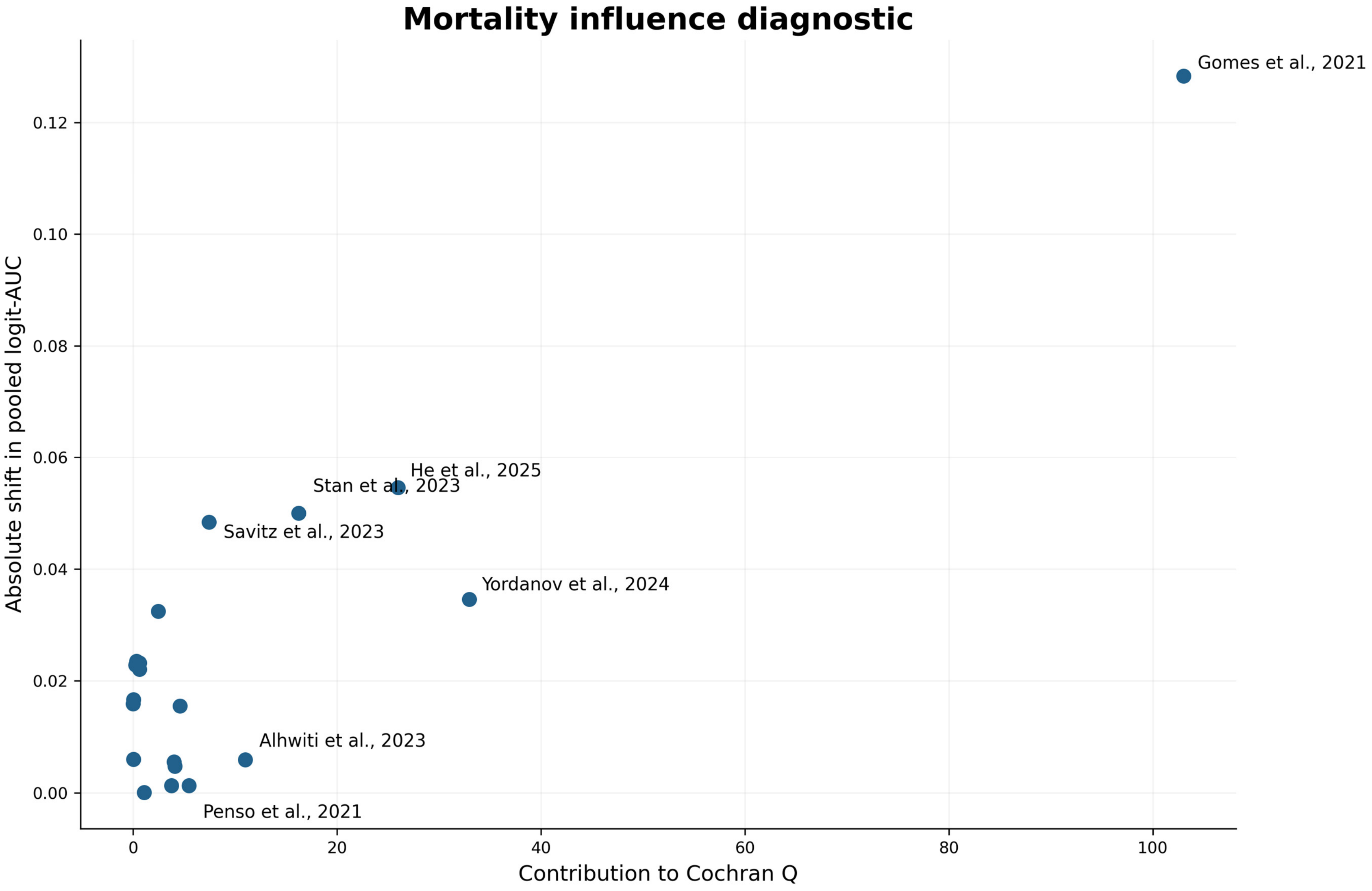

Supplementary Figure S13. Selected mortality sensitivity and specification analyses. Source-family and influential-study scenario labels are normalized to the corresponding author-year reports.

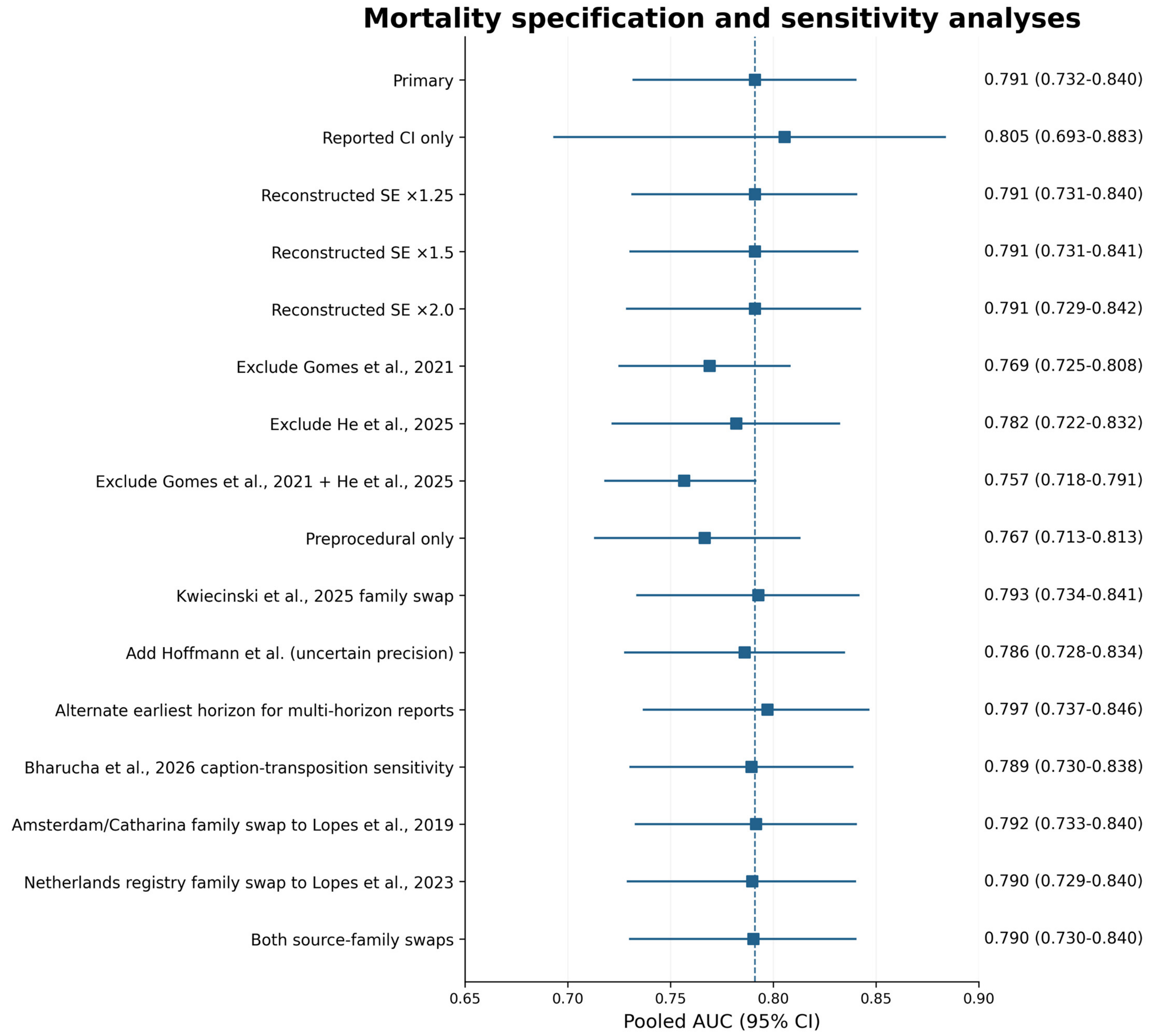

Supplementary Figure S14. Sensitivity of the primary mortality estimate to heterogeneity estimator and inferential method.

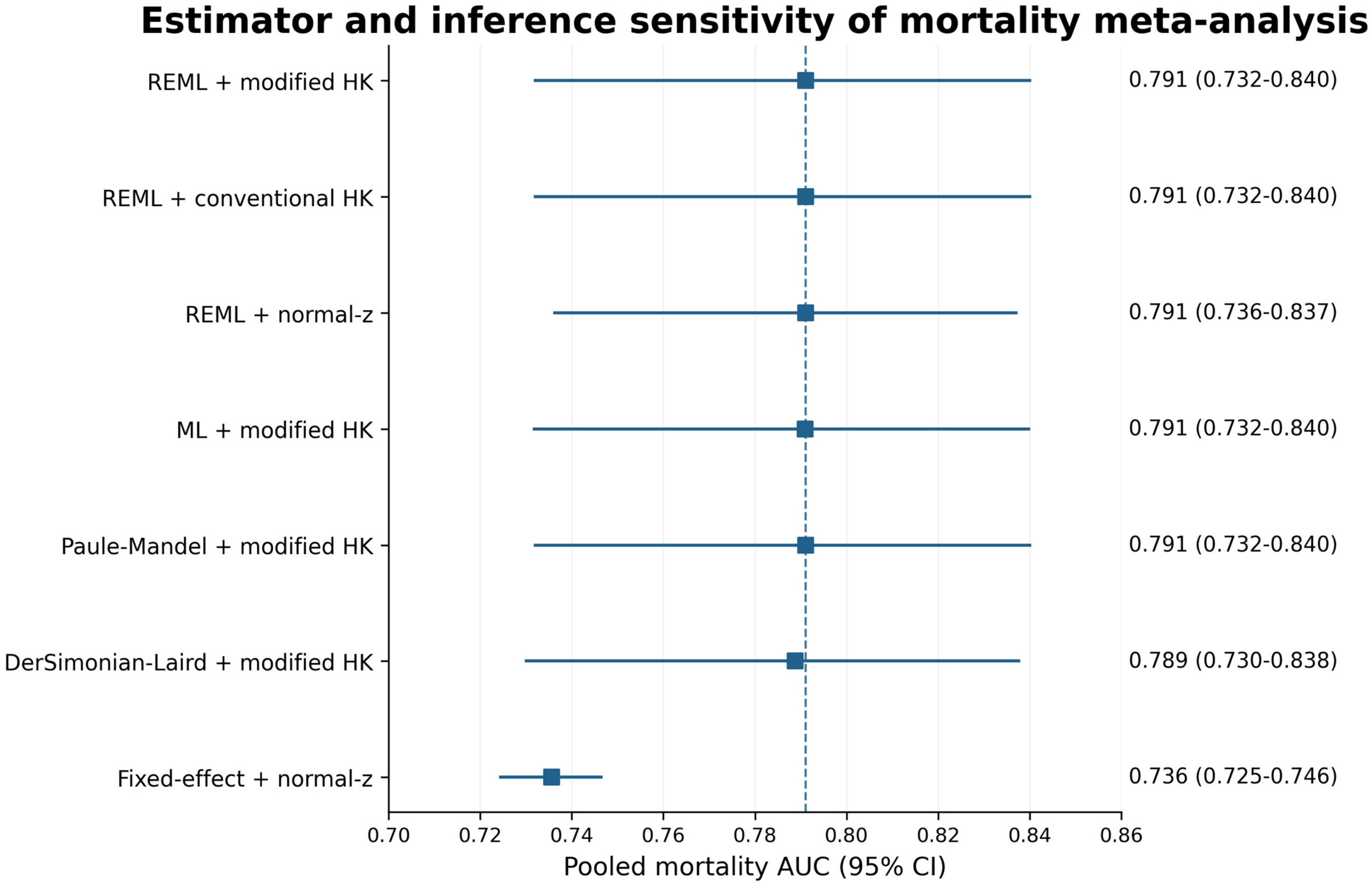

Supplementary Figure S15. GOSH landscape based on 25,000 random subsets of the final mortality evidence set; used to examine heterogeneity geometry rather than to replace primary REML inference.

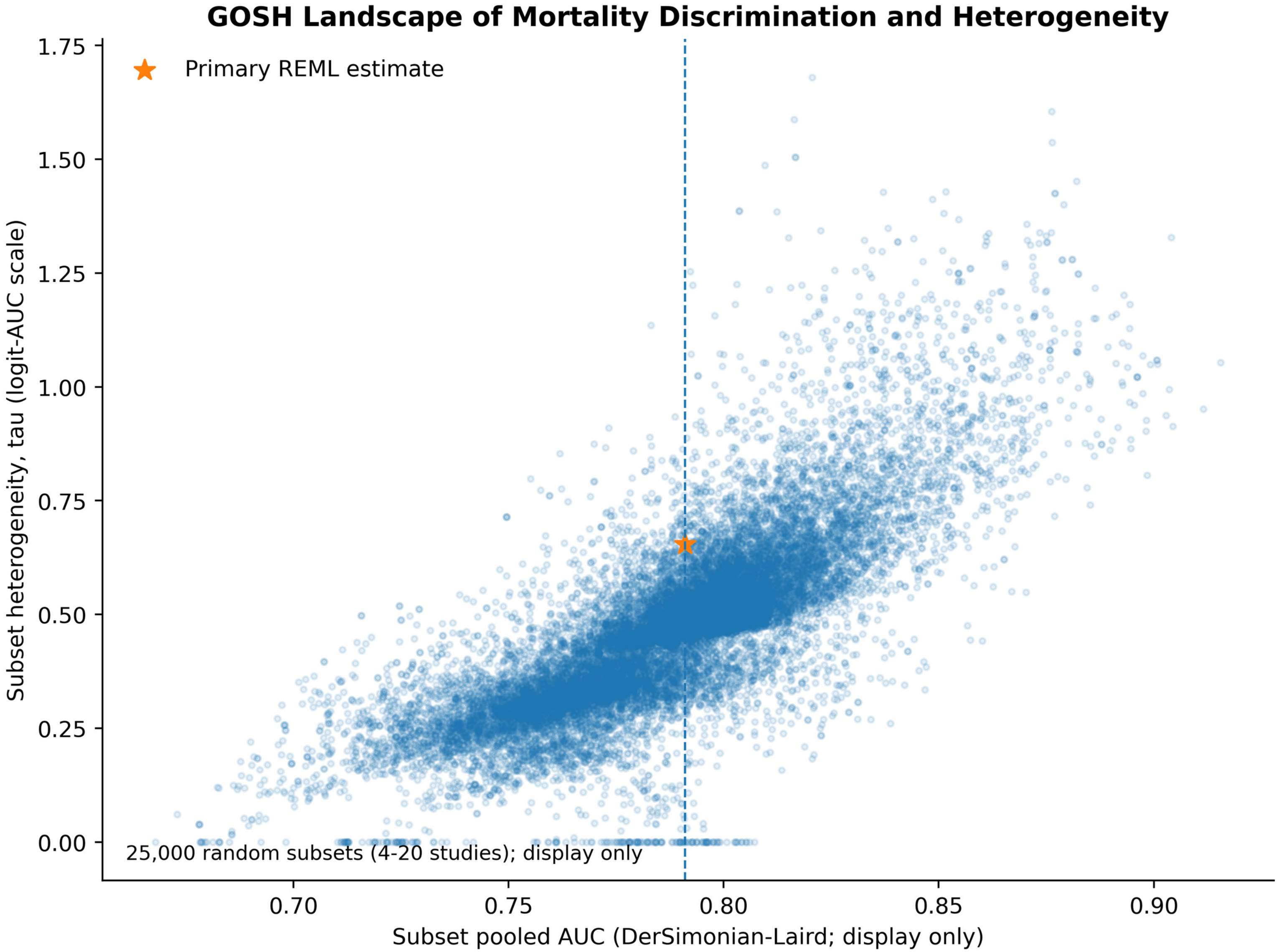

Supplementary Figure S16. Contour-enhanced mortality funnel plot. Asymmetry was interpreted cautiously and not as direct proof of publication bias.

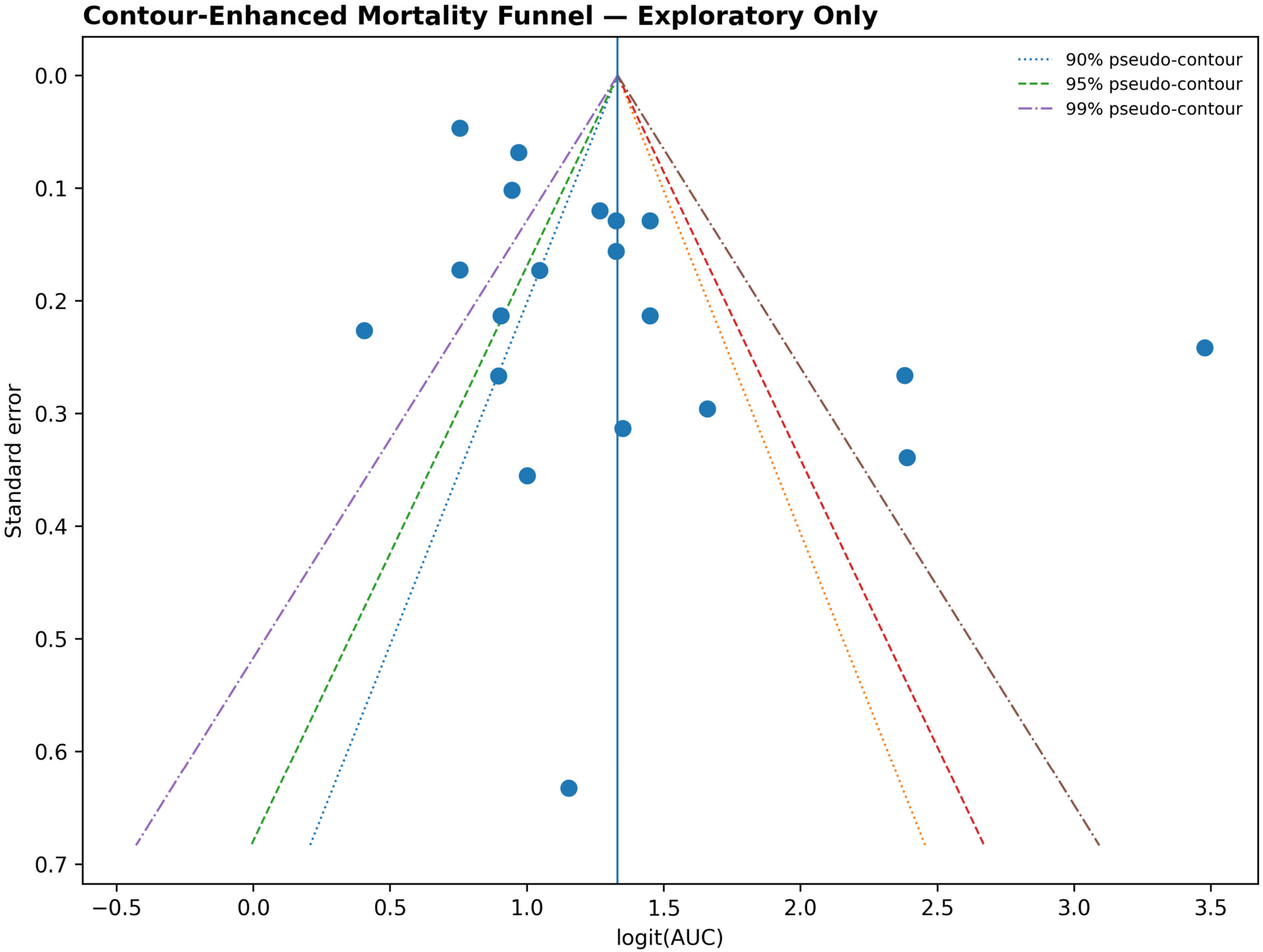

Supplement: Supplementary file 1 [file jcm-15-07692-s001.zip › Supplementary Tables and Figures.pdf]
